# eHealth and Hypertensive Disorders of Pregnancy: Systematic Review

**DOI:** 10.2196/77064

**Published:** 2025-09-10

**Authors:** Hillary Hu, Nargis Noori, Vincent Lee, Clara Chow, Ngai Wah Cheung, Kanchana Ekanayake, Monica Zen

**Affiliations:** 1 Faculty of Medicine, The University of Sydney, Sydney, Australia; 2 Westmead Applied Research Centre, The University of Sydney, Sydney, Australia; 3 Department of Obstetrics and Gynaecology, Westmead Hospital, Corner of Darcy and Hawkesbury Roads, Sydney, 2145, Australia, 61 88905555; 4 Centre for Kidney Research, School of Public Health, The University of Sydney, Sydney, Australia; 5 Westmead Hospital, Sydney, Australia; 6 Centre for Diabetes and Endocrinology Research, University of Sydney, Westmead Hospital, Sydney, Australia; 7 University of Sydney Library, The University of Sydney, Sydney, Australia

**Keywords:** blood pressure, digital health, hypertension, preeclampsia, pregnancy, telehealth, SMS text messages, mobile phone

## Abstract

**Background:**

Hypertensive disorders of pregnancy (HDP) affect up to 10% of pregnancies and can have adverse short and long-term implications for women and their babies. eHealth interventions include any health service or treatment delivered using the internet and related technology that aims to facilitate, capture, or exchange knowledge. eHealth interventions are increasingly used across many health care settings with improved outcomes.

**Objective:**

There have been no previous systematic reviews examining eHealth interventions and HDP. It is important to conduct this review as strategies to improve the monitoring and management of HDP can reduce morbidity, and potentially, mortality associated with HDP.

**Methods:**

We conducted a systematic review to examine all eHealth interventions targeted at patients at risk of or with HDP, the feasibility, acceptability of these interventions, and secondary outcomes, including clinical outcomes and resource utilization. The searches included two main concepts: eHealth interventions and HDP. Subject headings for the terms “telehealth,” “ehealth,” “digital health,” “telemedicine” and “preeclampsia,” “pregnancy induced hypertension,” “gestational hypertension,” and “high blood pressure” were used. The search was conducted on all papers published from the database inception to August 24, 2024. Meta-analyses of randomized controlled trial findings were conducted where possible. Other outcomes were reported in a narrative style with a summation of findings.

**Results:**

A total of 100 publications were identified with 61,539 participants. Interventions were primarily targeted at self-monitoring of blood pressure (BP) with reminders for BP checks, transmission of BP and HDP symptom data, and two-way communication between patients and care providers. In observational studies, there was no significant difference in clinical outcomes. Within qualitative outcomes, eHealth interventions appeared to be feasible, and all studies showed that participants were satisfied and found eHealth interventions easy to use. There was equivocal evidence regarding the cost benefits of eHealth interventions, but it did demonstrate largely reduced health care service utilization. In a meta-analysis of randomized controlled trial data, eHealth interventions reduced readmission rates (odds ratio [OR] 0.4, 95% CI 0.23-0.71), improved the likelihood of BP ascertainment (OR 7.02, 95% CI 4.41-11.15), and improved attendance at postpartum hypertension clinic (OR 1.44, 95% CI 0.98-2.12).

**Conclusions:**

The current evidence for the use of eHealth interventions targeted at patients at risk of or with HDP is of low quality and insufficient to make a recommendation regarding their routine use in clinical care. Our findings indicate that there is poor quality and low-level evidence that eHealth interventions are feasible, safe, and acceptable to patients. There is very limited evidence that it has the potential to reduce health care utilization, improve follow-up and BP ascertainment, reduce admissions, as well as confer some economic benefit compared to usual care with a generally positive patient experience with minimal patient concerns.

## Introduction

Preeclampsia and hypertensive disorders of pregnancy (HDP) affect up to 10% pregnancies [1], and can have adverse short and long-term implications for women, as well as potential impacts on offspring. Preeclampsia is characterized by hypertension in pregnancy after 20 weeks of gestation associated with proteinuria or other organ dysfunction. The constellation of hemolysis, elevated liver enzymes, low platelets syndrome is also a manifestation of preeclampsia [2]. Chronic hypertension (essential or secondary), gestational hypertension (hypertension without proteinuria or organ dysfunction in pregnancy), masked hypertension, and white coat hypertension encompass the other hypertensive disorders that can affect a pregnancy [2]. The pathophysiology of preeclampsia is multifactorial, with the primary theory of development being abnormal placentation leading to incomplete remodeling of the spiral arteries, narrow maternal vessels, and relative placental ischemia with tissue hypoxia causing endothelial damage resulting in hypertensive pathology [3,4]. Women with preeclampsia are more likely to develop cardiovascular disease, diabetes, and chronic kidney disease later in life [5,6], as well as have an increased risk of mortality during pregnancy and the puerperal period [7]. For the neonate, there is an increased risk of growth restriction, preterm birth, and stillbirth [7]. An estimated 15% of all preterm births are a result of preeclampsia [3].

eHealth and digital health interventions include any health service or treatment delivered using the internet and related technology that aims to facilitate, capture, or exchange knowledge [8,9]. eHealth interventions, including mobile phone SMS text messages, apps (mobile or computer-based), electronic monitors and wireless-enabled devices, audiovisual communication, and Bluetooth or web-based communication, can be used to complement and supplement conventional face-to-face clinician and patient interactions.

Technology-enhanced digital health care delivery can improve health outcomes, improve patient access to services [10-13], and has been reported to be feasible to implement and acceptable to patients with high patient satisfaction [14]. Within obstetrics, telehealth interventions have been shown to improve outcomes in smoking cessation and breastfeeding [10]. eHealth platforms that allow health care professionals to digitally monitor women at risk for or who have HDP can reduce antenatal visits, ultrasounds, and hypertension-related admissions [15]. A digital health platform for telemonitoring blood pressure (BP) and symptoms for women at increased risk of preeclampsia found high participant satisfaction and had significantly fewer admissions for hypertension and less antenatal visits and ultrasounds compared to women without telemonitoring [15].

Systematic reviews evaluating the impact of eHealth interventions compared to standard care report similar or improved results in managing chronic health conditions—for example, improving glycemic control [16] and improving cardiovascular risk factors [17]. However, there is insufficient evidence to determine the specific intervention and content to promote behavior change in different diseases, settings, and contexts. There have been no previous systematic reviews examining eHealth interventions and HDP. Given the current trend toward positive outcomes for the use of eHealth interventions in disease management and behavior modification, a review of the literature in HDP is justified. It is important to conduct this review as strategies to improve the monitoring and management of HDP can reduce morbidity, and potentially, mortality associated with HDP. As eHealth has the potential to enhance health care in HDP, it is important to determine the method and modality of interventions that are effective in improving outcomes in women with HDP.

Conducting clinical research on pregnant women is difficult due to ethical, logistical, and legal concerns [18]. As such, it is necessary to first confirm the feasibility and acceptability of eHealth interventions in patients with HDP prior to further research to establish positive clinical outcomes. This systematic review will examine all eHealth interventions targeted at patients at risk of or with HDP, the feasibility, and acceptability of these interventions, as well as secondary outcomes, including clinical outcomes and resource utilization.

## Methods

The systematic review was registered with the International Prospective Register of Systematic Reviews (CRD42023483948) and is reported as per the PRISMA (Preferred Reporting Items for Systematic Reviews and Meta‐Analyses) guidelines [19].

### Search Strategy

The search strategy included only terms relating to or describing the intervention. The searches included two main concepts: eHealth interventions and HDP. Subject headings for the terms “telehealth,” “ehealth,” “digital health,” “telemedicine” and “preeclampsia,” “pregnancy induced hypertension,” “gestational hypertension,” and “high blood pressure” were used. The full search strategy is attached in Multimedia Appendix 1. In cases where there were multiple publications reporting the same outcome, the complete peer-reviewed publication was included rather than conference proceedings or other shortened versions of the complete paper. In cases where there was significant overlap of results presented, but with some new data presented in the different publications, these were included and presented together.

### Information Sources

The search was conducted in the Cochrane Central Register of Controlled Trials (CENTRAL) through the Cochrane Register of Studies, MEDLINE Ovid, Embase Ovid, CINAHL, Web of Science, and Google Scholar databases. Reference lists of all primary studies, conference abstracts, and review studies were searched for additional references and trial registries for unpublished trial data. Authors were contacted for preprint data or trial data if required. Any identified gray literature was included if deemed relevant—searches were conducted in Google Scholar, and conference proceedings were included in the results and analysis.

### Eligibility Criteria

The search was conducted on all papers published from database inception to August 24, 2024, published in English. All study types, such as randomized controlled trials (RCTs; including cross-over RCTs, cluster RCTs, and quasi-RCTs), cohort studies, case-control studies, and observational studies, were included. We included studies from primary care and hospital settings.

We included all patients with risk factors for HDP, as well as those who had a diagnosis of HDP, according to established criteria. We included patients with any comorbidities, provided the digital intervention was aimed at the prevention or management of HDP. We excluded interventions that were targeted at health care professionals. Given that patient behavior change represents a potential barrier to optimal prevention of pregnancy complications, including preeclampsia [20], and the multitude of guidelines that direct clinician management of HDP, interventions targeted at patients have the potential to make a greater impact than those targeted at clinicians.

As well, most eHealth interventions are targeted at affecting patient behavior and attitudes; therefore, it is important to examine the effect of these interventions on patients first, prior to examining the impact on clinicians.

We studied outcomes including incidence of HDP, maternal morbidity including chronic hypertension (essential or secondary), gestational hypertension (hypertension without proteinuria or organ dysfunction in pregnancy), masked hypertension, white coat hypertension, preeclampsia, eclampsia, organ failure, hospital or intensive care unit admission and mortality arising from HDP, neonatal morbidity including preterm birth, growth restriction, and mortality, any adverse events, patient feedback and experience of the intervention, patient adherence to intervention, and rates of unscheduled antenatal presentations or emergency department presentations or hospital admissions.

### Study Selection

We included the following comparisons: (1) eHealth intervention versus non-eHealth intervention, (2) eHealth intervention versus an alternate eHealth intervention, and (3) eHealth interventions versus no intervention or usual care.

We included the following eHealth interventions [14]: (1) SMS text messages (eg, for reminders, education, prevention strategies, or management); (2) mobile phones, BP cuffs, and medical devices connected to phone by cord or wirelessly, or transmission of data by Bluetooth; (3) smartphone apps or apps on a smart device; (4) web or internet‐based interventions (eg, web-based training programs for patients and web-based transmission of monitoring data); (5) remote monitoring by health provider data collection at a different location from the patient, including store-and-forward (asynchronous) transmission of patient data through an electronic communication system; and (6) audiovisual interventions such as video.

We also included studies in which the intervention was part of a complex multicomponent integration care intervention.

All studies were assessed for eligibility in Covidence (Veritas Health Innovation) using a two-step process. Two authors (HH and NN) screened the titles and abstracts independently, and disagreements were discussed with a third author (MZ). After assessment of all titles and abstracts, the full text of any potentially relevant studies was retrieved and reviewed for inclusion.

### Data Extraction

We extracted data from each individual trial, including the study design, sample characteristics such as sample size, inclusion, and exclusion criteria. Characteristics of the intervention and control groups (if any), including the intervention modality, content, and duration, were also extracted. We identified the primary and secondary outcomes and extracted all outcome data.

### Assessment of Risk of Bias

We used the ROBINS-I (risk of bias [RoB] in nonrandomized studies of interventions) [21] and RoB [22] tools to assess RoB (Multimedia Appendix 2).

This study was based on data from published studies and did not require approval from an ethics committee.

### Data Synthesis

All characteristics and outcomes were reported as per the original paper—including type of study, type of intervention, and study outcomes. Outpatient adherence was defined as attendance at the hypertension clinic follow-up. Feasibility was defined as percentage of validated BP monitoring and measurements, feasibility of adjusting antihypertensives via telehealth, percentage of BP monitoring by teleconsultation, technology feasibility, proportion of women for whom BP was ascertained, resource utilization and identification and triage to appropriate follow-up, recruitment consent and retention, recruitment discontinuation, and adherence and persistence with self-monitoring, proportion of participants with satisfaction and adherence 80% or higher, and adherence to study protocol, as well as implied feasibility by the successful implementation of the eHealth intervention and completion of study. Participation rate was defined as the performance of a BP measurement with an eHealth intervention. Patient acceptance was reported as a qualitative outcome, which included ease of use assessed on a visual analog scale.

Mechanisms of action of the eHealth intervention were defined as “self-monitoring of BP,” “decision aid,”—either one or two-way communication between patient or their carer and clinician with conveyance of information allowing for decision-making regarding care and “education.” These mechanisms of action have previously been described in other systematic reviews looking at eHealth interventions for chronic disease [14].

Clinical outcomes were largely undefined—for example, stillbirth, neonatal death, and small for gestational age. Definitions of HDP (gestational hypertension, chronic hypertension, preeclampsia, de novo postpartum hypertension, superimposed preeclampsia, eclampsia, and hemolysis, elevated liver enzymes, low platelets syndrome) varied between studies—they either used their own study definition or used definitions for the conditions as per the National Institute of Health and Care Excellence definitions or the American College of Obstetricians and Gynecologists’ Committee on Practice Bulletins, International Society for the Study of Hypertension in Pregnancy. Risk factors for preeclampsia varied across the studies.

### Statistical Analysis

Meta-analyses of RCT findings were conducted where possible—Revman (The Cochrane Collaboration) was used to conduct the analysis and present the data. We used the Mantel-Haenszel method with fixed effect analysis for meta-analysis of outcomes. We summarized relative intervention effects for dichotomous outcomes as odds ratios (ORs)—these are summarized in forest plots.

We assessed the heterogeneity by visual inspection of the forest plot. Heterogeneity was then analyzed using a chi-square test on N‐1 degrees of freedom, with an α of 0.05 used for statistical significance and with the *I*^2^ test [23]. Other outcomes were reported in a narrative style with a summation of findings.

Due to the small number of studies, we were unable to assess for the existence of small study bias using funnel plots. There were insufficient extractable data to perform sensitivity analyses.

## Results

### Study Selection

**Figure 1.**
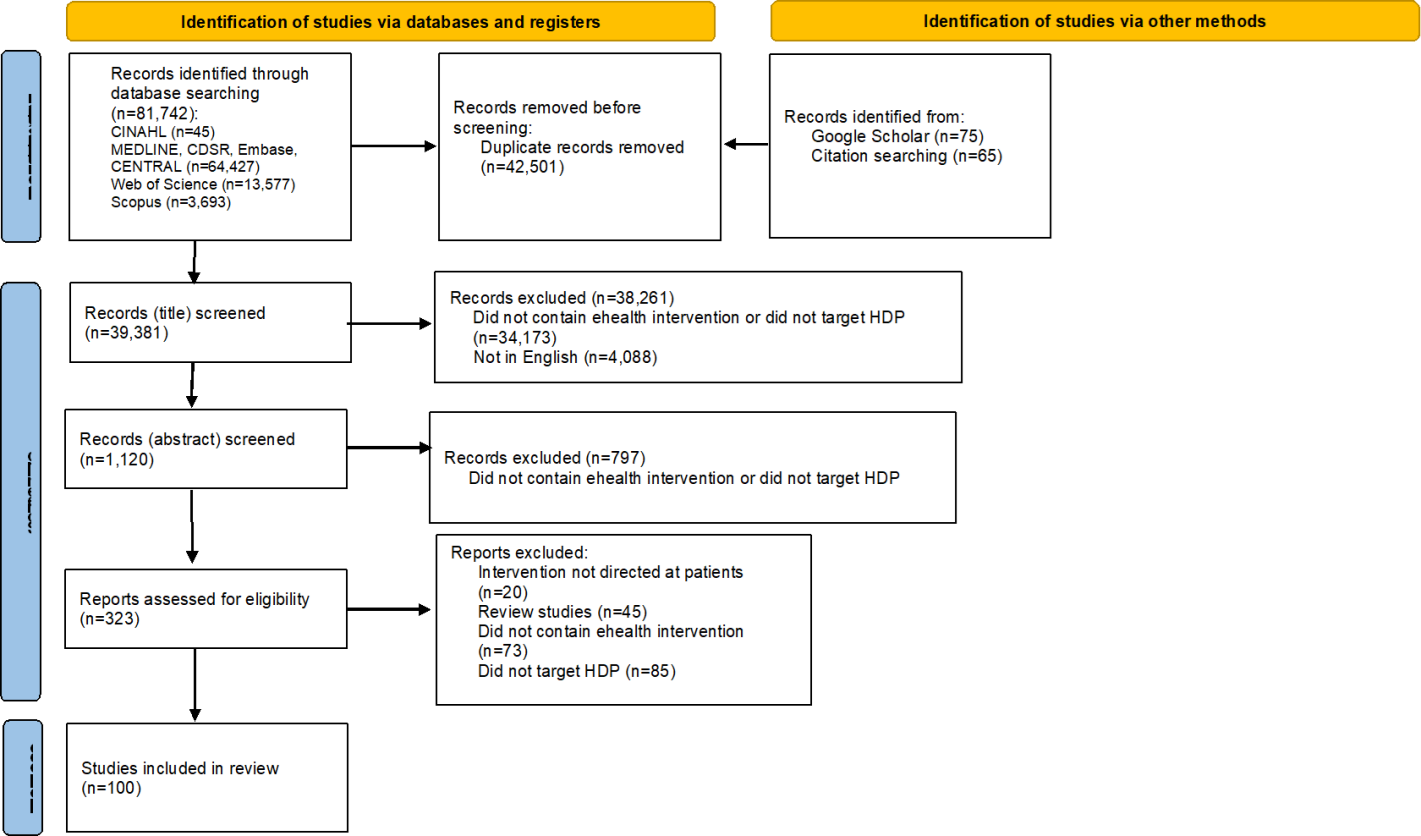
PRISMA diagram. HDP: hypertensive disorders of pregnancy; PRISMA: Preferred Reporting Items for Systematic Reviews and Meta‐Analyses.

We searched for studies published between database inception and August 24, 2024, and identified a total of 81,882 studies. After removing duplicates, we screened 39,381 study titles and further narrowed down our selection by reviewing 1120 abstracts. We screened 323 full-text publications based on inclusion and exclusion criteria. We ultimately included 96 studies that met our criteria with a total of 100 publications (4 publications included poster presentation of the study findings; Figure 1).

### Study Characteristics

Table 1 presents an overview of the key features of the studies included in this analysis. The included studies were conducted between 2004 and 2024, with the majority published after 2015. The majority of studies (n=58) were conducted in the United States. Of the 100 publications, there were 32 posters, 1 letter to the editor, 2 research letters, 1 brief communication, and 64 complete studies. Study types included case reports, case series, cohort studies, case control, and randomized controlled studies, as well as qualitative studies and cost analysis of previous studies.

**Table 1.** Overview of characteristics of studies^a^.

Characteristics	Studies (N=100), n (%)
Country	
United States of America	58 (58)
United Kingdom	18 (18)
Netherlands	4 (4)
Belgium	6 (6)
Ghana	2 (2)
Other^b^	12 (12)
Number of participants	
0‐50	21 (21)
51‐100	14 (14)
101‐200	21 (21)
201‐300	11 (11)
300+	29 (29)
Unclear	4 (4)
Length of intervention	
≤1 week	1 (1)
1‐3 months	7 (7)
4‐6 months	18 (18)
>6 months	54 (54)
Unclear	20 (20)
Type of study	
Cohort study	69 (69)
Case report or case series	4 (4)
Case control	9 (9)
Randomized controlled trial	11 (11)
Qualitative study	7 (7)
Type of intervention	
SMS text message	31 (31)
Bluetooth (BP^c^ cuff, digital scale)	19 (19)
Telehealth	25 (25)
Smartphone app	34 (34)
BP cuff with data transfer capability (Wi-Fi or internet)	12 (12)
Web-based platform	40 (40)
BP cuff with data transfer capability (cellular transmission)	4 (4)
Mobile phone (internet-based)	3 (3)
Tablet	4 (4)
Smartwatch	1 (1)
Automated tele device	1 (1)
Email	3 (3)
Artificial intelligence	1 (1)
Automated BP cuff	1 (1)
Video	2 (2)

a Total participants (n=61,539).

b Countries where n=1 (Hungary, France, India, Germany, Peru, Kenya, Japan, Mexico, Switzerland, Canada, Denmark, and Indonesia).

c BP: blood pressure.

Participant numbers ranged from 1 to 12,038, with the majority including more than 300 patients (n=29, 29%). Follow-up for the eHealth interventions was between 1 week and 12 months. Interventions were primarily targeted at self-monitoring of BP with reminders for BP checks, as well as transmission of BP and HDP symptom data, and two-way communication between patients and care providers. A total of 44 studies looked at antenatal patients, 46 looked at postnatal patients, and 10 studies looked at both groups.

In total, 18 studies [24-41] assessed a single outcome, while the rest looked at two or more outcomes.

Follow-up and application of the eHealth intervention ranged from 1 week to 1 year, with 20 studies not reporting on intervention length (Table 1).

eHealth interventions included SMS text message, Bluetooth, telehealth, smartphone app, Wi-Fi or internet-based or cellular transmission BP cuffs, web-based platform, mobile phone (internet-based), tablet, smartwatch, automated tele device, email, wearable device (smart wristband), and videos. Most studies involved eHealth interventions where more than one technology was used.

There were 60 studies that looked only at eHealth intervention with no comparator group, 36 studies compared an eHealth intervention to usual management (usually face-to-face), one study compared the implementation of the same eHealth intervention at different hospitals at different times [42], one study compared routine telehealth to mostly face-to-face review with some unofficial telehealth appointments [43], one study compared automated versus manual measurement of BP via remote BP monitoring [44], one study compared remote BP monitoring with communication via email compared to manual submission of data and usual care [45], and one study implemented the same eHealth intervention with clinicians aware (eHealth cohort) or blinded (control) to the remote BP measurements (Table 2) [46].

**Table 2.** Analysis of eHealth interventions.

eHealth interventions	Study
Modality of eHealth intervention	
SMS text message	[31-33,36,42,47-71]
Bluetooth (BP^a^ cuff, digital scale)	[15,25,27,30,35,45,56,69,72-86]
Telehealth	[30,33,34,36,37,39,40,43,64,68,70-72,74-76,79,80,82,85-101]
Smartphone app	[15,27,28,31,35,37,45,48,51,56,63,68,69,73,77,78,81,84,85,93,94,98,102-116]
BP cuff with data transfer capability (Wi-Fi or internet)	[25,28,29,38,44,82,83,91,95,108,117-119]
Web-based platform	[15,25,27,28,30,31,33,35-37,39,42,44,46,50,54-58,60-64,66,69-71,75,77-79,81-84,86,89,94,95,98,99,103,104,110,111,120]
BP cuff with data transfer capability (cellular transmission)	[39,71,89,97,120]
Mobile phone (internet-based)	[24,26,32]
Tablet	[74-76,80,86]
Smartwatch	[27]
Automated tele device	[92]
Email	[15,45,68,121]
Wearable device (smart wristband)	[78]
Artificial intelligence	[45]
Automated BP cuff (unclear mode of transmission of data)	[41,122]
Video	[41,116]
Mechanism of effect of eHealth intervention	
Self-monitoring of BP	[15,24-33,35-39,41,42,44-51,53-64,66-98,101-104,106-114,117-122]
Decision aid	[15,24,25,27,28,30,32-35,37-43,45-62,64-77,79-91,93-105,107,109,111-115,117,119-122]
Education	[116]
Types of patients	
At risk of HDP^b^	[15,26,35,41,44,56,58,66,69,73,77,81,86-88,98-100,105,106,108,110,111,113,114,116,122]
HDP	[24,25,27-34,36-39,42,43,45-55,57,59-65,67-72,74-76,78-80,82-85,89-97,101-104,107,109,112,115,117-121]
Comparator group	
Nil	[27-29,32,36,38-41,47,50,53,55,56,58,59,62,64-67,71,73,74,76-78,81,82,85-89,94,99,102,103,105,106,108,110-112,115,117,119-122]
Historical data	[15,33,35,49,54,57,61,68,90,92,96,97,100]
Usual care (no eHealth intervention)	[24-26,30,31,34,37,42,43,48,51,52,60,61,63,69,70,75,79-81,83,84,91,93,95,96,98,101,104,107,109,113,114,116,118]
Same eHealth intervention (implementation of same eHealth intervention at different institution)	[42]
Same eHealth intervention—clinician aware (eHealth cohort) or blinded to remote BP results (control)	[46]
Remote BP monitoring with manual submission of data and usual face-to-face care	[45]
Remote BP monitoring with manual reporting of BP (compared to automatic transmission)	[45]
Type of study	
Cohort study	[25,27-30,33,34,36,38-40,42-45,47,49,52-59,62,64-72,74,76,78,79,81,83,85-94,97,99,100,102,103,105-110,112,115,116,118-121]
Case report or case series	[73,111,117]
Case control	[15,26,32,35,113,114]
Randomized or nonrandomized controlled trial	[24,37,46,48,51,60,61,63,75,79,84,98,101,104]
Qualitative study	[31,41,77,80,82,122]
Outcome of eHealth intervention	
Readmission rate	[33,45,47,49-51,57,59,60,62,65,67,68,70,75,84,88,90,92-94,103,109,119]
Outpatient adherence rate, that is, follow-up rate	[34,40,49,52,57,63,70,72,79,90,97,100,113,118,119]
Qualitative assessment of participant perception of intervention	[30,31,39,45,50,58,67,77,84,85,87,101,104,112,121]
Qualitative assessment of participants’ support person perception of intervention (interviews)	[122]
BP	[24,63,66,70,71,75,84,88,90,102,104,106,110]
Patient satisfaction	[15,27,28,48,54-56,62,68,75,77-82,85,89,92-94,102,108,110,112,115]
Feasibility	[46,52,53,55-57,62,63,66,81,86,88,89,92-94,103,108]
Number of face-to-face visits (aimed to reduce) or health care service utilization	[15,55,65,70,75,79,83,92,95,96,100,105,113,114]
Participation rate (ie, BP measurement)	[15,29,32,36,40,42,44,45,48,50,59-64,66-71,75,77,81,86,87,94,97,99,101,103,108,110,120,121]
Medication titration or commencement	[42,45,50,53,59,60,62,63,67,70,75,76,94,111,117]
Antenatal admission rate	[15,83,91,95,114]
Recruitment or consent rate	[63,64,66,74,80,82,88,97,114]
Clinical outcomes	[15,26,33,38,42,43,45,46,53,54,62,63,66,73,76,78,81,83,84,86,88,91,94,96-99,103-107,109-111,113,114,117-119]
Patient acceptance (ease of use)	[46,63,101,115]
Cost analysis	[25,35,37,46,54,96,113]
Patient knowledge	[41,85,116]
Timing in pregnancy	
Antenatal	[15,25,27,31,35,39-41,43,44,46,55,56,63,66,69,73,77,78,81-83,85,87,89,91,95,98,99,102,104,106-108,110,112-117,120,122]
Postnatal	[24,26,28,30,32-34,36,38,42,45,47-53,57,59-62,64,65,67,70-72,74-76,79,80,84,88,90,92-94,96,97,100,101,103,105,109,119]
Antenatal and postnatal	[29,37,54,58,68,69,86,111,118,121]

a BP: blood pressure.

b HDP: hypertensive disorders of pregnancy.

Most interventions were based around self-monitoring of BP, with eHealth being used to transmit or store BP and send and receive reminders, as well as communicate with health care providers regarding BP, symptoms, and management.

**Figure 2.**
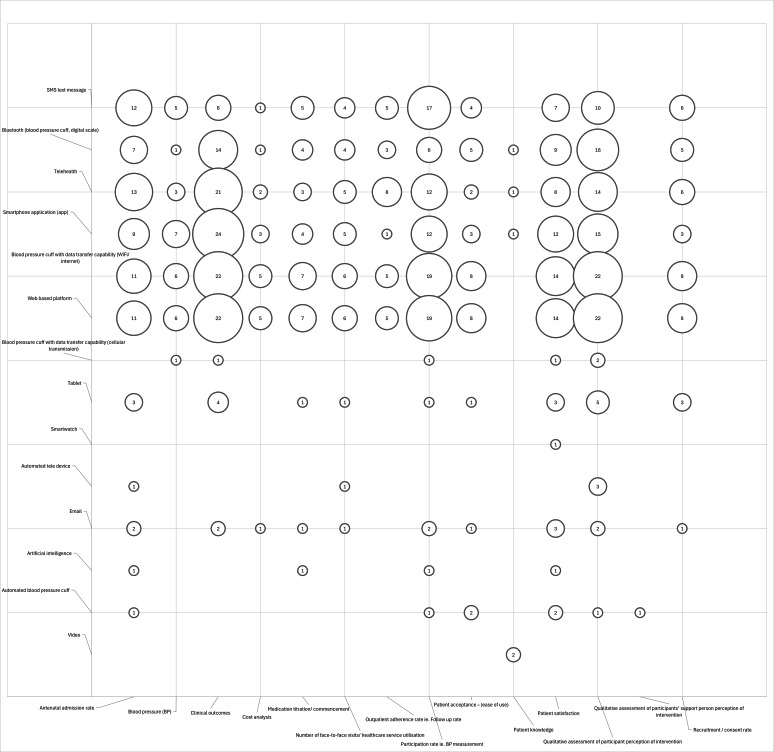
Bubble plot of reported outcomes by type of eHealth intervention.

The outcomes targeted by the eHealth interventions and reported on in the studies included admission rate, outpatient adherence rate, that is, follow-up rate, qualitative patient perception of intervention (interviews), actual BP, patient satisfaction, feasibility, degree of health care service utilization, participation rate, that is, BP measurement, medication titration or commencement, recruitment or consent rate, clinical outcomes, patient acceptance (ease of use), patient knowledge and motivation, and cost analysis (Table 2). Figure 2 depicts a bubble plot of reported outcomes.

Often, the outcomes were not defined and only reported on. Readmission rate was defined differently in included studies—readmission within 7 days, 14 days, 30 days, or 6 weeks, or not defined at all.

The reported outcomes included in these studies were heterogeneous and largely lacking in detail.

### RoB of Included Studies

A total of 29 studies were unable to be assessed due to insufficient information, 9 studies were assessed as low RoB, 13 studies were assessed as moderate RoB, and 30 studies were assessed as serious RoB (Multimedia Appendix 2).

### Synthesis of Results

Outcomes have been categorized into three main categories—“clinical outcomes,” “qualitative outcomes,” and “resource utilization.”

### Clinical Outcomes

The majority of reported clinical outcomes were nonspecific and discussed in broad terms of maternal or neonatal or infant morbidity without the specific condition. Most studies found similar maternal and neonatal clinical outcomes [26,46,63,83,95,96,104,107,113,114]. Three case reports described the management of patients with HDP using eHealth [73,111,117]. eHealth interventions reduced the incidence of small for gestational age and preterm birth in one study [26], but increased it in another study [98].

### Medication Adjustment

Remote BP monitoring via eHealth was used to commence [50,62,70] or titrate patients’ antihypertensive [53,63,94] or both [42,60,74,111,117].

When comparing remote BP monitoring with automatic transmission of data compared to manual submission, there was no difference in initiation or titration of antihypertensives [45].

### Blood Pressure

In total, 4 studies found that participants using eHealth methods to either monitor or diagnose HDP had lower BPs [24,51,84]—statistically significant; 2 studies found that BP was higher with eHealth BP monitoring [63,90]—not statistically significant; and 1 study found no difference in BP between eHealth and usual care [104]—not statistically significant.

### Admission Rate

#### Antenatal Admission Rate

In total, 5 studies reported on antenatal admission rate as an outcome; 4 studies found reduced antenatal admissions [15,83,91,95] comparing eHealth to usual care, while 1 study found no difference [114].

#### Readmission Rate

Most readmissions were secondary to hypertension or were not specified. A total of 9 studies reported on readmission rate with no comparator [47,50,53,59,61,62,74,75,88,92-94].

In total, 6 studies found that readmission rates were lower with eHealth interventions compared to usual care [33,49,60,70,84,96], 1 study showed no difference [90], and 2 studies showed an increase in readmission rates with eHealth intervention [68,109].

When comparing remote BP monitoring with automatic transmission of data compared to manual submission, there was no difference in hypertension-related emergency department presentation or hospital readmission [45].

### Outpatient Adherence

eHealth interventions improved outpatient adherence (outpatient clinic follow-up)—increased rate of follow-up, as well as decreased time to follow up—compared to conventional face-to-face care. Telehealth also increased postpartum depression screening, Papanicolaou test completion, and long-acting reversible contraception initiation [100].

Only 1 study [52] showed no difference in postnatal outpatient follow-up rates between remote BP monitoring with Bluetooth BP cuff and database storage of data and nonusers.

#### Participation Rate (BP Ascertainment)

eHealth interventions increased the rate of BP ascertainment (reported as participation rate) compared to usual care. One study reported a shortened interval between BP measurements during the prenatal and postpartum periods for all patients [69]. eHealth interventions increased adherence to hypertension-specific guidelines [70]. Remote BP monitoring via eHealth reduced racial disparities in adhering to postpartum BP checks [71].

One RCT reported no significant differences in the rate of clinically documented BP monitoring at 7‐10 days postpartum between remote BP monitoring compared to routine office-based care [101].

#### Incidence of HDP

A total of 7 studies reported on the incidence of HDP without a comparator [66,74,88,97,99,108,110]. Two studies reported outcomes compared to historical data—one study found fewer cases of preeclampsia in the eHealth cohort compared to control [114], while one study found the converse—increased diagnosis of HDP in the eHealth cohort [15]. A total of 12 studies compared an eHealth intervention to conventional care—4 studies found eHealth reduced HDP incidence [96,98,114,118], 3 studies found eHealth increased incidence of HDP [15,26,63], 4 studies found higher incidence of gestational hypertension but lower incidence of preeclampsia [83,91,107,118], and 1 study found no difference [43].

### Qualitative Outcomes

#### Recruitment or Consent

The recruitment or consent rate for using eHealth ranged from 40.38% to 98%. The consent rate for completion of patient experience questionnaires ranged from 41% to 100%.

The recruitment rate to use the eHealth intervention, as well as to complete a feedback survey, was similar between patients at risk of HDP and those with HDP.

#### Feasibility

Feasibility was assessed as an outcome in 18 studies. Specific outcomes were described in 2 studies—the feasibility of BP monitoring in postpartum women by teleconsultation was 95.23% [53]. Feasibility was demonstrated with a proportion of 0.767 participants (*P*=.003; 95% CI 0.577-0.885). A total of 30 participants with feasibility measured using a 1-sided *t* test of the proportion of participants with satisfaction (via validated Likert-style postparticipation surveys) and adherence of 80% or higher (recorded BPs or expected BPs) [94].

#### Patient Satisfaction, Qualitative Assessment of Patient Experience

All studies have shown that participants using eHealth interventions were satisfied and found it easy to use. The eHealth interventions were acceptable to all patients. Almost all patients would elect to use the eHealth intervention again in the future.

Support people of the pregnant person were reported to have a positive perception of home BP monitoring [122].

#### Ease of Use

Most users of eHealth interventions found it to be easy to use and had benefits over usual care. It required very little effort to learn how to use the intervention [31,39,50,59,67,79,81,82,85,86,103,121].

One study reported patients encountering some difficulty in learning how to use the new technology [80].

#### Privacy Concerns

Three studies reported on the users’ privacy concerns—most patients did not have privacy concerns regarding the technology in the eHealth intervention [57,80], only one of the studies reported 3 women expressing concerns regarding sharing health data as a threat to their privacy [82].

#### Recommendation to Others

A total of 9 studies reported on users’ experience and whether they would recommend the eHealth intervention to users—most users of the eHealth intervention would recommend it to others [15,55,57,74,77,80,81,85,86].

#### Preference for eHealth Over Conventional Models of Care

In total, 3 studies reported on patients preferring eHealth over conventional models of care [56,77,80], and 3 [31,80] studies reported few patients preferred conventional models of care over eHealth—in 1 study, this proportion was 28% [85].

#### Benefits of eHealth

Benefits of eHealth management of HDP include reduction of anxiety [58], significant decrease in perceived stress [39], good communication (n=39, 30%) [30,54], increased awareness of hypertension, preeclampsia and symptoms with earlier treatment [59,67,85,116,122], feeling of safety [82,93], convenience, perceived better care, patient empowerment [39], and sense of empowerment [78].

Users felt it was “very easy” or “somewhat easy” to fit an eHealth intervention (remote BP monitoring) into their lifestyle [85].

#### Challenges and Concerns

Some participants reported concerns with the device itself [89], such as problems with wearing the device or perceiving that it gave higher readings than clinical BP monitors. Some users of the eHealth intervention reported increased anxiety and concerns with the remote monitoring process [30,89,121]. Others reported that performing self BP monitoring can be bothersome [31,77] and that they received excessive calls from the call center [39].

#### Patient Knowledge and Motivation

One study examined an animation video on preeclampsia to educate and motivate pregnant women—they found this intervention to have increased the patients’ knowledge and motivation for preeclampsia prevention [116]. Patients who received education had a better understanding of their pregnancy and preeclampsia [31,41,67,85,108].

### Resource utilization

#### Cost Analysis

Studies that included cost analysis and comparison between eHealth and usual care found that eHealth interventions were either cost-neutral [46] or resulted in a cost saving to the health system [25,35,54,113].

One study reported slightly increased costs associated with the eHealth intervention compared to usual care; however, this was not statistically significant [37].

#### Health Care Service Utilization

eHealth interventions largely reduced health care service utilization. A total of 6 studies found that the eHealth intervention reduced the number of outpatient appointments, number of days admitted, admissions, and patient travel [15,46,55,92,95,107,114].

Two studies found equivocal health care utilization between eHealth intervention and usual care [75,113].

Two studies found that the eHealth intervention increased hospital and specialist follow-up [79,96].

Two studies studying postnatal patients reported on health care service utilization with no comparator [57,105].

##### RCT Results

**Figure 3.**
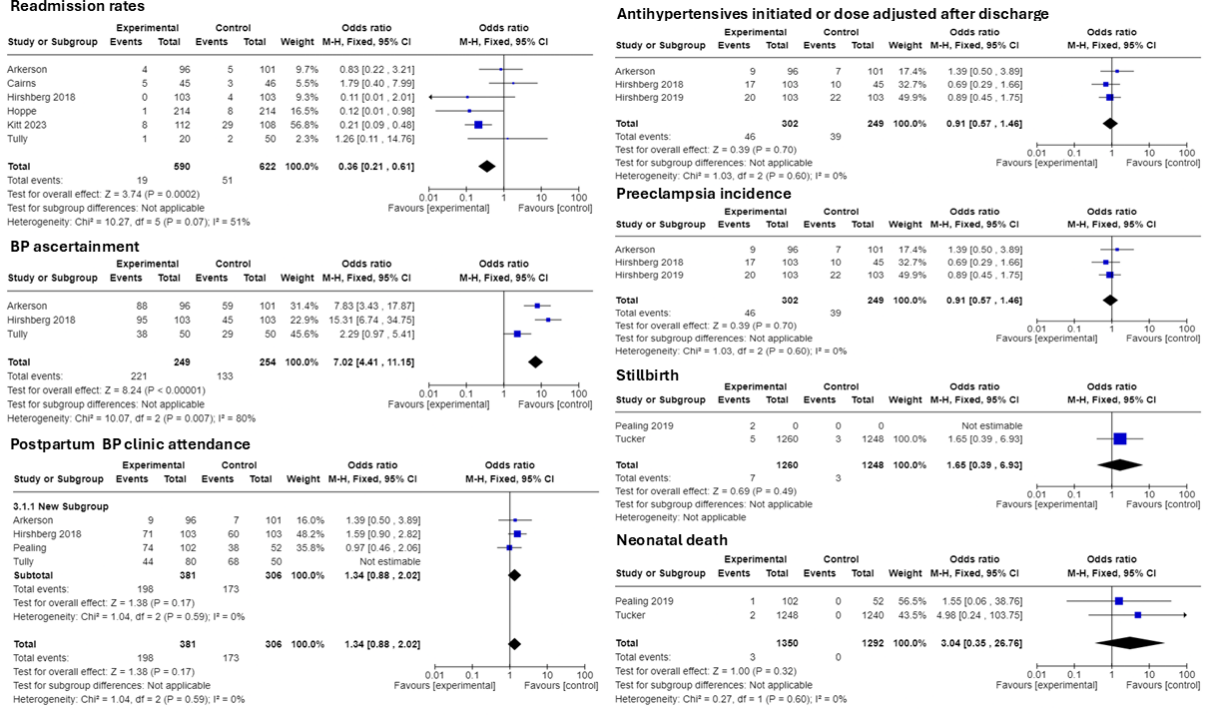
RCT results. *I*^2^ values varied between 0% and 80%, indicating moderate, substantial, or considerable heterogeneity within the studies. BP: blood pressure; RCT: randomized controlled trial [48,51,60,61,63,75,84,98,101].

There were 11 RCTs [24,37,46,48,51,60,61,63,84,98,101,104] comparing eHealth and usual care (control), which reported on a variety of outcomes. eHealth interventions reduced readmission rates (OR 0.4, 95% CI 0.23-0.71), improved the likelihood of BP ascertainment (OR 7.02, 95% CI 4.41-11.15), and improved attendance at postpartum hypertension clinic (OR 1.44, 95% CI 0.98-2.12; Figure 3).

eHealth intervention participants were slightly less likely to have antihypertensives and were initiated or dose adjusted after discharge (OR 0.91, 95% CI 0.57-1.46). eHealth interventions increased the diagnosis of preeclampsia (OR 1.14, 95% CI 0.81-1.62).

eHealth intervention participants were more likely to experience stillbirth rates compared to control (OR 0.55, 95% CI 0.15-1.99), favoring control, and neonatal death (OR 0.33, 95% CI 0.04-2.89), favoring control.

## Discussion

### Principal Results

We conducted this systematic review to identify all eHealth interventions targeting patients at risk of or with HDP and report on their feasibility and acceptability for implementation, as well as clinical outcomes and resource utilization. In observational studies, there were no significant differences in the observed clinical outcomes. Within qualitative outcomes, eHealth interventions appeared to be feasible, and all studies showed that participants were satisfied and found eHealth interventions easy to use. There was equivocal evidence regarding the cost benefits of eHealth interventions, but it did demonstrate largely reduced health care service utilization. Meta-analysis of RCT data showed that eHealth interventions reduced readmission rates (OR 0.4, 95% CI 0.23-0.71), improved the likelihood of BP ascertainment (OR 7.02, 95% CI 4.41-11.15), and improved attendance at postpartum hypertension clinic (OR 1.44, 95% CI 0.98-2.12). The implications of these findings indicate that eHealth interventions have great potential in managing HDP with little apparent risk to patients.

We identified 96 studies (100 publications) with 61,539 participants, involving a variety of different eHealth technologies to replace or augment standard care to either manage or prevent the development of HDP.

Due to the considerable heterogeneity between study designs, comparators, diagnostic criteria of HDP used, included participants, reported outcomes, and eHealth interventions, we were only able to perform meta-analyses for a few outcomes.

eHealth mobile and web apps can be used to educate, prevent, manage, and follow up patients with HDP. Modifiable risk factors for preeclampsia include chronic hypertension, obesity, high gestational weight gain, and sedentary lifestyle [123-127]. Effective strategies for the early diagnosis and management of preeclampsia include self-monitoring of BP, magnesium sulfate therapy, and timely delivery [15,128]. Lifestyle modifications can mitigate the development of and improve the management of preeclampsia [129].

Most studies were cohort studies—some studies were classified as case-control studies by the authors; however, they involved comparison of two cohorts over a period of time. Most studies used the eHealth intervention as either a unidirectional or bidirectional decision aid to aid decision-making for either the clinician or patient regarding management and intervention.

The majority of eHealth interventions contained a web-based platform component, usually for transmission and storage of data. Clinical outcomes were the most frequently reported; however, these were a mixture of maternal and fetal outcomes, often with no sensitivity analysis performed. Definitions of these outcomes varied between studies and were often not defined. Additionally, there were a large number of other outcomes reported, which limited our ability to synthesize the data and formulate conclusions. Due to the high variability in study design, comparators, definitions, and outcomes, a grouped analysis and heterogeneity report of subgroups was unable to be performed.

Considering all eHealth interventions for HDP published thus far, these interventions appear to be acceptable to patients, with few patient concerns, and were feasible to implement. Clinical outcomes and BP varied between those who used eHealth interventions and those who did not. The impacts of these interventions include reduced antenatal admission rates, as well as readmission rates, improved outpatient follow-up of hypertension, improved BP measurement and adherence to postpartum hypertension management guidelines, improved patient knowledge of preeclampsia, and reduced health care utilization with associated cost savings. eHealth interventions were used successfully to commence or titrate antihypertensives. Meta-analyses of RCTs showed a statistically significant reduction in readmission rates and an increased likelihood of BP ascertainment and improved attendance at postpartum hypertension clinic.

### Limitations

The quality of the evidence was low and inadequate due to the small, heterogeneous study populations and variability of eHealth technologies and reported outcomes.

Subgroup analysis was unable to be performed on the majority of outcomes due to the considerable heterogeneity of reported data points (MultimediaAppendices 3  4 [15,24-123]). The definition of outcome of interest varied greatly—for example, the time period of “Readmission rate” varied between not reported to 6 months (with a range of not reported, 10 d, 2 wk, 16 d, 6 wk, 8 wk, and 6 mo), the comparators ranged from “no comparator” to “conventional care,” “historical cohort,” or other definitions. The readmission rate was largely reported in numbers; however, some studies reported qualitative descriptions of readmission rates, such as “equally likely to have a hypertension-related hospital readmission” [45] and “1 less readmission for every 100 patients engaged in the program” [70]. In cases of pooled analysis of RCT results, the different definitions of outcomes of interest reduce the validity of pooled results (readmission time period ranged between 10 d and 6 mo, and the definition of preeclampsia was reported in one study [63], but not the other [101]). For the other outcomes with grouped analysis—BP ascertainment, postpartum BP clinic attendance, antihypertensives initiated or dose adjusted after discharge, stillbirth, and neonatal death, the reliability of the results is limited by the small number of included participants and studies.

The overall small sample size limited our ability to conduct robust statistical estimates of heterogeneity and limited the reliability of the *I*^2^ estimate. The CIs for *I*^2^ in the RCT grouped analysis were substantial, indicating uncertainty about the true level of heterogeneity, further limiting the reliability of our reported results.

The majority of studies were assessed to be at uncertain or high RoB. The large number of studies with uncertain RoB assessment was due to poor methodological and outcome reporting of studies. There is likely to be confounding and systematic errors due to the inclusion of a large number of studies assessed as having an uncertain or high RoB in this systematic review, which can significantly impact the reliability and validity of our conclusions and reduce the overall quality of evidence. As only 9 studies were assessed as low RoB, our ability to synthesize and interpret results or derive reliable conclusions and recommendations from this data is limited; the effects we reported on are uncertain due to the low quality of data and heterogeneity of trial design.

The majority of studies were conducted in Western, high-income countries—their findings may not be generalizable to all populations.

The strength of this review is the comprehensive inclusion and analysis of all eHealth interventions targeted at HDP.

### Comparison With Prior Work

Other systematic reviews have assessed the impact of eHealth interventions in obstetrics and in patients at risk of or with HDP. These reviews had a narrower scope and restricted their inclusion criteria, focusing on specific intervention types (home BP monitoring [130,131], telehealth [132]), or were limited to specific patients (postpartum patients [133]). Our review is unique in that this is the most comprehensive review on all modalities of eHealth technology applied to all patients at risk of or with HDP, with no other exclusion criteria restricting study inclusion. Again, they were unable to make concrete recommendations given the small, heterogeneous study population and reported outcomes. Similar to our review, they have also found that telehealth interventions overall improved obstetric outcomes, decreased the need for obstetric monitoring office visits while maintaining maternal and fetal outcomes [10,134,135].

### Conclusions

The data suggest that the current evidence for the use of eHealth interventions targeted at patients at risk of or with HDP is of low quality and insufficient to make a recommendation regarding their routine use in clinical care.

Our findings indicate that there is poor quality and low-level evidence that eHealth interventions are feasible, safe, and acceptable to patients, and there is very limited evidence that it may reduce health care utilization, improve follow-up and BP ascertainment, and reduce admissions, as well as confer some economic benefit compared to usual care with a generally positive patient experience with minimal patient concerns.

The wide acceptance of these eHealth interventions from the 61,539 patients included in the studies within the review indicates that future application of eHealth technologies is likely to be accepted. The most frequently used technologies (mobile phone and internet-based) are relatively accessible for most patients from different backgrounds, and prospective studies aiming to implement these interventions in any setting should be feasible.

The broad range of use for the eHealth interventions in HDP shows promise in their applicability to other aspects of antenatal and obstetric care. There is insufficient evidence to draw any conclusions on the clinical efficacy of these interventions, and future research is required to further explore this. We have shown that eHealth interventions can be successfully implemented into obstetric care, and prospective integration of these interventions into routine care should be considered.

## Supplementary material

10.2196/77064 Multimedia Appendix 1 Search strategy.

10.2196/77064 Multimedia Appendix 2 Risk of bias assessment.

10.2196/77064 Multimedia Appendix 3 Results summary.

10.2196/77064 Multimedia Appendix 4 Study summary.

10.2196/77064 Checklist 1 PRISMA checklist.

## Abbreviations

BP: blood pressure
HDP: hypertensive disorders of pregnancy
OR: odds ratio
PRISMA: Preferred Reporting Items for Systematic Reviews and Meta‐Analyses
RCT: randomized controlled trial
RoB: risk of bias
ROBINS-I: risk of bias in nonrandomized studies of interventions
