# Supplementary material for: eHealth and Hypertensive Disorders of Pregnancy: Systematic Review
Source: J Med Internet Res. 2025 Sep 10;27:e77064. doi: 10.2196/77064 (PMC12422594; doi:10.2196/77064)
Supplement: Multimedia Appendix 1 [file jmir-v27-e77064-s001.docx]

Appendix 1 – Search Strategy

Embase Classic+Embase <1947 to 2023 November 17>

Ovid MEDLINE(R) ALL <1946 to November 17, 2023>

EBM Reviews - Cochrane Central Register of Controlled Trials <October 2023>

EBM Reviews - Cochrane Database of Systematic Reviews <2005 to November 15, 2023>

1 Telemedicine/ 87498

2 Telemedicine*.mp. 111858

3 (e-health* or ehealth*).mp. 26517

4 Telehealth.mp. 46239

5 (mobile health or m-health or health).mp. 9386128

6 (digital health or online or telecommunication* or teleconferenc* or tele-conferenc* or apps or artificial intelligence or AI or digital or ehealth or e-health or electronic health record* or emedicine or e-medicine or etherap* or e-therap* or health technolog* or ICT or instant messag* or information technolog* or internet or web or mhealth or m-health or MMS or mobile* or podcast* or smartphone* or smart phone* or SMS or social media or technolog* or telecare or tele-care or telehealth or tele-health or telemedicine or tele-medicine or telerehabilitation or tele-rehabilitation or virtual or web-based or website* or virtual reality).tw. 3742111

7 (android or app* or audio or blog or CBT or CD-ROM or cell phone* or cellphone* or chat).tw. 19338141

8 (computer* or cyber* or DVD or eHealth or e-health or "electronic health*" or e-Portal or ePortal).tw. 949572

9 (eTherap* or forum* or gaming or information technolog* or instant messag* or internet* or ipad or iphone or ipod or web* or WWW).tw. 831572

10 (smart phone* or smartphone* or social network* site* or mobile phone* or email* or mobile or multimedia or online* or personal digital assistant or PDA or SMS or social medi* or software or telecomm* or telehealth* or telemed* or telemonitor* or telephone or telepsych* or teletherap* or text messag* or texting or podcast or virtual*).tw. 2300381

11 1 or 2 or 3 or 4 or 5 or 6 or 7 or 8 or 9 or 10 28421523

12 hypertension, pregnancy-induced/ or eclampsia/ or hellp syndrome/ or pre-eclampsia/ 109995

13 (pre-eclamp* or preeclamp* or eclampsia* or Hellp syndrome*).mp. 157133

14 ((Pregnan* or maternal* or gestational*) adj3 (hypertension* or high blood pressure*)).mp. 56436

15 12 or 13 or 14 184160

16 11 and 15 58440

17 limit 16 to english language 54715

18 limit 17 to female 43160

19 limit 18 to human 41768

Search rerun on 260824 – 536 extra papers found (after duplications removed)

**Scopus -searched 22/11/23**

**3,157 documents found**

( ( TITLE-ABS-KEY ( ( pregnan*  OR  maternal*  OR  gestational* )  W/3  ( hypertension*  OR  "high blood pressure*" ) ) )  OR  ( TITLE-ABS-KEY ( pre-eclamp*  OR  preeclamp*  OR  eclampsia* ) ) )  AND  ( TITLE-ABS-KEY ( telemedicine*  OR  telehealth*  OR  "digital health"  OR  online  OR  telecommunication*  OR  teleconferenc*  OR  tele-conferenc*  OR  apps  OR  digital  OR  ehealth  OR  e-health  OR  "electronic health record*"  OR  emedicine  OR  e-medicine  OR  etherap*  OR  e-therap*  OR  "health technolog*"  OR  ict  OR  "instant messag*"  OR  "information technolog*"  OR  internet  OR  web  OR  mhealth  OR  m-health  OR  mms  OR  mobile*  OR  podcast*  OR  smartphone*  OR  "smart phone*"  OR  sms  OR  "social media"  OR  technolog*  OR  telecare  OR  tele-care  OR  telehealth  OR  tele-health  OR  telemedicine  OR  tele-medicine  OR  telerehabilitation*  OR  tele-rehabilitation*  OR  virtual  OR  web-based*  OR  website*  OR  "virtual reality"  OR  "mobile health"  OR  m-health  OR  health  OR  android  OR  app*  OR  audio  OR  artificial intelligence   OR  AI   OR  blog  OR  cbt  OR  cd-rom  OR  "cell phone*"  OR  cellphone  OR  chat  OR  computer*  OR  cyber*  OR  dvd  OR  ehealth  OR  e-health  OR  "electronic health*"  OR  e-portal  OR  eportal  OR  etherap*  OR  forum*  OR  gaming  OR  "information technolog*"  OR  "instant messag*"  OR  internet*  OR  ipad  OR  iphone  OR  ipod  OR  web*  OR  www  OR  "smart phone*"  OR  smartphone  OR  "social network* site*"  OR  "mobile phone*"  OR  email*  OR  mobile  OR  multimedia  OR  online*  OR  "personal digital assistant*"  OR  pda  OR  sms  OR  "social medi*"  OR  software  OR  telecomm*  OR  telehealth*  OR  telemed*  OR  telemonitor*  OR  telephone  OR  telepsych*  OR  teletherap*  OR  "text messag*"  OR  texting  OR  podcast  OR  virtual* ) )

**Cinahl**

| 4:24:14 AM | | | |  |  |
| --- | --- | --- | --- | --- | --- |
| S8 | S4 AND S5 | Expanders - Apply equivalent subjects Narrow by Language: - english Narrow by SubjectGender: - female Search modes - Boolean/Phrase | Interface - EBSCOhost Research Databases Search Screen - Advanced Search Database - CINAHL Complete | | 4,767 |
| S7 | S4 AND S5 | Expanders - Apply equivalent subjects Narrow by SubjectGender: - female Search modes - Boolean/Phrase | Interface - EBSCOhost Research Databases Search Screen - Advanced Search Database - CINAHL Complete | | 4,856 |
| S6 | S4 AND S5 | Expanders - Apply equivalent subjects Search modes - Boolean/Phrase | Interface - EBSCOhost Research Databases Search Screen - Advanced Search Database - CINAHL Complete | | 5,809 |

| **#** | **Query** | **Limiters/Expanders** | **Last Run Via** | **Results** |
| --- | --- | --- | --- | --- |
| S6 | S4 AND S5 | Expanders - Apply equivalent subjects Search modes - Boolean/Phrase | Interface - EBSCOhost Research Databases Search Screen - Advanced Search Database - CINAHL Complete | Display |
| S5 | (MH "Pregnancy-Induced Hypertension+") OR ( (MH "Pre-Eclampsia+") OR (MH "Eclampsia+") OR (MH "HELLP Syndrome") ) OR ( pre-eclamp* or preeclamp* or eclampsia* ) OR ( (Pregnan* or maternal* or gestational*) N3 (hypertension* or high blood pressure*)) ) | Expanders - Apply equivalent subjects Search modes - Boolean/Phrase | Interface - EBSCOhost Research Databases Search Screen - Advanced Search Database - CINAHL Complete | Display |
| S4 | ( (MH "Telemedicine+") OR "Telemedicine*" ) OR ( "Telehealth*" OR (MH "Telehealth+") ) OR ( TI ( "digital health" or online or telecommunication* or teleconferenc* or tele-conferenc* or apps or digital or ehealth or e-health or "electronic health record*" or emedicine or e-medicine or etherap* or e-therap* or "health technolog*" or ICT or "instant messag*" or "information technolog*" or internet or web or mhealth or m-health or MMS or mobile* or podcast* or smartphone* or "smart phone*" or SMS or "social media" or technolog* or telecare or tele-care or telehealth or tele-health or telemedicine or tele-medicine or telerehabilitation* or tele-rehabilitation* or virtual or web-based* or website* or "virtual reality" or "mobile health" or m-health or health ) OR AB ( "digital health" or online or telecommunication* or teleconferenc* or tele-conferenc* or apps or digital or ehealth or e-health or "electronic health record*" or emedicine or e-medicine or etherap* or e-therap* or "health technolog*" or ICT or "instant messag*" or "information technolog*" or internet or web or mhealth or m-health or MMS or mobile* or podcast* or smartphone* or "smart phone*" or SMS or "social media" or technolog* or telecare or tele-care or telehealth or tele-health or telemedicine or tele-medicine or telerehabilitation* or tele-rehabilitation* or virtual or web-based* or website* or "virtual reality" or "mobile health" or m-health or health ) ) OR ( TI ( android or app* artificial intelligence or AI or audio or blog or CBT or CD-ROM or "cell phone*" or cellphone or chat or computer* or cyber* or DVD or eHealth or e-health or "electronic health*" or e-Portal or ePortal or eTherap* or forum* or gaming or "information technolog*" or "instant messag*" or internet* or ipad or iphone or ipod or web* or WWW ) OR AB ( android or app* or audio or blog or CBT or CD-ROM or "cell phone*" or cellphone or chat or computer* or cyber* or DVD or eHealth or e-health or "electronic health*" or e-Portal or ePortal or eTherap* or forum* or gaming or "information technolog*" or "instant messag*" or internet* or ipad or iphone or ipod or web* or WWW ) ) OR ( TI ( "smart phone*" or smartphone or "social network* site*" or "mobile phone*" or email* or mobile or multimedia or online* or "personal digital assistant*" or PDA or SMS or "social medi*" or software or telecomm* or telehealth* or telemed* or telemonitor* or telephone or telepsych* or teletherap* or "text messag*" or texting or podcast or virtual* ) OR AB ( "smart phone*" or smartphone or "social network* site*" or "mobile phone*" or email* or mobile or multimedia or online* or "personal digital assistant*" or PDA or SMS or "social medi*" or software or telecomm* or telehealth* or telemed* or telemonitor* or telephone or telepsych* or teletherap* or "text messag*" or texting or podcast or virtual* ) ) | Expanders - Apply equivalent subjects Search modes - Boolean/Phrase | Interface - EBSCOhost Research Databases Search Screen - Advanced Search Database - CINAHL Complete | Display |
| S3 | S1 AND S2 | Expanders - Apply equivalent subjects Search modes - Boolean/Phrase | Interface - EBSCOhost Research Databases Search Screen - Advanced Search Database - CINAHL Complete | 5,809 |
| S2 | (MH "Pregnancy-Induced Hypertension+") OR ( (MH "Pre-Eclampsia+") OR (MH "Eclampsia+") OR (MH "HELLP Syndrome") ) OR ( pre-eclamp* or preeclamp* or eclampsia* ) OR ( (Pregnan* or maternal* or gestational*) N3 (hypertension* or high blood pressure*)) ) | Expanders - Apply equivalent subjects Search modes - Boolean/Phrase | Interface - EBSCOhost Research Databases Search Screen - Advanced Search Database - CINAHL Complete | 21,942 |
| S1 | ( (MH "Telemedicine+") OR "Telemedicine*" ) OR ( "Telehealth*" OR (MH "Telehealth+") ) OR ( TI ( "digital health" or online or telecommunication* or teleconferenc* or tele-conferenc* or apps or digital or ehealth or e-health or "electronic health record*" or emedicine or e-medicine or etherap* or e-therap* or "health technolog*" or ICT or "instant messag*" or "information technolog*" or internet or web or mhealth or m-health or MMS or mobile* or podcast* or smartphone* or "smart phone*" or SMS or "social media" or technolog* or telecare or tele-care or telehealth or tele-health or telemedicine or tele-medicine or telerehabilitation* or tele-rehabilitation* or virtual or web-based* or website* or "virtual reality" or "mobile health" or m-health or health ) OR AB ( "digital health" or online or telecommunication* or teleconferenc* or tele-conferenc* or apps or digital or ehealth or e-health or "electronic health record*" or emedicine or e-medicine or etherap* or e-therap* or "health technolog*" or ICT or "instant messag*" or "information technolog*" or internet or web or mhealth or m-health or MMS or mobile* or podcast* or smartphone* or "smart phone*" or SMS or "social media" or technolog* or telecare or tele-care or telehealth or tele-health or telemedicine or tele-medicine or telerehabilitation* or tele-rehabilitation* or virtual or web-based* or website* or "virtual reality" or "mobile health" or m-health or health ) ) OR ( TI ( android or app* artificial intelligence or AI or audio or blog or CBT or CD-ROM or "cell phone*" or cellphone or chat or computer* or cyber* or DVD or eHealth or e-health or "electronic health*" or e-Portal or ePortal or eTherap* or forum* or gaming or "information technolog*" or "instant messag*" or internet* or ipad or iphone or ipod or web* or WWW ) OR AB ( android or app* or audio or blog or CBT or CD-ROM or "cell phone*" or cellphone or chat or computer* or cyber* or DVD or eHealth or e-health or "electronic health*" or e-Portal or ePortal or eTherap* or forum* or gaming or "information technolog*" or "instant messag*" or internet* or ipad or iphone or ipod or web* or WWW ) ) OR ( TI ( "smart phone*" or smartphone or "social network* site*" or "mobile phone*" or email* or mobile or multimedia or online* or "personal digital assistant*" or PDA or SMS or "social medi*" or software or telecomm* or telehealth* or telemed* or telemonitor* or telephone or telepsych* or teletherap* or "text messag*" or texting or podcast or virtual* ) OR AB ( "smart phone*" or smartphone or "social network* site*" or "mobile phone*" or email* or mobile or multimedia or online* or "personal digital assistant*" or PDA or SMS or "social medi*" or software or telecomm* or telehealth* or telemed* or telemonitor* or telephone or telepsych* or teletherap* or "text messag*" or texting or podcast or virtual* ) ) | Expanders - Apply equivalent subjects Search modes - Boolean/Phrase | Interface - EBSCOhost Research Databases Search Screen - Advanced Search Database - CINAHL Com |  |

**Web of Science**

| Type | Search Query | Results |
| --- | --- | --- |
| Search | #2 AND #1 |  |
| Search | pre-eclamp* or preeclamp* or eclampsia* or "Hellp syndrome*" (Topic) OR ((Pregnan* or maternal* or gestational*) NEAR3 (hypertension* or high blood pressure*)) (Topic) |  |
| Search | TS=((  Telemedicine* or e-health* or "mobile health*" or m-health or Telehealth or ehealth* or "mobile health*" or "m-health" or Telehealth or e-health* OR ehealth* OR Telemedicine* OR "digital health" OR online OR telecommunication* OR teleconferenc* OR tele-conferenc* OR apps OR artificial intelligence or AI OR digital OR ehealth OR e-health OR "electronic health record*" OR emedicine OR e-medicine OR etherap* OR e-therap* OR "health technolog*" OR ICT OR "instant messag*" OR "information technolog*" OR internet OR web OR mhealth OR m-health OR MMS OR mobile* OR podcast* OR smartphone* OR "smart phone*" OR SMS OR "social media" OR technolog* OR telecare OR tele-care OR telehealth OR tele-health OR telemedicine OR tele-medicine OR telerehabilitation OR tele-rehabilitation OR virtual OR web-based OR website* OR "virtual realit*" or android or app* or audio or blog or CBT or CD-ROM or "cell phone*" or cellphone* or chat or computer* or cyber* or DVD or eHealth or e-health or "electronic health*" or e-Portal or ePortal or eTherap* or forum* or gaming or "information technolog*" or "instant messag*" or internet* or ipad or iphone or ipod or web* or WWW or "smart phone*" or "smartphone*" or "social network*" site* or "mobile phone*" or email* or mobile or multimedia or online* or "personal digital assistant*" or PDA or SMS or "social medi*" or software or telecomm* or telehealth* or telemed* or telemonitor* or telephone or telepsych* or teletherap* or text messag* or texting or podcast or virtual*)) |  |

**Google Scholar**

( pregnan* OR maternal* OR gestational* ) W/3 ( hypertension* OR "high blood pressure*" ) OR ( pre-eclamp* OR preeclamp* OR eclampsia* ) AND ( telemedicine* OR telehealth* OR "digital health" OR online OR telecommunication* OR teleconferenc* OR tele-conferenc* OR apps OR digital OR ehealth OR e-health OR "electronic health record*" OR emedicine OR e-medicine OR etherap* OR e-therap* OR "health technolog*" OR ict OR "instant messag*" OR "information technolog*" OR internet OR web OR mhealth OR m-health OR mms OR mobile* OR podcast* OR smartphone* OR "smart phone*" OR sms OR "social media" OR technolog* OR telecare OR tele-care OR telehealth OR tele-health OR telemedicine OR tele-medicine OR telerehabilitation* OR tele-rehabilitation* OR virtual OR web-based* OR website* OR "virtual reality" OR "mobile health" OR m-health OR health OR android OR app* OR audio OR artificial intelligence OR AI OR blog OR cbt OR cd-rom OR "cell phone*" OR cellphone OR chat OR computer* OR cyber* OR dvd OR ehealth OR e-health OR "electronic health*" OR e-portal OR eportal OR etherap* OR forum* OR gaming OR "information technolog*" OR "instant messag*" OR internet* OR ipad OR iphone OR ipod OR web* OR www OR "smart phone*" OR smartphone OR "social network* site*" OR "mobile phone*" OR email* OR mobile OR multimedia OR online* OR "personal digital assistant*" OR pda OR sms OR "social medi*" OR software OR telecomm* OR telehealth* OR telemed* OR telemonitor* OR telephone OR telepsych* OR teletherap* OR "text messag*" OR texting OR podcast OR virtual* )
