# Supplementary material for: eHealth and Hypertensive Disorders of Pregnancy: Systematic Review
Source: J Med Internet Res. 2025 Sep 10;27:e77064. doi: 10.2196/77064 (PMC12422594; doi:10.2196/77064)
Supplement: Multimedia Appendix 2 [file jmir-v27-e77064-s002.docx]

Appendix Two – Risk of Bias assessment


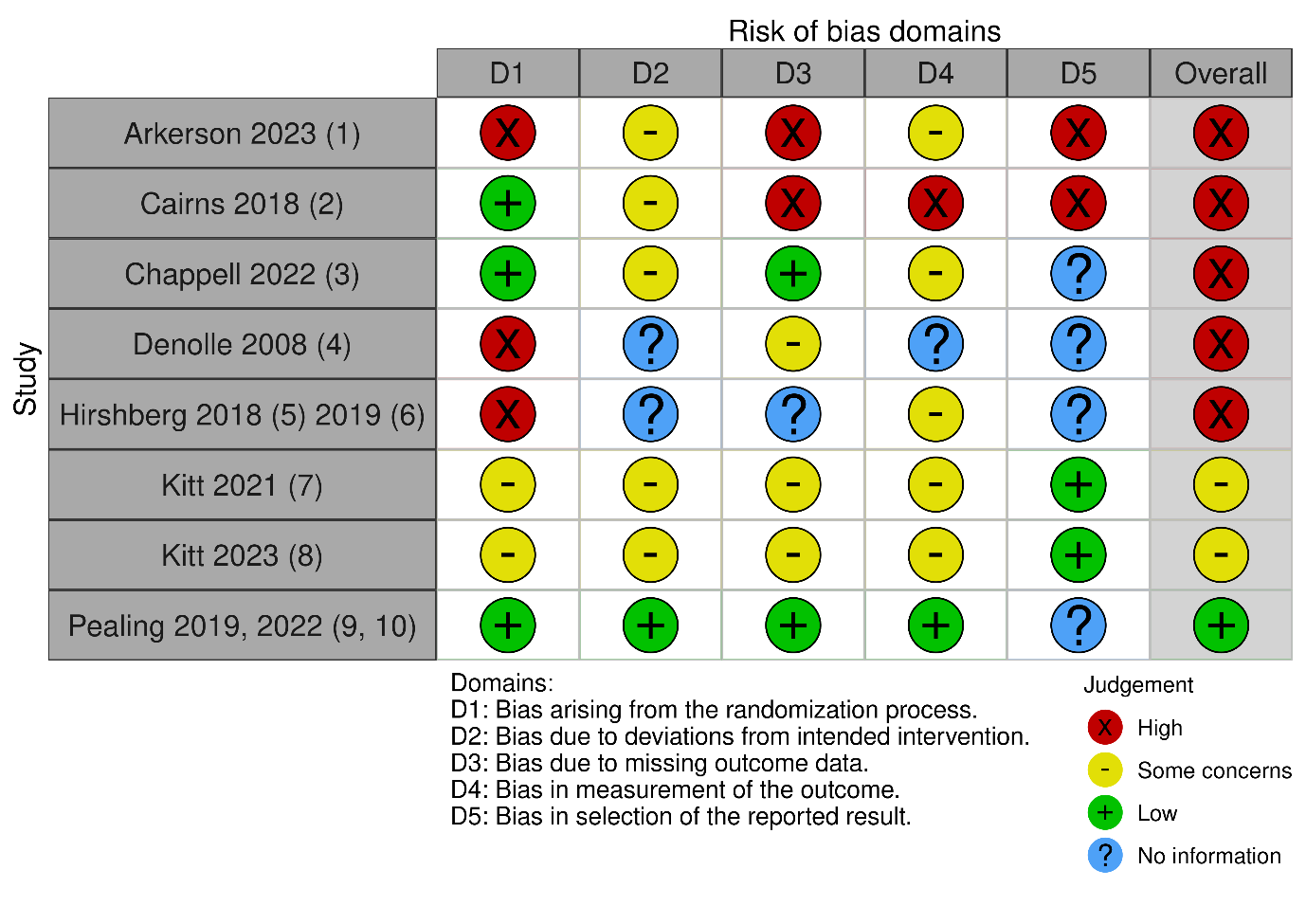


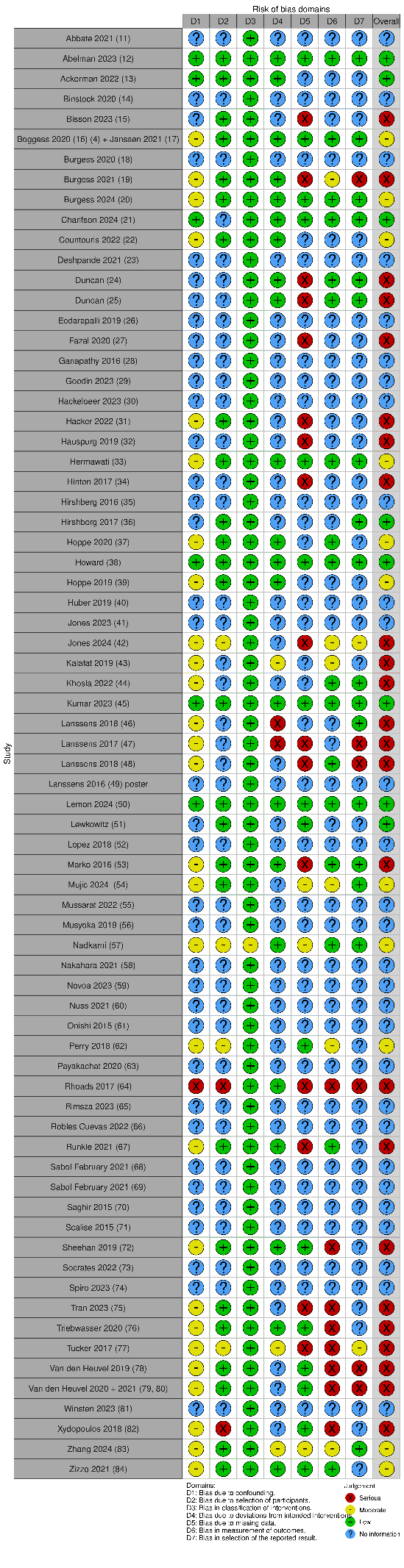


References

1. Arkerson BJ, Finneran MM, Harris SR, Schnorr J, McElwee ER, Demosthenes L, et al. Remote Monitoring Compared with In-Office Surveillance of Blood Pressure in Patients with Pregnancy-Related Hypertension: A Randomized Controlled Trial. Obstetrics and Gynecology. 2023;142(4):855-61.

2. Cairns AE, Tucker KL, Leeson P, Mackillop LH, Santos M, Velardo C, et al. Self-management of postnatal hypertension the SNAP-HT trial. Hypertension. 2018;72(2):425-32.

3. Chappell LC, Tucker KL, Galal U, Yu LM, Campbell H, Rivero-Arias O, et al. Effect of Self-monitoring of Blood Pressure on Blood Pressure Control in Pregnant Individuals With Chronic or Gestational Hypertension The BUMP 2 Randomized Clinical Trial. JAMA-JOURNAL OF THE AMERICAN MEDICAL ASSOCIATION. 2022;327(17):1666-78.

4. Denolle T, Weber JL, Calvez C, Getin Y, Daniel JC, Lurton O, et al. Diagnosis of white coat hypertension in pregnant women with teletransmitted home blood pressure. Hypertens Pregnancy. 2008;27(3):305-13.

5. Hirshberg A, Downes K, Srinivas S. Comparing standard office-based follow-up with text-based remote monitoring in the management of postpartum hypertension: a randomised clinical trial. BMJ Quality & Safety. 2018;27(11):871-7.

6. Hirshberg A, Sammel MD, Srinivas SK. Text message remote monitoring reduced racial disparities in postpartum blood pressure ascertainment. American Journal of Obstetrics and Gynecology. 2019;221(3):283-5.

7. Kitt JA, Fox RL, Cairns AE, Mollison J, Burchert HH, Kenworthy Y, et al. Short-Term Postpartum Blood Pressure Self-Management and Long-Term Blood Pressure Control: A Randomized Controlled Trial. Hypertension. 2021;78(2):469-79.

8. Kitt J, Fox R, Frost A, Shanyinde M, Tucker K, Bateman PA, et al. Long-Term Blood Pressure Control After Hypertensive Pregnancy Following Physician-Optimized Self-Management: The POP-HT Randomized Clinical Trial. Jama. 2023;330(20):1991-9.

9. Pealing LM, Tucker KL, Mackillop LH, Crawford C, Wilson H, Nickless A, et al. A randomised controlled trial of blood pressure self-monitoring in the management of hypertensive pregnancy. OPTIMUM-BP: A feasibility trial. Pregnancy Hypertension. 2019;18:141-9.

10. Pealing L, Tucker KL, Fletcher B, Lawley E, Chappell LC, McManus RJ, et al. Perceptions and experiences of blood pressure self-monitoring during hypertensive pregnancy: A qualitative analysis of women's and clinicians' experiences in the OPTIMUM-BP trial. Pregnancy Hypertension. 2022;30:113-23.

11. Abbate M, Srinivas SK, Triebwasser JE. 911 Readmission for hypertension among women in a postpartum remote blood pressure monitoring program. American Journal of Obstetrics and Gynecology. 2021;224(2 Supplement):S566.

12. Abelman SH, Svetec S, Felder L, Boelig RC. Impact of telehealth implementation on diagnosis of hypertensive disorders of pregnancy. Am J Obstet Gynecol MFM. 2023;5(8):101043.

13. Ackerman CM, Spatz E, Son M, Lundsberg LS, Culhane JF, Chou J, et al. Improving follow-up by enhancing access to care for postpartum hypertensive patients. American Journal of Obstetrics and Gynecology. 2022;226(1 Supplement):S213-S4.

14. Binstock A, Lemon L, Hauspurg A, Larkin J, Watson A, Quinn B, et al. 129: The effect of a remote blood pressure monitoring program on postpartum healthcare utilization. American Journal of Obstetrics and Gynecology. 2020;222(1 Supplement):S98-S9.

15. Bisson C, Dautel S, Mueller A, Britt R, Patel E, Suresh S, et al. Patient and provider perception of home blood pressure monitoring kits. Pregnancy Hypertension. 2023;34:33-8.

16. Boggess K, Demers S, Bailey S, Srinivas SK, You W, Grobman WA, et al. 720: Home blood pressure (BP) monitoring in postpartum women with hypertensive disorders of pregnancy. American Journal of Obstetrics and Gynecology. 2020;222(1 Supplement):S456-S7.

17. Janssen MK, Demers S, Srinivas SK, Bailey SC, Boggess KA, You W, et al. Implementation of a text-based postpartum blood pressure monitoring program at 3 different academic sites. American Journal of Obstetrics & Gynecology MFM. 2021;3(6):100446.

18. Burgess A, Gartrell K, Anderson T. Remote Monitoring of Blood Pressure After Preeclampsia. JOGNN-JOURNAL OF OBSTETRIC GYNECOLOGIC AND NEONATAL NURSING. 2020;49(6):S17-S8.

19. Burgess A, Gartrell K, Anderson T. Feasibility of Using Blood Pressure Self-Monitoring and the Epic MyChart Blood Pressure Flowsheet to Monitor Blood Pressure After Preeclampsia. CIN-COMPUTERS INFORMATICS NURSING. 2021;39(8):432-8.

20. Burgess A, Deannuntis T, Wheeling J. Postpartum Remote Blood Pressure Monitoring Using a Mobile App in Women with a Hypertensive Disorder of Pregnancy. MCN Am J Matern Child Nurs. 2024;49(4):194-203.

21. Charifson M, Wen T, Zell B, Vaidya P, Rios CI, Fagbohun F, et al. 352 Remote blood pressure monitoring during pregnancy: Comparing patient engagement between connected and unconnected device users. American Journal of Obstetrics & Gynecology. 2024;230(1):S198.

22. Countouris M, Jaramillo Restrepo V, Bidani S, Catov J, Berlacher K, Jeyabalan A, et al. Feasibility of Utilizing Telehealth in a Multidisciplinary Postpartum Hypertension Clinic. Womens Health Rep (New Rochelle). 2022;3(1):877-86.

23. Deshpande SS, Gadappa SN, Badgire SA, Sholapure AS, Kamble MS, Baxy HS. Study of Feasibility of Blood Pressure Monitoring in Postpartum Women by Teleconsultation in COVID 19 Pandemic Situation. J Obstet Gynaecol India. 2022;72(Suppl 1):186-91.

24. Duncan C, Bisson C, Mueller A, Costello L, Lang D, Rana S. Postpartum readmissions after implementation of a remote patient monitoring program for hypertension. Pregnancy Hypertension. 2024;36:31.

25. Duncan C, Patel E, Bisson C, Mueller A, Costello L, Lang D, et al. Six-week blood pressure trends in postpartum patients utilizing remote patient monitoring program. Pregnancy Hypertension. 2024;36:44-5.

26. Eedarapalli P, Stephenson E, Dell S. Florence (Flo) tele-monitoring of hypertension in pregnancy: A tool for the 21st century. BJOG: An International Journal of Obstetrics and Gynaecology. 2019;126(Supplement 2):157-8.

27. Fazal N, Webb A, Bangoura J, El Nasharty M. Telehealth: improving maternity services by modern technology. BMJ OPEN QUALITY. 2020;9(4).

28. Ganapathy R, Grewal A, Castleman JS. Remote monitoring of blood pressure to reduce the risk of preeclampsia related complications with an innovative use of mobile technology. Pregnancy Hypertension. 2016;6(4):263-5.

29. Goodin A, Dhillon K, Jennings-Coulibaly A, Roussos-Ross D, Varma D, Wen T. Real-Time Assessment of Symptoms for Postpartum Care via a Smartphone Application: Pilot Evaluation. Obstetrics and Gynecology. 2023;141(5 Supplement 1):65S.

30. Hackeloeer M, Hoyler A, Kaban N, Rieger O, Neznansky M, Lorenz-Meier L, et al. PO9_06. Evaluation of home blood pressure self-measurements in a high-risk collective: remote monitoring can help to predict adverse outcomes in pregnant women at risk. Pregnancy Hypertension. 2023;33(Supplement 1):e50-e1.

31. Hacker FM, Jeyabalan A, Quinn B, Hauspurg A. Implementation of a universal postpartum blood pressure monitoring program: feasibility and outcomes. AMERICAN JOURNAL OF OBSTETRICS & GYNECOLOGY MFM. 2022;4(3).

32. Hauspurg A, Lemon LS, Quinn BA, Binstock A, Larkin J, Beigi RH, et al. A Postpartum Remote Hypertension Monitoring Protocol Implemented at the Hospital Level. OBSTETRICS AND GYNECOLOGY. 2019;134(4):685-91.

33. Hermawati DM, Inda; Aida Fitri, Harahap; Juwita, Ratna. The Impact of Animation Video on Pre-eclampsia Prevention

Knowledge and Motivation in Pregnant Women. J Liaquat Uni Med Health Sci. 2024.

34. Hinton L, Tucker KL, Greenfield SM, Hodgkinson JA, Mackillop L, McCourt C, et al. Blood pressure self-monitoring in pregnancy (BuMP) feasibility study; a qualitative analysis of women's experiences of self-monitoring. BMC Pregnancy & Childbirth. 2017;17:1-9.

35. Hirshberg A, Srinivas S. Text message based remote monitoring in the management of postpartum hypertension. Obstetrics and Gynecology. 2016;127(Supplement 1):131S.

36. Hirshberg A, Bittle MD, VanDerTuyn M, Mahraj K, Asch DA, Rosin R, et al. Rapid-cycle innovation testing of text-based monitoring for management of postpartum hypertension. Journal of Clinical Outcomes Management. 2017;24(2):77-85.

37. Hoppe KK, Thomas N, Zernick M, Zella JB, Havighurst T, Kim K, et al. Telehealth with remote blood pressure monitoring compared with standard care for postpartum hypertension. American Journal of Obstetrics and Gynecology. 2020;223(4):585-8.

38. Howard E, Gillispie-Bell V, Olet S, Glenn B, Ammar N, Price-Haywood EG. Evaluating Racial Disparities in Implementation and Monitoring of a Remote Blood Pressure Program in a Pregnant Population-A Retrospective Cohort Study. Ochsner J. 2024;24(1):22-30.

39. Hoppe KK, Williams M, Thomas N, Zella JB, Drewry A, Kim K, et al. Telehealth with remote blood pressure monitoring for postpartum hypertension: A prospective single-cohort feasibility study. Pregnancy Hypertension. 2019;15:171-6.

40. Huber K, Schellhammer S, Fox C, Taylor A. Implementation of a Home Health-Telemedicine Program to Monitor Pregnant Women With Preeclampsia. JOGNN-JOURNAL OF OBSTETRIC GYNECOLOGIC AND NEONATAL NURSING. 2019;48(3):S22-S.

41. D. Jones R, Allison MK, Moody H, Peng C, Eswaran H. Use of Cellular-Enabled Remote Patient Monitoring Device for Hypertension Management in Pregnant Women: A Feasibility Study. Maternal & Child Health Journal. 2023;27(7):1191-8.

42. Jones RD, Peng C, Jones CD, Long B, Helton V, Eswaran H. Cellular-Enabled Remote Patient Monitoring for Pregnancies Complicated by Hypertension. Cardiovasc Digit Health J. 2024;5(3):156-63.

43. Kalafat E, Leslie K, Bhide A, Thilaganathan B, Khalil A. Pregnancy outcomes following home blood pressure monitoring in gestational hypertension. Pregnancy Hypertension. 2019;18:14-20.

44. Khosla K, Suresh S, Mueller A, Perdigao JL, Stewart K, Duncan C, et al. Elimination of racial disparities in postpartum hypertension follow-up after incorporation of telehealth into a quality bundle. American Journal of Obstetrics & Gynecology MFM. 2022;4(3):100580.

45. Kumar NR, Arias MP, Leitner K, Wang E, Clement EG, Hamm RF. Assessing the impact of telehealth implementation on postpartum outcomes for Black birthing people. Am J Obstet Gynecol MFM. 2023;5(2):100831.

46. Lanssens D, Vonck S, Storms V, Thijs IM, Grieten L, Gyselaers W. The impact of a remote monitoring program on the prenatal follow-up of women with gestational hypertensive disorders. European Journal of Obstetrics & Gynecology and Reproductive Biology. 2018;223:72-8.

47. Lanssens D, Vandenberk T, Smeets CJ, De Cannière H, Molenberghs G, Van Moerbeke A, et al. Remote Monitoring of Hypertension Diseases in Pregnancy: A Pilot Study. JMIR Mhealth Uhealth. 2017;5(3):e25.

48. Lanssens D, Vandenberk T, Smeets CJP, De Cannière H, Vonck S, Claessens J, et al. Prenatal Remote Monitoring of Women With Gestational Hypertensive Diseases: Cost Analysis. Journal of Medical Internet Research. 2018;20(3):28-.

49. Lanssens D, Van Moerebeke A, van den Hoogen A, Geusens N, Grieten L, Gyselaers W. E4. Remote prenatal follow-up of patients at risk for gestational hypertensive disorders: maternal and neonatal outcomes. Journal of Maternal-Fetal and Neonatal Medicine. 2016;29(sup2):24.

50. Lemon LS, Quinn B, Binstock A, Larkin JC, Simhan HN, Hauspurg A. Clinical Outcomes Associated With a Remote Postpartum Hypertension Monitoring Program. Obstet Gynecol. 2024;144(3):377-85.

51. Lewkowitz AK, Hauspurg A. Perinatal Remote Blood Pressure Monitoring. Obstet Gynecol. 2024;144(3):339-45.

52. Lopez BDB, Aguirre JAA, Coronado DAR, Gonzalez PA, editors. Wearable technology model to control and monitor hypertension during pregnancy. Iberian Conference on Information Systems and Technologies, CISTI; 2018.

53. Marko KI, Krapf JM, Meltzer AC, Oh J, Ganju N, Martinez AG, et al. Testing the Feasibility of Remote Patient Monitoring in Prenatal Care Using a Mobile App and Connected Devices: A Prospective Observational Trial. JMIR RESEARCH PROTOCOLS. 2016;5(4).

54. Mujic E, Parker SE, Nelson KP, O'Brien M, Chestnut IA, Abrams J, et al. Implementation of a Cell-Enabled Remote Blood Pressure Monitoring Program During the Postpartum Period at a Safety-Net Hospital. J Am Heart Assoc. 2024;13(13):e034031.

55. Mussarat N, Biggio JR, Rick T, Elmayan A, Williams F. Connected MOM: A Remote Blood Pressure Monitoring System for Early Detection of Pregnancy Associated Hypertension. American Journal of Obstetrics and Gynecology. 2022;226(1 Supplement):S740-S1.

56. Musyoka FM, Thiga MM, Muketha GM. A 24-hour ambulatory blood pressure monitoring system for preeclampsia management in antenatal care. Informatics in Medicine Unlocked. 2019;16 (no pagination).

57. Nadkarni S, Oyama S, May H, Adeyemo O. Quality of Blood Pressure Monitoring During Telehealth Visits for Pregnant Patients at Risk of Preeclampsia. Telemed J E Health. 2024;30(8):2165-72.

58. Nakahara A, Shuffle E, Omachi K, Blake Z, Mohammed A, Elmayan A, et al. 357 Digital medicine to increase compliance for hypertensive disorders of pregnancy: a retrospective cohort study. American Journal of Obstetrics and Gynecology. 2021;224(2 Supplement):S233.

59. Novoa B, Patel E, Costello L, Watts B, Suresh S, Bisson C, et al. PO7_1. Patient perceptions regarding remote patient monitoring for postpartum hypertension. Pregnancy Hypertension. 2023;33(Supplement 1):e41.

60. Nuss E, Bank TC, Holleran D, Hoffman M. 900 The impact of postpartum blood pressure monitoring amongst women with hypertensive disorders of pregnancy. American Journal of Obstetrics and Gynecology. 2021;224(2 Supplement):S558.

61. Onishi S, Nakano K, Iwai K, Yamada Y, Akasaka J, Shigemitsu A, et al. Postpartum follow-up of hypertensive pregnancy using at-home web-linked mobile sphygmomanometer. Pregnancy Hypertension. 2015;5(1):85.

62. Perry H, Sheehan E, Thilaganathan B, Khalil A. Home blood-pressure monitoring in a hypertensive pregnant population. ULTRASOUND IN OBSTETRICS & GYNECOLOGY. 2018;51(4):524-30.

63. Payakachat N, Rhoads S, McCoy H, Dajani N, Eswaran H, Lowery C. Using mHealth in postpartum women with pre-eclampsia: Lessons learned from a qualitative study. International Journal of Gynecology & Obstetrics. 2020;149(3):339-46.

64. Rhoads SJ, Serrano CI, Lynch CE, Ounpraseuth ST, Gauss CH, Payakachat N, et al. Exploring Implementation of m-Health Monitoring in Postpartum Women with Hypertension. TELEMEDICINE AND E-HEALTH. 2017;23(10):833-41.

65. Rimsza RR, Goyal S, Barry VG, Oakes MC, Turnbull D, Sabol B, et al. Factors affecting engagement in a postpartum remote blood pressure monitoring program: Identifying opportunities for improvement. American Journal of Obstetrics and Gynecology. 2023;228(1 Supplement):S728.

66. Robles Cuevas MA, López Martínez I, López Domínguez E, Hernández Velázquez Y, Domínguez Isidro S, Flores Frías LM, et al. Telemonitoring System Oriented towards High-Risk Pregnant Women. Healthcare (2227-9032). 2022;10(12):2484.

67. Runkle JD, Sugg MM, McCrory S, Coulson CC. Examining the Feasibility of Smart Blood Pressure Home Monitoring: Advancing Remote Prenatal Care in Rural Appalachia. Telemedicine Reports. 2021;2(1):125-34.

68. Sabol B, Oakes MC, Turnbull D, Raghuraman N, Kelly J, Carter EB, et al. 925 Implementation of postpartum home blood pressure monitoring to reduce readmissions for hypertensive disorders of pregnancy. American Journal of Obstetrics and Gynecology. 2021;224(2 Supplement):S574.

69. Sabol B, Oakes MC, Turnbull D, Raghuraman N, Kelly J, Carter EB, et al. 879 Utilization of postpartum home blood pressure monitoring across different obstetrical care settings. American Journal of Obstetrics and Gynecology. 2021;224(2 Supplement):S545-S6.

70. Saghir S, Rajasri A. 'Tele-health service' for home blood pressure and symptom monitoring in the management of postnatal pre-eclampsia in a district general hospital with remote geography- a pilot study. BJOG-AN INTERNATIONAL JOURNAL OF OBSTETRICS AND GYNAECOLOGY. 2015;122:24-.

71. Scalise LF, Stringer M. Follow-up Text Messages for Patients at High Risk of Postpartum Hypertension...Proceedings of the 2015 AWHONN Convention. JOGNN: Journal of Obstetric, Gynecologic & Neonatal Nursing. 2015;44:S6-S.

72. Sheehan E, Khalil A, Kay L. Using a smartphone app to identify signs of pre-eclampsia and/or worsening blood pressure. British Journal of Midwifery. 2019;27(2):92-9.

73. Socrates T, Wenker C, Blaschke C, Vischer A, Meienberg A, Mayr M, et al. Feasibility, Acceptance and Safety of a Home Based Telemonitoring Strategy in Women with Postpartum Hypertension. Interim Analysis of the Swiss Register for Women with Ppht. Journal of Hypertension. 2022;40(Supplement 1):e274-e5.

74. Spiro L, Bairey Merz CN, Bello N, Kilpatrick S, Minissian M, Wei J. Remote Patient Monitoring for Postpartum Hypertensive Disorders of Pregnancy [ID: 1378058]. Obstetrics & Gynecology. 2023;141(5S):68S.

75. Tran KC, Freiman S, Chaworth T, Purkiss S, Foster C, Khan NA, et al. Implementation of a Home Blood Pressure Monitoring Program for Management of Hypertensive Disorders of Pregnancy, a Quality Improvement Study, in British Columbia, Canada. Obstetric Medicine. 2023;16(1 Supplement):17-8.

76. Triebwasser JE, Janssen MK, Hirshberg A, Srinivas SK. Successful implementation of text-based blood pressure monitoring for postpartum hypertension. PREGNANCY HYPERTENSION-AN INTERNATIONAL JOURNAL OF WOMENS CARDIOVASCULAR HEALTH. 2020;22:156-9.

77. Tucker KL, Taylor KS, Crawford C, Hodgkinson JA, Bankhead C, Carver T, et al. Blood pressure self-monitoring in pregnancy: examining feasibility in a prospective cohort study. BMC Pregnancy & Childbirth. 2017;17:1-10.

78. van den Heuvel JFM, Kariman SS, van Solinge WW, Franx A, Lely AT, Bekker MN. SAFE@HOME - Feasibility study of a telemonitoring platform combining blood pressure and preeclampsia symptoms in pregnancy care. European Journal of Obstetrics & Gynecology & Reproductive Biology. 2019;240:226-31.

79. van den Heuvel JFM, Lely AT, Huisman JJ, Trappenburg JCA, Franx A, Bekker MN. SAFE@HOME: Digital health platform facilitating a new care path for women at increased risk of preeclampsia - A case-control study. Pregnancy Hypertens. 2020;22:30-6.

80. van den Heuvel JFM, van Lieshout C, Franx A, Frederix G, Bekker MN. SAFE@HOME: Cost analysis of a new care pathway including a digital health platform for women at increased risk of preeclampsia. Pregnancy Hypertension. 2021;24:118-23.

81. Winsten MT, Gold S, Overcash R. MomHEART: Utilization of a Web-Based Postpartum Hypertension Platform. Obstetrics and Gynecology. 2023;141(5 Supplement 1):64S.

82. Xydopoulos G, Perry H, Sheehan E, Thilaganathan B, Fordham R, Khalil A. Home blood-pressure monitoring in a hypertensive pregnant population: cost-minimization study. Ultrasound in Obstetrics & Gynecology. 2019;53(4):496-502.

83. Zhang Y, Lin YY, Lal L, Swint JM, Tucker T, Ivory DM, et al. Feasibility of Remote Blood Pressure Monitoring for Detection and Management of Maternal Hypertension in a predominantly Black, Rural and Medicaid Population in Mississippi. Telemed J E Health. 2024;30(7):e2096-e102.

84. Zizzo AR, Hvidman L, Salvig JD, Holst L, Kyng M, Petersen OB. Home management by remote self-monitoring in intermediate- and high-risk pregnancies: A retrospective study of 400 consecutive women. Acta Obstetricia et Gynecologica Scandinavica. 2022;101(1):135-44.
