# Supplementary material for: eHealth and Hypertensive Disorders of Pregnancy: Systematic Review
Source: J Med Internet Res. 2025 Sep 10;27:e77064. doi: 10.2196/77064 (PMC12422594; doi:10.2196/77064)
Supplement: Multimedia Appendix 3 [file jmir-v27-e77064-s003.docx]

Appendix 3 – Results Summary

Patient recruitment/ consent rate

| Study | eHealth intervention | Outcome |
| --- | --- | --- |
| Patients at risk of HDP |  |  |
| Hacker 2022 [88]. | Remote BP monitoring with telehealth communication | Participation with answering the screening phone call ranged from 49% to 64% |
| Van den Heuvel 2019 [81]. | Remote BP monitoring with BP cuff that transmits data to mobile phone application via Bluetooth with storage on web based platform | 14/ 33 women (42%) agreed to participate |
| Patients with HDP |  |  |
| Boggess 2020 [50] Janssen 2021 [62]. | Remote BP monitoring with transmission of data via web based platform and text message communication | 199 (65%) of 305 potentially eligible women enrolled |
| Deshpande 2021[53]. | Remote BP monitoring with text message communication | 63/156 (40.38%) women agreed to participate in the study, three women opted out of the study before 90 days |
| Eedarapalli 2019[54]. | Remote BP monitoring with text messaging of BPs linked with web based platform | 100% completed feedback forms |
| Hirshberg 2018 [61]. | Remote BP monitoring with text message communication, data storage on web based platform | 72/278 women (74%) agreed to participate |
| Hoppe 2019 [74]. | Remote BP monitoring with tablet device and blood pressure cuff (Bluetooth transmission of data) with telehealth review | Amongst 263 eligible women, 124 agreed to be approached for this study, resulting in a recruitment rate of 47%. 55 out of 124 approached participants gave informed consent, resulting in a consent rate of 44%. 52 participants completed the 6 week clinic visit, resulting in a retention rate of 95% |
| Pealing 2019 [63]. | Remote BP monitoring with data submission via text message or smart phone application with web based platform data storage | Overall recruitment 71% - 158 randomised, 222 eligible |
| Sabol February 2021 [64]. | Remote BP monitoring with text message communication and storage of data on web based portal | 278 women were eligible for enrolment, 247 participants enrolled (88.8%) with a 92.7% completion rate |
| Socrates 2022 [93]. | Remote BP monitoring with transmission of data via smartphone application with telehealth communication | 98% (out of 112) participants chose eHealth management – home based BP telemonitoring |
| Payakachat [30] | Remote BP monitoring with Bluetooth transmission of data to web based platform with telehealth communication | Of the 48 participants, 37 (77.1% completion rate) completed the interview. Twenty-one (84% response rate) were from the user group and 16 (69.6%) were from the non-user group |
| Sheehan 2019 [112]. | Remote BP monitoring with BP cuff with storage of data via smartphone application | Postal invitations were sent to 48 eligible women, 17 responses were received, with 11 women agreeing to participate and 6 declining to participate |
| Thomas 2021 [80]. | Remote BP monitoring with tablet device and blood pressure cuff (Bluetooth transmission of data) with telehealth review | 66% of respondents completed the survey |
| Tran 2023 [121]. | Remote BP monitoring with communication of recorded BPs via email | 43/103 participants (response rate 41%) completed the qualitative survey (antepartum (40%) and postpartum (60%)) |
| Nadkarni [40] | Telehealth | Of 721 eligible patients, 200 (27.7%) had only 1 ante- or  postpartum telehealth visit, while 244 (33.8%) had ‡2 ante- or postpartum telehealth visits. Of the 444 patients who  had ‡1 ante- or postpartum telehealth visit, 404 (91.0%) had ‡1 antepartum telehealth visit, 141 (31.8%) had ‡1 postpartum telehealth visit, and 101 22.7%) had ‡1 ante- and postpartum telehealth visits. Of the 404 patients with ‡1 antepartum telehealth  visit, 198 (49.0%) had 1 antepartum telehealth visit, 121 (30.0%) had 2–3, and 85 (21.0%) had >3. Of the 141 patients with ‡1 postpartum telehealth visit, 121 (85.8%) had 1 postpartum telehealth visit and 20 (14.2%) had >1. Patients with more antepartum ( p < 0.001) and postpartum visits ( p = 0.020) were more likely to have had ‡1 telehealth visit |
| Zhang [86] | Remote BP monitoring with Bluetooth BP cuff, communication via cellular enabled iPad tablet and telehealth and storage of data via web based platform | 100 patients were referred to the program, with 98 patients consenting to enrol (95 in person and 3 virtually).  Seventy-seven (N= 77) patients actively participated after receiving kits and instructions. Among them, 21 (21.43%) fully adhered to the prenatal protocol, 36 (36.73%) mostly adhered, and 21 (21.43%) partially adhered. The remaining 21  (21.43%) patients did not use RPM |

Admission rate

| **Study** | **Comparator** | **Outcome** |
| --- | --- | --- |
| **Antenatal** |  |  |
| Lanssens 2018 [91] | Conventional care (no eHealth) | The number of prenatal admissions (44/86 [51.16%] versus 154/215 [71.63%], respectively, b = –1.23) and prenatal admissions until delivery (27/86 [31.40%] versus 124/215 [57.67%], respectively, b = –1.24) were lower in the RM group than in the CC group in both the uni- and multivariate analyse |
| Lanssens 2017 [83] | Conventional care (no eHealth) | The number of prenatal hospital admissions and admissions until delivery were lower in RM than in CC when a univariate analysis is performed: 56.25% (27/48) versus 74.49% (73/98), and 27.08% (13/48) versus 62.24% (61/97). This was not significant in multivariate analysis |
| Perry [114] | Historical data | There was no significant difference between the HBPM group and controls in the number of days of blood pressure-related hospital admissions. Days of BP-related hospital bed stay per patient eHealth 4 (2-6), conventional care 5 (2-6.8) p=0.2 |
| van den Heuvel 2020 [15] | Conventional care (no eHealth) – historical cohort | In the SAFE@HOME group, observational admissions for hypertension  or diagnosis/exclusion of suspected preeclampsia were significantly  lower compared to the control group (2.9% vs 13.5% of participants, p = 0.004.– van den Heuvel 2020 |
| Zhang [86] | No comparator | During pregnancy, 21 (21.43%) patients were admitted to study sites, with 7 from the fully adhered group, 6 from the mostly adhered group, 6 from the partially adhered group, and  2 from the nonadhered group. Among them, 6 were admitted for gestational hypertension, with 3 from the fully adhered group, 1 from the mostly adhered group, 1 from the partially adhered group, and 1 from the nonadhered group. No statistical significance was observed in these numbers across the different adherence groups |
| **Postnatal** |  |  |
| Arkerson 2021 [48] | Conventional care (no eHealth) | Readmission within 10 days – remote monitoring n=4 (4.2%) vs. conventional care n=5 (5%) p =0.792 |
| Cairns [51] | Conventional care (no eHealth) | Eleven serious adverse events were reported: all were readmissions to hospital with no serious maternal morbidity or mortality. Three were unrelated to BP. Eight women were readmitted to hospital with high or low BP, all within the first 2 weeks postpartum, 5 from the eHealth group (3 from conventional care) |
| Binstock 2020 [49] | Historical cohort | Remote BP monitoring reduced readmissions - 41 (12.2%) women with remote BP monitoring compared with 31 (8.0%) women without remote BP monitoring in the historical cohort |
| Hacker 2022 [88] | No comparator | 8 (0.7%) women were diagnosed with severe preeclampsia requiring hospital readmission |
| Hirshberg 2023 [96] | Two comparator groups – Cohort A - same hospitals historical cohort (conventional care) and Cohort C - contemporaneous different hospitals conventional care | Hospital readmissions were 50% and 56.7% lower for the eHealth group compared to two other cohort groups . Inpatient admissions eHealth 12 (1.2%) Cohort A 23 (2.2%) difference -11 (-50%) p=0.6 OR 0.52 (0.26-1.04), eHealth 17 (1.3%) Cohort C 38 (3%) -21 (-56.7%) p=0.005 OR 0.44 (0.25-0.78) |
| Hirshberg 2019 [60] | Conventional care (no eHealth) | There were no hypertension readmissions in the texting arm, compared to 4 readmissions in usual care |
| Hoppe 2020 [75]. | Conventional care (no eHealth) | Readmissions in eHealth group compared to controls - 1 [0.5%] vs 8 [3.7%]; relative risk [RR], 0.12; 95% CI, 0.01-0.96 |
| Hirshberg 2018 [61] | Conventional care (no eHealth) | There was a statistically significant increase in hypertension-related readmissions in the office arm (3.9% vs 0%, p=0.04) |
| Sabol February 2021 [33]. | Historical cohort | The percentage of readmissions secondary to hypertension decreased by 24% between pre and post implementation of remote BP monitoring - 62.2% vs. 38.1% p=0.08 |
| Khosla 2022[90] | Historical data | No difference in readmissions between telehealth and usual care - 17.84% vs 17.44% P=.91 |
| Nuss 2021 [109] | Conventional care (no eHealth) | Only one study found that readmission increased with eHealth compared to usual care - 4.4% vs. 2.3%, p<0.01. After adjusting for age, race, and preterm delivery, this difference remained - women in the eHealth group had an 87% higher odds of needing readmission (p < 0.001) |
| Deshpande 2021[53] Hirshberg 2016 [59]  Socrates 2022 [93]  Spiro 2023 [94]  Hoppe 2019 [74] | No comparator | No readmissions during study period |
| Saghir 2015 [92] | Historical data | No readmissions during study period |
| Boggess 2020 [50] Janssen 2021 [62] | No comparator | 10 (5%) women had a hospital readmission for hypertension |
| Abbate 2021 [47] | No comparator | 18/877 patients – 2.1% per year |
| Lewkowitz [45] | Remote BP monitoring with manual submission of data and usual care | Manual BP and Automatic BP patients were equally  likely to have a HTN-related ED presentation or hospital readmission. |
| Lemon [70] | Conventional care (no eHealth) | Program participation was  consistently associated with decreased hospital readmission. Results demonstrate 1 less readmission for every 100 patients engaged in the program (propensity  score–matched adjusted risk difference [aRD] 21.5, 95% CI, 22.6 to 20.46), with no significant difference in ED visits |
| Kitt 2023 [84] | Conventional care (no eHealth) | There were 41 hospital readmissions in the 14 days after discharge. Thirty-seven were related to hypertension with a lower number in the intervention group 8 (7%) vs control 29 (27%) p<0.001. Relative risk reduction for blood pressure-related readmissions in the intervention group was 72.5% |
| Duncan [119] | No comparator | 45.4 % patients had a diagnosis of preeclampsia at  enrolment. 21.6 % were readmitted within six weeks of delivery, with similar readmission rates between Black and non-Black patients  (21.7 %vs 21.3 %, p=0.93). The majority of patients were readmitted with elevated home BPs (39.4 %), followed by severe preeclampsia  (22.7 %) and postpartum headache (13.6 %). The majority of patients had severe hypertension on readmission (57.6 %) |
| Burgess [68] | Historical cohort at 3 (of the 5 hospitals intervention utilised at) hospitals | In the control cohort, 812 patients had a pregnancy complicated by a hypertensive disorder, and 10.1% (*n* = 82) of them were evaluated in the ED, placed under observation, or were readmitted in the postpartum for a hypertensive complaint. In the eHealth cohort 837 patients had a pregnancy complicated by a hypertensive disorder of pregnancy of which 12.3% (*n* = 103) had an ED evaluation, observation, or readmission in the. When comparing all types of visits (readmissions,  ED evaluations, and observation, pre- and postimplementation),  there was no significant increase in visit types (*p* = .16). There was, however, a significant increase  in readmissions post-implementation 3.1% (*n* = 25) versus 5% (*n* = 42; *p* = .047) |

Outpatient adherence (outpatient follow up attendance rate)

| Study | Comparator | Outcome |
| --- | --- | --- |
| Antenatal |  |  |
| Xydopoulos 2018 [113] | Conventional care (no eHealth) | Remote BP monitoring with a BP cuff and app increased the duration of monitoring (9 weeks vs 5weeks, P =0.004) and those patients started monitoring at an earlier gestational age (30weeks vs 33.6weeks, P =0.001) |
|  |  |  |
| Postnatal |  |  |
| Binstock 2020 [49] | Historical cohort (no eHealth) | Attendance at the postpartum appointment was significantly higher in the remote monitoring group 297 (87.8%) vs. 301 (79%) in historical cohort, p=0.004 |
| Hauspurg 2019 [57] | Historical cohort (no eHealth) | 360 (88%) women receiving eHealth intervention attended a 6-week postpartum visit, compared with a historical rate of 60% attendance among all deliveries and 66% attendance among women with a hypertensive disorder of pregnancy in the year prior to implementation of remote patient monitoring |
| Hirshberg 2018 [61] | Historical cohort (no eHealth), contemporaneous conventional care (no eHealth) | 87 (84%) patients in the text message based remote surveillance group had a recorded blood pressure at follow up compared to the previous 2 year average of 30% attendance rate at the hospital postnatal high-risk blood pressure clinic. The direct comparator group (conventional office visit) had 45 women (44%) attend their postnatal office visit |
| Khosla 2022[90] | Historical data | Among all the patients, adherence with at least one post partum hypertension clinic visit increased significantly when telehealth visits were performed (53.5% vs 76.7%; P<.0001). Comparing African American and Caucasian patients – the African American patients had a significant improvement in visit adherence with telehealth visits - 48.5% vs 76.3%; P<.0001 |
| Nakahara 2021 [118] | Conventional care (no eHealth) | Patients enrolled in remote BP monitoring with a digital BP cuff were more likely to adhere to postpartum BP evaluation – postpartum BP evaluation eHealth Yes n=250 (59.5%) No n=170 (40.5%) conventional care Yes n=1304 (41.5%) No n=1842 (58.6%) |
| Patel 2023 [97] | Historical cohort (no eHealth) | Home BP monitoring with telematic data transmission to care providers the postpartum BP check appointment attendance rate was 76.7%, after the intervention the attendance rate improved to 83.1%. At the 6 week postpartum visit attendance improved from 52% to 60.3% |
| Sanghavi 2022 [34] | Conventional care (no eHealth) | In postpartum patients with preeclampsia - the adherence rate was 32% for in-person clinic visits compared to 70% for telemedicine visits (p < 0.001) |
| Hirshberg 2016 [59] | No comparator | 6/32 patients returned for their usual care postpartum office BP check |
| Ackerman 2022 [72] | Conventional care (no eHealth) | The number of days until postpartum follow-up was statistically significantly shorter in the remote BP monitoring group with telehealth follow up (mean of 4 days) compared to those in the control group (mean of 7 days). 94% in the intervention group saw a health care provider within 10 days of discharge vs. 65% in the usual care group (OR 7.48 (95% CI 1.71-32.70)) |
| Rhoads 2016 [79] | Conventional care (no eHealth) | Adherence with study visits in eHealth users - 88% (107/121), compared to 90% (68/76) in non users. Follow up at the 6 week postnatal study visit users 72% (38/53) vs. control 73% (22/30) |
| Countouris 2022[52] | Conventional care (no eHealth) | Of 175 scheduled visits, 140 patients were seen in the clinic, corresponding with a show rate of 80.0%. 122 of visits were scheduled virtually with a 75.4% show rate and 53 visits were scheduled in-person with a 90.6% show rate. |
| Kumar [100] | Historical cohort prior to telehealth visits (no eHealth) | In both unadjusted and adjusted analyses, Black patients were less likely to attend a PPV than non-Black patients before telehealth implementation (63.9% vs 88.7%; P≤.001; aOR: 0.48, 95% CI:0.29−0.79). After tele-health implementation, Black patients were still less likely than non-Black patients to attend a PPV on bivariate analysis (79.1% vs 88.6%; P<.001), but after controlling for insurance, education, parity, and scant prenatal care, there was no longer a significant difference in PPV attendance by race (aOR, 0.74; 95%CI,0.45−1.21). |
| Lemon [70] | Usual care (no eHealth intervention) | Patients enrolled in the  remote monitoring program were more likely to  attend an office visit within 6 weeks postpartum  (77.0% vs 64.4%, P,.001). There were six more ndividuals attending an office visit within 6 weeks postpartum (propensity score–matched aRD 5.7, 95% CI, 3.9–7.6). |

Participation rate (BP ascertainment)

| Study | Comparator | Outcome |
| --- | --- | --- |
| Antenatal |  |  |
| Marko 2016 [108]. | Conventional care (no eHealth) | Remote patient monitoring increased the mean number of blood pressure measurements (34, P=.01) compared to the number of blood pressure measurements collected in the office (10) |
| Pealing 2019 [63]. | Conventional care (no eHealth) | There was high persistence with the self-monitoring via BP cuff and app with 86% (43/50) of women with chronic and 76% (38/49) of women with gestational hypertension provided home BP readings for 80% or more of the time |
| Runkle 2021 [110] | No comparator | 23/30 patients contributed to remote BP monitoring, mean days of home BP monitoring was 16.2 days |
| Tucker 2022 [98]. | Usual care (no eHealth intervention) | There was no difference in sending first BP through - mean 104.3 days (eHealth with remote BP monitoring and app group) vs. 106.2 days (usual care) |
| Tucker 2017 [66]. | No comparator | 162 (81%) women provided self-monitored BP data |
| Van den Heuvel 2019 [81]. | No comparator | The compliance rate was 93% for blood pressure uploads in a remote BP monitoring program |
| Van den Heuvel 2020 [15]. | Historical data | The median compliance rate for blood pressure measurements was 91.2% (IQR 70–100, range 34–100) |
| Zizzo 2021 [99]. | No comparator | Very few (<10) women did not participate with home-monitoring management because of non-compliance, or because of technical issues with the equipment. The longest duration of inclusion was 194 days; however, the median  varied from 12.5 to 30 days |
| Charifson [44] | Remote BP monitoring with manual reporting of values to web based platform | Patients with connected devices had more mean entries/day (0.57 vs. 0.35) and higher adherence rates (15% vs. 8%) compared to  those with unconnected devices. Adjusted analyses noted 2.28 (95% CI: 1.44-3.61) times more BP measure per day and 6.28 (95% CI: 2.62-15.05) higher odds of adherence among connected (eHealth intervention)  versus unconnected device users |
| Howard [69] | Conventional care (no eHealth) | Patients in the CMOM group, irrespective of race, had  1.8 times more BP measurements than usual care patients during pregnancy (26 vs 15 measurements; rate ratio=1.78, 95% CI 1.74-1.82). Patients enrolled in CMOM had shorter intervals between BP measurements compared to usual care patients (12 vs 17 days, *P<*0.001). During the postpartum period, patients in the CMOM group who had been diagnosed with hypertensive disorders  of pregnancy, irrespective of race, had more BP measurements than usual care patients (6 vs 5 measurements;  rate ratio=1.30, 95% CI 1.23-1.37), with statistically significant differences seen among both Black and White patients. Patients in CMOM and usual care had similar  intervals (7 days) between BP measurements during the 6-  week postpartum period. Patients with a diagnosis of hypertensive disorder of pregnancy who enrolled in CMOM were modestly more likely to take their BP within 7 days of discharge from the hospital than usual care patients (71.6% vs 67.6%, respectively, risk ratio=1.06, 95% CI 1.00-1.11); however, the CMOM intervention  did not result in an improvement in 7-day postpartum adherence for Black patients (risk ratio=1.03, 95% CI 0.94-1.11) as it did for White patients (risk ratio=1.09, 95% CI 1.01-1.17) |
| Nadkarni [40] | No comparator | Of the 244 patients with ‡2 ante- or postpartum telehealth  visits, 142 (58.2%) had documentation of owning a home blood pressure cuff. However, only 106 (43.4%) had  ‡1 SMBP documented in their telehealth visit notes. patients who attended federally qualified health centres were more likely to have had ‡1 documented SMBP during their telehealth visit compared with patients who attended the MFM clinic ( p = 0.018) or private clinics ( p < 0.001). Patients with more antepartum ( p = 0.032)  and postpartum telehealth visits ( p = 0.017) were also more likely to have ‡1 documented SMBP during telehealth. |
| Postnatal |  |  |
| Arkerson 2021 [48] | Conventional care (no eHealth) | Patients in the remote monitoring arm had significantly higher rates of blood pressure ascertainment within 10 days of discharge (91.7% vs 58.4%, P=0.001). Patients in the remote monitoring arm had significantly higher rates of blood pressure ascertainment within 10 days of discharge (91.7% vs 58.4%, P=0.001) |
| Hoppe 2020 [75] | Conventional care (no eHealth) | More women in the intervention group had one BP measured within 10 days postpartum when compared with the controls (202 [94.4%] vs 129 [60.3%]; RR, 1.59; 95% CI, 1.36-1.77) |
| Boggess 2020 [50] Janssen 2021 [62] | No comparator | 190 (96%) of 199 women recorded at least one BP. 131 (66%) recorded a BP within the first 3-4 days, 167 (84%) within 7-10 days, and 124 (62%) recorded a BP during both times |
| Burgess 2020 [102] | No comparator | 69% used an automatic BP cuff and app for remote BP monitoring |
| Cairns 2018 [51] | Conventional care (no eHealth) | 82/91 participants (90%) self-monitored their BP and finished follow-up |
| Hirshberg 2023 [96] | Conventional care (no eHealth) | 90% of patients texted at least one blood pressure reading, and more than 80% of patients sent in multiple blood pressure readings |
| Hirshberg 2016 [59] Hirshberg 2017 [67] | No comparator | 27/32 patients sent at least one BP – 27 patients sent at least one BP, 10/32 texted BPs on 5 of the 7 days, 27/32 texted at least one BP reading on day 1 or 2 and 21/32 texted at least one BP on day 5, 6 or 7 |
| Hirshberg 2019 [60] | Conventional care (no eHealth) | Text message based remote BP monitoring resulted in >90% blood pressure ascertainment in participants |
| Hirshberg 2018 [61]. | Historical data | Text based remote BP monitoring increased the rate of at least one BP measurement compared to office visits 92.2% vs 43.7%; p<0.001 |
| Onishi 2015 [29]  Antenatal and postnatal | No comparator | The compliance of the patients for the routine measurement of their blood pressure was notably good as reported with no specific figures. |
| Rimsza 2023 [32] | No comparator | 108 (63.2%) patients using remote BP monitoring with text message transmission of data had had high engagement (responding to >50% of prompts) and 63 (36.8%) had low engagement (responding to <50% of prompts). |
| Sabol 2021 [64] | No comparator | Adherence rate of 92.1% and 84.7% adherence with at least 50% of BP reminders. |
| Scalise 2015 [65]. | No comparator | The response rate was 34% (n=41) |
| Spiro 2023 [94]. | No comparator | Overall adherence/ participation rate was 91%, 100% (total 30 patients) of participants completed the program |
| Tran 2023 [121].  Antenatal and postnatal | No comparator | Median adherence to remote BP monitoring in antenatal and postnatal patients was 0.94, IQR 0.57, 1.00 |
| Triebwasser 2020 [42]. | Other eHealth intervention (implementation of same eHealth intervention at different institution), Conventional care (no eHealth) | BP measurements occurred in 318 (95.5%) in the remote BP monitoring with app group versus 95 (92.2%) in the usual care cohort. In the eHealth cohort, 282 (84.7%) checked BP in the first 10 days postpartum versus 84 (81.6%) in the usual care cohort. This difference was not seen after adjusting for confounders (adjusted OR 0.89 [95% CI 0.48, 1.64]). Adherence to the program in the first 6 months varied from 93.9 to 100% with proportion of patients having BP checks in the first 10 days postpartum varied from 80.3 to 89.7% |
| Winsten 2023 [36]. | No comparator | 49 (76.6%) participants interacted with the eHealth intervention (remote BP monitoring with web based platform and text message or phone call reminders) |
| Burgess [68] | Historical cohort (no eHealth) | 74% (n=938) patients entered seven or more BPs. 6% (n=79) patients entered zero or just one BP. |
| Lemon [70] | Conventional care (no eHealth) | Patients enrolled in the  remote monitoring program were more likely to  have a BP recorded at 10 days or less from the time  of delivery (97.6% vs 11.5%, P,.001), and be at 6  weeks postpartum (80.2% vs 60.9%, P,.001). Adherence to hypertension specific  guidelines increased, indicating 85 more individuals  with a BP recorded within 10 days of delivery (propensity score–matched aRD 85.4, 95% CI, 84.3, 86.6) for every 100 engaged in the program |
| Lewkowitz [45] | Remote BP monitoring with manual submission of data and usual care | Compared to those in Manual-BP, those in Automatic-BP had more SMBP assessments (median 20 [interquartile range [11, 38] vs median 12 [IQR 4, 28; MD 0.5; 95% CI 0.04]) |
| Mujic [71] | No comparator | Over the 6-week program,  patients measured their BP an average of 15.2 days (SD: 10.2), with minimal differences by race and ethnicity. During the first week postpartum, >90% of patients recorded at least 1 BP measure with no difference in  engagement between race and ethnicity subgroups.  Although there was retention in participation at each  postpartum week, we observed similar rates of program fatigue week to week among racial and ethnic  groups, with roughly half of all patients engaging with  the program for the full duration. |
| Tully [101] | Conventional care (no eHealth) | The proportion of documented BP evaluation at 7–10 days postpartum was higher in the intervention compared to standard  care (76.0% vs 58.0%, absolute risk difference  18.0%, 95% CI −0.1 to 36.1%), but the difference was not statistically significant (chi square p=0.06). Individuals  enrolled in the study were more likely to undergo postpartum BP evaluation (67.0% overall) than observed prior to the  project (20%). All the interviewed participants in the intervention reported evaluating their BP at home. Additionally, a 6/24 (25.0%) of the interviewed participants in standard care  reported home BP monitoring, which they conducted with their own devices outside of the study. |

**Patient satisfaction, qualitative assessment of patient experience**

| Study | eHealth intervention | Outcome |
| --- | --- | --- |
| Arkerson 2021 [48] | Remote BP monitoring with BP cuff and smartphone application, text messages alerts were used as well as telephone review if required | 96.4% participants enrolled in remote monitoring were either comfortable or very comfortable with home blood pressure monitoring |
| Bisson 2023 [87] | Remote BP monitoring with BP cuff with telehealth review of blood pressure logs | Most of the 117 patients who responded to the survey found Cuff Kits “very valuable” or “valuable” 68.4 % and 19.7 %, respectively |
| Hauspurg 2019[57] | Remote BP monitoring with web based platform and text message communication | 235 patients (94%) reported that they were satisfied with their experience in the remote monitoring program and 205 women (82%) felt more comfortable knowing a nurse was checking their health remotely every day. 239 patients (96%) reported that they are comfortable using new technology |
| Burgess 2020 [102] | Remote BP monitoring linked with web based platform and mobile phone application | Based on a Likert scale (1-5), the participants felt that the education provided was very helpful (4.86), the cuff was easy to use (4.94), and that it would be easy to take their BP twice daily once home |
| Denolle 2008[46] | Remote BP monitoring linked with web based platform | 75% participants were very satisfied and 25% satisfied with remote BP monitoring and transmission of data to a central database |
| Deshpande 2021[53] | Remote BP monitoring with text message communication | 50 out of 60 (83.3%) were satisfied with home monitoring of blood pressure by teleconsultation. No patient was unsatisfied with teleconsultation |
| Eedarapalli 2019[54] | Remote BP monitoring with text messaging of BPs linked with web based platform | 100% liked the system and were satisfied |
| Hoppe 2019 [74] | Remote BP monitoring with tablet device and blood pressure cuff (Bluetooth transmission of data) with telehealth review | 90% of the patients found measuring their BP at home acceptable and feasible. 35/56 participants completed the survey and all of them rated their experience with self-monitoring as ‘good’ or ‘excellent’. Overall, 39/52 (87%) were “very” or “extremely” satisfied with the remote monitoring |
| Jongsma 2020[77] | Remote BP monitoring with Bluetooth transmission of data to a smartphone application and web based platform | 47/51 92% were satisfied with usability of mHealth technology |
| Lopez 2018 [78] | Remote BP monitoring with a wearable device with data transmission to a phone application with data storage on a web based platform. The wearable device transmits information via the internet and Bluetooth | Participants using a wearable BP device connected to an app found the technology non-invasive and easy to use with friendly graphics and alerts |
| Marko 2016 [108] | Remote BP monitoring with mobile phone application connected to a wireless weight scale and BP cuff | The patient satisfaction survey was completed by 6/8 participants (75%). These participants felt comfortable with the concept and technical aspects of remote monitoring, were able to easily access provider resources and were easily able to remember to use the remote monitoring program |
| Musyoka 2019 [27] | Remote BP monitoring with smartwatch linked via Bluetooth with a mobile with transmission and storage of information with a mobile phone application and web based platform | Participants thought highly of the mobile and app based remote BP monitoring - perceived usefulness 100%, perceived ease of use 90%, user satisfaction 100%. |
| Novoa 2023 [28] | Remote BP monitoring BP cuff with automatic transmission of data to mobile phone application communication and web based platform | 96% reported that they were “definitely able” or “somewhat able” to improve their blood pressure management with use of remote BP monitoring with telematic data transmission. 85% of patients reported they were “very” or “somewhat” likely to attend their postpartum follow-up visit. 81% of patients reported they felt “very” or “somewhat” likely to recommend remote BP monitoring to others |
| Pealing 2022 [31] | Remote BP monitoring with automated BP monitor, communication via text message or smartphone application. BPs were transmitted automatically to a web based platform | Women were very positive about their experiences of eHealth monitoring of BP with automated BP monitor and data transmission via text message or study app and preferred self monitoring over routine clinic BP measurement and would choose to self-monitor in any future pregnancies. |
| Spiro 2023 [94] | Remote BP monitoring with automatic uploading of data to a web based platform with communication via mobile phone application and telehealth | Satisfaction rate was 92% |
| Thomas 2021 [80] | Remote BP monitoring with tablet device and blood pressure cuff (Bluetooth transmission of data) with telehealth review | 59% (75/127) reported eHealth easily (very or extremely) fit in their lifestyle, 80% (34/43) felt help was readily accessible when needed, and 80% (101/127) of women felt confident using a tablet and Bluetooth BP monitor. Overall, 84% (107/127) reported that they were very or extremely satisfied with the equipment |
| Van den Heuvel 2020 [136] | Remote BP monitoring with automated BP monitor with transmission of data via Bluetooth to web based portal with communication via mobile phone application or email | The online survey on a eHealth BP monitoring platform was answered by 51 (49%) participants. Few had difficulties with using the system (4%, 2/51) and instructions regarding the use of the BP monitor and app were clear to almost all (96%, 49/51). The vast majority was satisfied with the use of the app and platform (92%, 47/51). The vast majority of participants using remote BP monitoring was satisfied with the use of the app and platform (92%, 47/51). |
| Zhang [86] | Remote BP monitoring with Bluetooth BP cuff, communication via cellular enabled iPad tablet and telehealth and storage of data via web based platform | Thirty-eight patients completed the satisfaction survey at discharge, and most expressed high satisfaction with various  aspects of the program, including the telemonitoring kit, the education provided, communication with the nurse. The comments received further reflected  positive feedback and satisfaction, and recommending its participation to others |
| Lewkowitz [45] | Remote BP monitoring with Bluetooth BP cuff with data transmission via mobile phone application utilising AI to respond to BPs or symptoms with communication with clinicians via email | SMBP programs were viewed highly favorably, with median  Likert scale scores of 10 and low decisional regret in both groups |
| Kitt 2023 [84] | Remote blood pressure monitoring Bluetooth BP cuff with communication with clinician via mobile phone app, storage of data on web based platform | Quality of life scores did not differ significantly between groups |
| Tully [101] | Remote BP monitoring with telephone review with clinician | Participants rated their health care experiences and the study materials positively  Participant accounts  suggested remote BP monitoring was acceptable  with themes of (1) maternal convenience coupled with uncertainty, (2) inadequate access to postpartum health information, and (3) determining the need for acute care utilization, which occurred (4) in the context of their daily postpartum lives. |
| Burgess [68] | Antenatal and postpartum education via mobile phone app, remote self BP monitoring with communication via mobile phone app, text message or email, and telehealth review | Forty-seven participants completed the patient satisfaction survey. 93%  (*n* = 39) of patients who used the app and completed  the survey felt it was easy to use the BP monitor and get  started with the app. Seventy percent (*n* = 33) reported that they would be more likely to return to their care team in the future for a pregnancy because they offer at-home BP monitoring. 70% (*n* = 33) felt that the postpartum monitoring program gave them extra peace of mind during their first few weeks at home. Several respondents reported they liked the text  reminders to take their BP. |
| Jones [39] | Remote BP monitoring with cellular enabled BP devices supported by telehealth communication and storage of data on web based platform | Participants described the advantages of using the RPM BP device, including (1) easy/convenient to use, (2) perceived better care, (3) increased monitoring of BP, (4) call center support, and (5) participant empowerment.  While most participants reported no issues with using the device, a few reported issues with the call center protocol or perceiving that the device gave higher BP readings when compared to clinical BP monitors. Some participants reported feeling like their BP readings were inaccurate. One participant reported a negative effect on their anxiety when their BP was shown to be high |

Carer experience

| Study | eHealth intervention | Outcome |
| --- | --- | --- |
| Irani poster [122] | Remote BP monitoring, interviews of patient support person via telephone | Almost all participants reported a positive perception  of home BP monitoring, despite lacking a detailed understanding of medical benefits. Most supported the pregnant woman’s autonomy  to monitor her BP at home. However, many felt that family involvement was needed prior to seeking medical care for elevated values. |

Patient experience – ease of use

| Study | eHealth intervention | Outcome |
| --- | --- | --- |
| Burgess 2021 [103] | Remote BP monitoring with data transmission via smartphone application and web based platform | All participants felt it would be very easy to remotely monitor their BP (median, 5 on Likert scale) |
| Rhoads 2016 [79] | Remote BP monitoring with data transmission via Bluetooth to a web based platform | There was no difference in perceived ease of use, perceived satisfaction, or perceived benefits between full or partial users of eHealth remote BP monitoring |
| Boggess 2020 [50] Janssen 2021 [62] | Remote BP monitoring with transmission of data via web based platform and text message communication | 98 (49%) of women completed the survey where almost all participants thought it was easy to send and receive texts. 92 (94%) agreed that ‘Texts helped to pay attention to BP’, and 91 (93%) agreed ‘I would recommend this program to a friend or family member’ |
| Denolle 2008[46] | Remote BP monitoring linked with web based platform | 92% of the women described HBP as very easy and 8% as easy to use and the installation of the monitor was considered very easy for 95% of the pregnant women |
| Ganapathy 2016 [56] | Remote BP monitoring with transmission of data via Bluetooth with communication via mobile phone application, text message and management of data via web based platform | >90% of the women reported that it was very simple to use |
| Hirshberg 2016 [59] Hirshberg 2017 [67] | Remote BP monitoring with electronic BP cuffs with text message communication | Patients reported satisfaction with the text messaging system with most commenting on the convenience of remote BP monitoring – “this was a lot better than having to pay for the bus and waiting for hours in some waiting room.” One patient also reported that the increased awareness was important, stating that “when [she] got home and realized that [her blood pressure] was still high, [she] did her own research and learned more about hypertension and preeclampsia.” Others reported that they checked their blood pressure after the study time , and “would have went longer than a week if they had asked me to.” |
| Lanssens 2019 [82] | Remote BP monitoring with wireless BP monitor with data transmission via Wi-Fi or Bluetooth to a web based platform with follow up via telehealth | 81% (N = 45) patients completed the satisfaction survey. 41 (91%) reported the amount of mental effort required to interact with the telehealth technology was “not at all” or “a little,” 42 (93%) felt “quite a bit” or a “great deal” of security using the home monitoring system, 44 (98%) of participants reported it took 1 min to record their blood pressure. 49/51 (91%) felt comfortable using mHealth. 49/51 96% found it easy to learn how to use mHealth technology. Nearly all participants 49/51 96% considered the BP monitor instructions to be clear and understandable. Nearly three-quarters (34/47, 72%) of the participants reported that they had no problems with taking the measurements at the requested times. Most (42/47, 89%) participants felt remote monitoring of BP was important to the follow up of their pregnancy |
| Pealing 2022 [31] | Remote BP monitoring with automated BP monitor, communication via text message or smartphone application. BPs were transmitted automatically to a web based platform | Women reported finding it “very simple” to learn how to measure their BP and the instructions were “simple, straight forward“ |
| Tran 2023 [121] | Remote BP monitoring with communication of recorded BPs via email | Almost all (98%) found home based BP monitoring with BP cuff and text message and online platform transmission and storage of information easy to do, with 51% were able to adhere to their monitoring schedule |
| Van den Heuvel 2019 [81] | Remote BP monitoring with BP cuff that transmits data to mobile phone application via Bluetooth with storage on web based platform | Participants reported that the BP monitor was easy to use with the usability of the BP monitor rated an average of 8.9 on a 1–10 scale (range 8–10), the app/web portal 7.6 (range 5–10) and the content of the app 8.0 (range 7–10) |
| Thomas 2021 [80] | Remote BP monitoring with tablet device and blood pressure cuff (Bluetooth transmission of data) with telehealth review | 1.6% (2/  128) of women felt the instructions for use were very or extremely difficult, 0.9% (1/128) of women felt the technology  required an extreme amount of mental effort |

Patient experience – privacy concerns

| Study | eHealth intervention | Outcome |
| --- | --- | --- |
| Hauspurg 2019[57] | Remote BP monitoring with communication via text message and data transmission to a web based platform | 221 women (88%) did not worry about their privacy with the technology |
| Lanssens 2019 [82] | Remote BP monitoring with wireless BP monitor with data transmission via Wi-Fi or Bluetooth to a web based platform with follow up via telehealth | Most (41/47, 87%) participants did not have any negative concerns about privacy, while 3 mothers reported that sharing health data posed a threat to their privacy |
| Thomas 2021 [80] | Remote BP monitoring with tablet device and blood pressure cuff (Bluetooth transmission of data) with telehealth review | 88% (112/127) of women felt secure transmitting protected health information, and 90% (114/127) felt they had sufficient control over their data |

Patient experience – recommendation to others

| Study | eHealth intervention | Outcome |
| --- | --- | --- |
| Hauspurg 2019[57] | Remote patient monitoring with web based platform and text message communication | 232 (93%) would recommend the program to others |
| Fazal 2020[55] | Remote BP monitoring with text message communication with web based platform storage of information | 100% would recommend remote BP monitoring with text message reminders to friends and family |
| Hoppe 2019 [74] | Remote BP monitoring with tablet device and blood pressure cuff (Bluetooth transmission of data) with telehealth review | 42 (93%) would recommend telehealth to other women |
| Jongsma 2020[77] | Remote BP monitoring with Bluetooth transmission of data to a smartphone application and web based platform | 45/51 88% would recommend to friends and family |
| Thomas 2021 [80] | Remote BP monitoring with tablet device and blood pressure cuff (Bluetooth transmission of data) with telehealth review | 91% (115/127) of women responding that they would recommend eHealth (tablet and Bluetooth BP monitor) to other postpartum women |
| Van den Heuvel 2019 [81] | Remote BP monitoring with BP cuff that transmits data to mobile phone application via Bluetooth with storage on web based platform | All participants (13/13) would recommend telemonitoring by the blood pressure monitor and app to other patients |
| Van den Heuvel 2020 [15] | Remote BP monitoring with automated BP monitor with transmission of data via Bluetooth to web based portal with communication via mobile phone application or email | Parous participants were more likely to recommend eHealth BP monitoring to other women (96.9% of multiparous vs. 73.7% of nulliparous women) |

Patient experience – preference for eHealth compared to conventional models of care

| Study | eHealth intervention | Outcome |
| --- | --- | --- |
| Ganapathy 2016 [56] | Remote BP monitoring with transmission of data via Bluetooth with communication via mobile phone application, text message and management of data via web based platform | 78% of women reported that they would prefer home monitoring with the device compared to the traditional model of care of hospital visits or home visits by midwives |
| Jongsma 2020[77] | Remote BP monitoring with Bluetooth transmission of data to a smartphone application and web based platform | 38 (84%) would prefer telehealth to going to a hospital or clinic |
| Thomas 2021 [80] | Remote BP monitoring with tablet device and blood pressure cuff (Bluetooth transmission of data) with telehealth review | 95% of women preferred remote care for postpartum follow-up. Only 4.7% (6/128) of women preferred to go to the hospital or clinic instead of using technology at home |
| Pealing 2022 [31] | Remote BP monitoring with automated BP monitor, communication via text message or smartphone application. BPs were transmitted automatically to a web based platform | All women interviewed from the intervention group said they would choose to self-monitor in any future pregnancies. One woman from the usual care group reported that she would not plan to use eHealth; she had not needed antihypertensive medication and felt the clinic readings were sufficiently frequent. Several women in the same study also preferred to have both home and clinic monitoring in any future pregnancy |
| Runesha [85] | Remote BP monitoring with Bluetooth BP cuff, communication with mobile phone app and telehealth clinician review | 28.0% of the patients who responded reported a preference for in-person visits over telehealth visits. |

Patient experience – benefits of eHealth

| Study | eHealth intervention | Outcome |
| --- | --- | --- |
| Hinton 2017[58] | Remote BP monitoring with automated BP cuff that transmits data to web based platform as well as via text message | Reduction of anxiety |
| Eedarapalli 2019 [54] | Remote BP monitoring with transmission of data to a web based platform as well as via text message | Good communication (30% participants) |
| Payakachat 2020 [30] | Remote BP monitoring with Bluetooth transmission of data to web based platform with telehealth communication | Decreased stress and anxiety, opportunity to use daily (and check their own blood pressure) and to self-manage, it was convenient. Participants felt it was beneficial, easy to use and advantageous to be able to self-manage their condition |
| Hirshberg 2016 [59]Hirshberg 2017 [67] | Remote BP monitoring with electronic BP cuffs with text message communication | Convenient, cost saving for the patient (reduced transport cost), increased awareness of hypertension, preeclampsia and symptoms |
| Lopez 2018 [78] | Remote BP monitoring with a wearable device with data transmission to a phone application with data storage on a web based platform. The wearable device transmits information via the internet and Bluetooth | Sense of empowerment |
| D Jones 2023 [89] | Remote BP monitoring with BP cuff that transmits data via cellular capability to a web based platform with telehealth communication | Perception that their care was better, BP being remotely monitored by a health professional, increased participant empowerment, convenience, and ease of use of the device |
| Lanssens 2019 [82] | Remote BP monitoring with wireless BP monitor with data transmission via Wi-Fi or Bluetooth to a web based platform with follow up via telehealth | 39 of the 47 (83%) mothers reported that remote BP monitoring with eHealth gave them a feeling of safety |
| Socrates 2022 [93] | Remote BP monitoring with transmission of data via smartphone application with telehealth communication | At 3 months, 97.8% participants reported a feeling of safety with home-based telemonitoring and would use the same approach in the future |
| Marko 2016 [108] | Remote BP monitoring with mobile phone application connected to a wireless weight scale and BP cuff | Most (83%, 5/6) of the participants felt that the remote BP monitoring app assisted with healthy pregnancy-related behaviours, were satisfied with prenatal care, felt more connected with their provider, and felt more knowledgeable about their pregnancy |
| Novoa 2023 [28] | Remote BP monitoring BP cuff with automatic transmission of data to mobile phone application communication and web based platform | 80% of the patients felt that they were “much more” or “somewhat more” aware of their own health after use of the remote BP monitoring |
| Pealing 2022 [31] | Remote BP monitoring with automated BP monitor, communication via text message or smartphone application. BPs were transmitted automatically to a web based platform | Participants using eHealth to self monitor their BP felt that it improved knowledge of their own BP and health, particularly in the context of interpreting any symptoms, as well as increase awareness of the variability of BP. Participants also preferred eHealth over conventional treatment due to convenience, reassurance, a sense of control and the hope that it would lead to more valid measurements |
| Robles Cuevas 2022 [115] | Remote telemonitoring system via a mobile phone application | Main advantages of the eHealth system reported to include the ability to access the information regarding their pregnancy treatment through the clinical history consultation, and to record alarm symptoms, record hypertension symptom data, record clinical analyses/medical studies, view recommendations provided by the doctor, and the generation of alerts that are sent to the doctor, to detect risk situations to the patient. |
| Runkle 2021 [110] | Remote BP monitoring with BP cuff and smartphone application with transmission and storage of data via web based platform | Participants (93%, n = 25) using eHealth to monitor their own BP and transmit the data via app were willing to change their behavior during pregnancy in response to personalized recommendations |
| Saghir 2015 [92] | Remote BP and symptom monitoring with automated tele device with telehealth assessment | Patient satisfaction  rate was 92% due to regular reassurance from daily monitoring  and avoidance of travel |
| Sheehan 2019 [112] | Remote BP monitoring with BP cuff with storage of data via smartphone application | Control over their care and continuity of care was what women valued the most from remote BP monitoring and a mobile phone app to transmit data |
| Tran 2023 [121] | Remote BP monitoring with communication of recorded BPs via email | 48% of participants were reassured by home measurements |
| Van den Heuvel 2019 [81] | Remote BP monitoring with BP cuff that transmits data to mobile phone application via Bluetooth with storage on web based platform | The platform was considered useful to gain more insight in BP trend (77%, 10/13), to feel involved in prenatal care (85%, 11/13) and to feel engaged in care participation (77%, 10/13 |
| Huber 2019 [76] | Remote BP monitoring with storage of data via Bluetooth capable iPad and communication via telehealth | eHealth also allowed for early identification of worsening disease process with early identification of concerning features such as increased weight gain, medication adjustments, referrals to licensed clinical social workers for living conditions– postpartum depression, and referral to the emergency department for severe-range blood pressures |
| Runesha [85] | Remote BP monitoring with Bluetooth BP cuff, communication with mobile phone app and telehealth clinician review | 92.8% of the entire cohort felt confident with the use of the mobile application and blood pressure cuff and 85.1% responded that they thought the RPM program would improve management of their blood pressures at home. At 3 weeks postpartum 87.4% patients reported that they were more likely to attend postpartum follow-up visits with the use of the RPM program. At 6 weeks postpartum 90.6% of  the patients that responded felt ‘much more’ or ‘somewhat more’ in control/aware of their own health. 77.4% of patients that responded felt that the RPM program was ‘very easy’ or ‘somewhat easy’ to fit into their lifestyle. 84.6% of the patients that responded report that it was ‘very easy’ or ‘somewhat easy’ to access their healthcare provider with the RPM program. Among all  respondents, 94.0% of patients expressed that they were ‘definitely’ or ‘somewhat likely’ to recommend the RPM  program to other postpartum mothers with HDP. 72.7% of patients reported being ‘very satisfied’ with the telehealth and postpartum RPM program. |

Patient knowledge

| Study | eHealth intervention | Outcome |
| --- | --- | --- |
| Hermawati [116] | Cohort of patients pre eHealth intervention (video) and post intervention, control cohort not shown eHealth intervention who received usual care/ information | The average value (mean) of  knowledge in the pretest and post-test of the intervention group is 16.13 and 19.19, respectively, and the mean value of knowledge in the pretest and post-test of the control group was 15.90 and 17.29 respectively. Then, the mean motivation scores in the  pretest and post-test of the intervention group were 42.68 and 44.74, respectively. The mean motivation scores in the pretest and post-test of the control group were 42.87 and 43.87, respectively.  Following the eHealth intervention there was a difference in the mean value of pretest and post-test knowledge and motivation (p value= 0,000, p value= 0,001), respectively. |
| Runesha [85] | Remote BP monitoring with Bluetooth BP cuff, communication with mobile phone app and telehealth clinician review | Greater than 85.5% of the  patients answered each question within the knowledge domain correctly, demonstrating an understanding of their  diagnosis, including potential warning signs of preeclampsia and indications for presenting to care. Participants showed  an understanding of the need for follow-up care, with 92.6% of the entire cohort agreeing that they still need a follow-up visit at 6-week postpartum, even if blood pressures normalized. Educational material was perceived as ‘very easy’ or ‘somewhat easy’ to understand by 89.1% of the entire cohort and 87.7% of the entire cohort definitely liked or somewhat liked the involvement of a nurse educator. |
| Irani [41] | Remote BP monitoring, audiovisual training of the use of the remote BP monitor and recognition of abnormal BP | Following audiovisual training of remote BP monitor - on objective assessment, out of eight displayed  BP monitor outputs, participants correctly identified a significantly  higher amount when provided with both the numbers and colors (μ=7.07, σ=0.88) as compared to the numbers only (μ=6.45, σ=1.40) with p=0.002 |

Patient experience – challenges and concerns regarding eHealth intervention

| Study | eHealth intervention | Outcome |
| --- | --- | --- |
| Jongsma [77] | Remote BP monitoring with Bluetooth transmission of data to a smartphone application and web based platform | Some participants found daily BP monitoring burdensome or found the timing to be difficult to integrate into their daily routine |
| Payakachat 2020 [30] | Remote BP monitoring with Bluetooth transmission of data to web based platform with telehealth communication | Some participants of the eHealth remote BP monitoring felt overwhelmed with the frequent communication with the call center and stressed with having to take and potentially retake their blood pressure daily |
| Pealing 2022 [31] | Remote BP monitoring with automated BP monitor, communication via text message or smartphone application. BPs were transmitted automatically to a web based platform | A couple of participants reported the system took time to work out and could be burdensome. |
| D Jones 2023 [89] | Remote BP monitoring with BP cuff that transmits data via cellular capability to a web based platform with telehealth communication | Concerns with the device itself, such as problems with wearing the device or perceiving that it gave higher readings than clinical BP monitors as well as concerns with the remote monitoring process, such as the alerts and calls from health professionals when readings were high |
| Tran 2023 [121] | Remote BP monitoring with communication of recorded BPs via email | The most common difficulties with adhering to BP measurement via eHealth included newborn care (57%), forgetting to check (39%), and lack of time in the mornings (35%) 21% participants found eHealth remote BP monitoring increased their anxiety |

Clinical outcomes

Incidence of hypertensive disorders of pregnancy

| Study | Comparator | Outcome |
| --- | --- | --- |
| Hacker 2022 [88] | No comparator | 8 (0.7%) of these women were diagnosed with severe preeclampsia. Of 510 women, 98 (8.2%) had a new diagnosis of a postpartum hypertensive disorder |
| Hoppe 2019 [74] | No comparator | 9 (16%) participants developed severe hypertension after discharge. 33 (60%) of participants had blood pressures over 150/100 mmHg that required treatment. 6 (11%) patients were referred to the emergency department for evaluation of symptomatic severe hypertension (e.g., headache, blurry vision, abdominal pain, shortness of breath) |
| Runkle 2021 [110] | No comparator | Incidence of preeclampsia: 1/23 patients. Incidence of gestational hypertension: 1/23 patients |
| Tucker 2017 [66] | No comparator | 20 (13%) developed gestational hypertension, of whom 5 (3%) went on to develop pre- eclampsia. Of the 13 (8%) women with chronic hypertension, 5 (3%) developed pre-eclampsia and 3 (2%) had worsening hypertension but not proteinuria. Incidence of gestational hypertension: 28/157 (18%) |
| Zizzo 2021 [99] | No comparator | There were no cases of eclampsia, incidence of preeclampsia: number of patients at high risk of PE n=151 total, 32 (21.2%) developed preeclampsia |
| Patel 2023 [97] | Historical data | eHealth intervention HDP incidence - Gestational HTN 83 (40.1%), preeclampsia without severe features 32 (15.46%), preeclapmsia with severe features 36 (17.39%), superimposed preeclampsia without severe features 2 (0.97%), superimposed preeclampsia with severe features 21 (10.14%), HELLP syndrome (1 (0.48%), eclampsia 1 (0.48%) |
| Perry 2018 [114] | Historical data | Significantly fewer patients developed PE in the HBPM group compared with the control group (20.4%, n=22 vs 34.5%, n=20,P =0.046) |
| Van den Heuvel 2020 [15] | Historical data | eHealth intervention increased the diagnosis of HDP.  Incidence of preeclampsia: Safe@home n=22 (21.4%), control 27 (20.3%) p=0.84, incidence of HELLP Safe@home 1 (1%), control 0 p=0.44, incidence of gestational hypertension Safe@home 9 (8.7%), control 4 (3%) p = 0.06 |
| Hoppe 2020 [75] | Conventional care (no eHealth) | In the eHealth arm severe hypertension occurred in 56 (26.2%) women and 116 (54.2%) had elevated BPs that required treatment after discharge (outcomes from conventional care not reported) |
| Marko 2016 [108] | Conventional care (no eHealth) | 1 patient developed preeclampsia during labour |
| Hirshberg 2023 [96] | Two comparator groups – Cohort A - same hospitals historical cohort (conventional care) and Cohort C - contemporaneous different hospitals conventional care | eHealth intervention compared to cohort A and cohort C – eclampsia (3 (0.3%) vs 6 (0.59%) vs. 8 (0.63%)), HELLP 4 (0.49%) vs. 7 (0.69%) vs. 11 (0.86%)) |
| Abelman [43] | Historical data - retrospective cohort - some telehealth used however not officially implemented | There was no difference between gestational age at diagnosis of HDP (37.5 weeks in non eHealth cohort compared to 37 weeks in eHealth cohort) |
| Burgess [68] | Historical data - cohort at 3 (of the 5 hospitals intervention utilised at) hospitals | 9% (n=107) patients entered at least one critical range BP (>/=150 mmHg systolic and or >/= 100 mmHg diastolic). Of these patients who entered a critical range BP, 26% (n=28) entered more than one critical range BP |
| Kalafat 2019 [107] | Usual care (no eHealth intervention) | 56 (70%) remote BP monitoring with app patients were diagnosed with gestational hypertension, 20 (25%) remote monitoring patients diagnosed with preeclampsia compared to 41 (65.1%) control patients diagnosed with gestational hypertension and 22 (34.9%) patients diagnosed with preeclampsia |
| Lanssens 2018 [91] | Usual care (no eHealth intervention) | The prevalence of gestational hypertension was higher in the eHealth group than in the conventional care group (60/86 [69.77%] versus 92/215 [42.79%], b = 0.24) p<0.01, but the prevalence of pre-eclampsia was lower in the RM group (17/86 [19.77%] versus 95/215 (44.19%], b = –0.23), p <0.01 |
| Lanssens 2017[83] | Usual care (no eHealth intervention) | For both uni- and multivariate analysis the prevalence of gestational hypertension higher in RM than in CC (81.25% vs 42.86% and beta=6.62), but the prevalence of preeclampsia was lower (14.85% vs 43.87% and beta=.24) |
| Mussarat 2022 [26] | Usual care (no eHealth intervention) | Rate of HDP with mobile health remote BP monitoring compared to control - Pregnancy associated hypertension n = 723, 22%, control n =1233 19%, aOR 1.22 (1.10, 1.36), gestational hypertension n=572 (18%) control n = 887 14$ aOR 1.35 (1.21, 1.52), preeclampsia without severe features n=107 3%, control n=253 (4%) aOR 0.84 (0.67, 1.06), preeclampsia with severe features n = 230 7% control n =398 6% aOR 1.17 (0.99, 1.38). Incidence of eclampsia: n=5 0.2%, control n=14 0.2% aOR 0.71 (0.26, 1.98), gestational hypertension n=572 (18%) control n = 887 14% aOR 1.35 (1.21, 1.52) |
| Nakahara 2021 [118] | Usual care (no eHealth intervention) | Incidence of eclampsia: eHealth n=1 (0.002%), control n=54 (0.02%) |
| Nuss 2021 [109] | Usual care (no eHealth intervention) | Superimposed preeclampsia remote BP monitoring n=33(5.4), control n=116 (7.6). Preeclampsia without severe features remote n=99 (16.2), control n=180 (11.8). Preeclampsia with severe features remote n=152 (24.9), control n=402 (26.5). All p value <0001. Incidence of eclampsia: Remote n=1 (0.2), control n=8 (0.5). Incidence of gestational hypertension: Remote n=255 (41.7), control n=596 (39.2) |
| Pealing 2019 [63] | Usual care (no eHealth intervention) | Rate of preeclampsia Chronic HTN SMBP 19/53 (36%), control 5/30 (17%) aOR 2.9. Gestational HTN SMBP 19/49 (39%), control 8/22 (36%) aOR 1.1 |
| Tucker 2022 [98] | Usual care (no eHealth intervention) | Maternal severe hypertension SM (self monitoring group) 69/1171 6%, usual care group 57/1175 (4.9%) p=0.25. Clinic hypertension recorded for 363 (15.5%) of those randomised, of whom 102 (4%) had preeclampsia - 179 (15.3%) in intervention group and 184 (15.7%) in usual care group . SM group 51/1209 (4.2%), usual care 51/1209 (4.2%) p >0.99 |
| Zhang [86] | No comparator | Diagnosis during program - preeclampsia incidence in fully adhere group n= 10 (47.62%), mostly adhere n=7 (20%), partially adhere n= 4 (19.05%), not adhere n=7 (33.33%), p=0.104. New gestational diagnosis fully adhere group n=3 (14.29%), mostly adhere n= 7 (20%), partially adhere n=3 (14.29%), not adhere n=4 (19.05%), p=0.949. Other hypertension fully adhere n=6 (28.57%), mostly adhere n=11 (31.43%), partially adhere n=8 (38.1%), not adhere n=7 (33.33%), p=0.925. |

Maternal and neonatal outcomes

| Study | Comparator | Outcome |
| --- | --- | --- |
| Participants at risk of HDP |  |  |
| Hackeloeer 2023 [106]. | No comparator | 20 developed an adverse outcome, 10 did not |
| Hacker 2022 [88]. | No comparator | 17 women were referred to the Emergency Department for evaluation of severely high blood pressure or mildly elevated blood pressure with symptoms of preeclampsia |
| Marko 2016 [108]. | No comparator | 1 patient developed IUGR |
| Zizzo 2021 [99]. | No comparator | Two fetuses died; one with major malformations and hydrops fetalis at GA 34+5 weeks, the other with severe FGR from week 20 and a birthweight of 635 g at GA 28+5bweeks. No severe maternal complications were observed. Amongst patients at high risk of preeclampsia (n=151) with remote BP monitoring with mobile device platform the incidence of preeclampsia was 21.2% (n = 32) |
| Perry 2018 [114] | Historical data | There was no difference in the number of adverse maternal, fetal or neonatal outcomes between the two groups |
| Van den Heuvel 2020 [15]. | Historical data | Incidence of SGA <5th cent. Safe @ home 5 (4.9%), control 12 (9%) p =0.22 |
| Mussarat 2022 [26]. | Conventional care (no eHealth) | eHealth compared to control - rates of preterm birth n=343 11%, control n=823 13% aOR 0.81 (0.71, 0.93), rate of SGA n=21 1%, control n=58, 1% aOR 0.72 (0.44, 1.19) |
| Tucker 2022 [98]. | Conventional care (no eHealth) | Rate of SGA SM group 104/1249 (8.3%), usual care 87/1235 (7%) p = 0.32. Rate of stillbirth Self monitoring group 5/1260 (0.4%), usual care group 3/1248 (0.2%). Neonatal death self monitoring 2/1248 (0.2%), usual care 0/1240 |
| Xydopoulos 2018 [113]. | Conventional care (no eHealth) | There was no difference in the number of adverse maternal, fetal or neonatal outcomes between the two groups |
| Zhang [86] | No comparator | Severe maternal morbidity rates – fully adhere n=7 (33.33%), mostly adhere n=15 (42.86%), partially adhere n=6 (28.57%), not adhere n=9 (42.86%) |
|  |  |  |
| Participants with HDP |  |  |
| Denolle 2008[46]. | No comparator | The duration of gestation and the type of delivery was comparable in the two groups (38 ± 1 vs. 37 ± 8 weeks and one vs three caesarean deliveries) as was the Apgar score (9.8 ± 0.7 vs. 9.7 ± 0.8 ) |
| Hirshberg 2023 [96]. | Two comparator groups – Cohort A - same hospitals historical cohort (conventional care) and Cohort C - contemporaneous different hospitals conventional care | Significantly fewer eHealth participants had any adverse clinical outcomes relative to control comparator A without eHealth (2.9% vs 4.7%; OR 0.61, 95% CI 0.40–0.98, adjusted OR 2.3% vs 4.5%; OR 0.54, 95% CI 0.33–0.87) and no significant difference relative to control comparator C (3.2% vs 4.5%; OR 0.71, 95% CI 0.47–1.07, adjusted OR 2.9% vs 4.9%; OR 0.59, 95% CI 0.40–0.88) |
| Lopez 2018 [78]. | Usual care (no eHealth intervention) | The number of patients with controlled BP was increased by 11% and the rate of maternal deaths was reduced by 7% |
| Patel 2023 [97]. | No comparator | Rate of preterm birth: 45 (22.06%). Rate of foetal growth restriction (undefined): 18 (8.7%) |
| Scalise 2015 [65]. | No comparator | Two of the 11 phone contacts required escalation in care and referral to an acute care centre |
| Spiro 2023 [94]. | No comparator | Of 450 recorded BP measurements, 88 (19.6%) were mild and 2 (0.4%) were severe |
| Denolle 2008[46] | Same eHealth intervention – clinician aware (eHealth cohort) or blinded to remote BP results (control) | There was no significant difference between the home based BP monitoring (HBPT) and transmission of data versus storage of data - control group (CM) (HBPT and CM) for clinical outcomes: number of hospitalizations during pregnancy (2 vs 1), duration of hospitalization after delivery (5.7 ± 1.1 vs. 5.9 ± 1.2 days), sick leave days during pregnancy (8.1 ± 18.1 vs. 6.1 ± 8.6 days), the duration of gestation and the type of delivery was comparable in the two groups (38 ± 1 vs. 37 ± 8 weeks and one vs three caesarean deliveries) as was the Apgar score (9.8 ± 0.7 vs. 9.7 ± 0.8 ) |
| Chappell 2022 [104] | Conventional care (no eHealth) | Comparing remote BP monitoring (self monitoring BP – SMBP) with telemonitoring system using an app compared to usual care (attending an antenatal clinic) there was no significant difference in the majority of maternal and infant outcomes, other than a lower proportion with spontaneous onset of labor: 12 participants (5%) in the SMBP group vs 21 participants (10%) in the usual care group (adjusted odds ratio, 0.52 [95% CI, 0.29 to 0.92]) |
| Kalafat 2019 [107]. | Conventional care (no eHealth) | No significant differences were observed regarding the gestational age at delivery (P=0.064). The incidence of preterm birth prior to 34 weeks was similar between the two groups (P=0.582). The incidence of vaginal delivery, operative delivery and elective cesarean section were similar between the HBPM and control groups (P=0.171). No significant differences were observed regarding maternal high-dependency unit admission (P=0.999), birth weight centile (0.803), fetal growth restriction (p=0.999), neonatal intensive care unit admissions (p=0.507) and composite neonatal (p=0.654), maternal (p=0.999) or fetal adverse outcomes (p=0.999) |
| Lanssens 2016 [95]. | Conventional care (no eHealth) | Maternal and neonatal outcomes were not different for gestational age at delivery, birthweight, birthweight percentile, length, Apgar at 1 min or at 5 min and pH arterial or venous. In RM versus RC, the number of spontaneous deliveries is higher (47% versus 27%;p<0.01) and the number of admissions to the NIC was lower (11.3% versus 9.2%;p<0.02) |
| Pealing 2019 [63]. | Conventional care (no eHealth) | Maternal and perinatal outcomes for women with chronic and gestational hypertension were also similar between groups Preterm birth <34 weeks. Chronic HTN SMBP 7/53) 13%, control 2/30 (7%). Gestational HTN SMBP 2/49 (4%), control 2/22 (9%). Birth weight <10th cent. Chronic HTN SMBP 8/53 (15%) control 1/30 (3%) aOR 5.3, Gestational HTN SMBP 9/49 (16%) control 4/22 (18%). Chronic HTN SMBP 2/53 (4%) control 0/30 (0%), Gestational HTN SMBP 0/49 (0%) control 0/22 (0%). Neonatal death Chronic HTN SMBP 1/53 (2%) control 0/30 (0%), Gestational HTN SMBP 0/49 (0%) control 0/22 (0%) |
| Lanssens 2017[83]. | Conventional care (no eHealth) | Neonates in the RM group, compared with CC group, were less likely to be admitted to the NIC department when performed a univariate analyses (10.42%, 5/48 vs 27.55%, 27/98) but not in multivariate analyses (beta=.34) |
| Kitt 2023 [84] | Conventional care (no eHealth) | There were 2 readmissions per group (eHealth and control) for serious adverse events (neonatal jaundice, maternal sepsis, maternal wound infection, sepsis). Between both groups there were similar rates of breastfeeding (54.8% in intervention group vs. 43.9% in control group, no statistical difference) and hormonal contraception (23.1% progesterone-only pill in intervention group vs. 20.4% in the control group, and 3.8% vs. 4.1% for combined-pill, respectively). |
| Kumar [100] | Historical data - cohort prior to telehealth visits (face to face) | The eHealth intervention improved non-Black patients rates of long acting reversible contraception initiation, Papanicolau test completion, postpartum depression screening participation, and cardiology follow up visit attendance |
|  |  |  |
| Case reports |  |  |
| Benczur [117] | No comparator | Case report – remote BP monitoring prompted patient to check BP which was elevated, she was medicated with antihypertensives and delivered preterm at 30 weeks via caesarean section |
| Cheu [73]. | No comparator | Case report – remote BP monitoring prompted patient to check BP which was elevated, she was medicated with antihypertensives and underwent induction of labour |
| Sheth 2019 [111]. | No comparator | Patient 1 - postpartum BPs were elevated patietn was admitted for magnesium sulfate. Patient 2 postpartum BP elevated - led to hospital admission for magnesium sulfate. 15 BPs were recorded after discharge and antihypertensives titrated according to these data points |

Medication adjustment

| Study | eHealth intervention | Outcome |
| --- | --- | --- |
| Boggess 2020 [50] Janssen 2021 [62]. | Remote BP monitoring with transmission of data via web based platform and text message communication | 31 (16%) were started on oral antihypertensive treatment |
| Deshpande 2021[53]. | Remote BP monitoring with text message communication | During follow-up, antihypertensive doses were increased in three women |
| Hirshberg 2019 [60]. | Remote BP monitoring with text message communication and data storage in web based platform | There was no difference in percentage of participants who required new antihypertensive medication or dose escalation in the eHealth and usual care cohort (19% text vs 21% office; P=0.73) |
| Hoppe 2019 [74]. | Remote BP monitoring with tablet device and blood pressure cuff (Bluetooth transmission of data) with telehealth review | 14 (25%) of participants were prescribed an increased dose of an anti-hypertensive medication and 11 (20%) were started on their first antihypertensive |
| Pealing 2019 [63]. | Remote BP monitoring with data submission via text message or smart phone application with web based platform data storage | No differences were seen in mean defined daily dose, nor the mean number of antihypertensive medications between the eHealth self monitoring group versus usual care |
| Spiro 2023 [94]. | Remote BP monitoring with automatic uploading of data to a web based platform with communication via mobile phone application and telehealth | Medication up-titration occurred seven times in six participants (20%) |
| Triebwasser 2020 [42]. | Remote BP monitoring with text message communication and data storage on web based platform | 28/43 (65.1%) had persistently elevated BP requiring medication initiation (n = 12), titration (n = 6), or both (n = 10). A further 15 women had medication initiated (n = 8), titrated (n = 4), or both (n = 3) due to persistent postpartum hypertension. Medication initiation or titration through the texting program did not differ in the eHealth cohorts (trial versus implementation of the eHealth over different time periods - 13.2% vs. 16.5%, p = 0.4 |
| Kitt 2023 [84] | Remote blood pressure monitoring Bluetooth BP cuff with communication with clinician via mobile phone app, storage of data on web based platform | Similar antihypertensive classes were prescribed in each group. At visit 2 (week 1) more antihypertensives were prescribed (defined daily doses, 1.5 vs 0.7, p=0.01) in the intervention group than in the control group |
| Lemon [70] | Remote BP monitoring with text message communication with data storage on web based platform and telehealth review | Patients enrolled in the  remote monitoring program were more likely to  be initiated on a new antihypertensive  postpartum (24.4% vs 5.4%, P,.001), 20 more patients were initiated on an antihypertensive  medication (propensity score–matched aRD 20.0, 95% CI, 18.4–21.5) for every 100 engaged in the program |
| Lewkowitz [45] | Remote BP monitoring with Bluetooth BP cuff with data transmission via mobile phone application utilising AI to respond to BPs or symptoms with communication with clinicians via email | Manual BP and Automatic BP patients were equally  likely to have BP medications initiated/titrated remotely |

Actual BP

| Study | Comparator | Outcome |
| --- | --- | --- |
| Lower BP with eHealth intervention |  |  |
| Cairns 2018 [51]. | Conventional care (no eHealth) | The adjusted difference in the mean BP readings was greatest at 6 weeks postpartum: intervention group mean (SD), systolic 121.6 (8.7)/diastolic 80.5 (6.6) mm Hg; control group, systolic 126.6 (11.0)/diastolic 86.0 (9.7) mm Hg; adjusted differences, systolic −5.2 (95% CI, −9.3 to −1.2) /diastolic −5.8 (95% CI, −9.1 to −2.5) mm Hg. Mean DBP was consistently lower in the intervention group from 4 weeks to 6 months. Participants in the intervention group were more likely to have SBP and DBP readings inside the target range at 6 weeks postpartum: adjusted odds ratio (OR) 8.0 (95% CI, 2.1–30.6) |
| Khosla 2022[90]. | Historical data | At the first postpartum clinic visit, the mean diastolic BP was higher in the post-telehealth period (85 mm Hg [80−91]) than pretelehealth (78 mm Hg [71−85]; P<.0001) |
| Kitt 2021 [24]. | Conventional care (no eHealth) | Women who self-managed their BP postpartum had a lower 24-hour diastolic BP of 73.7±5.0 mm Hg compared with 80.7±7.4 mm Hg in the usual care group adjusted mean difference, −7.4 mm Hg [95% CI, −10.7 to −4.2]; *P*<0.001. The difference in 24-hour diastolic BP between the cohorts was maintained when adjusting for baseline diastolic BP (adjusted mean difference, −7.3 mm Hg [95% CI, −10.5 to −4.0]; *P*<0.001) or the antenatal diastolic BP (adjusted mean difference, −6.9 mm Hg [95% CI, −10.3 to −3.6]; *P*<0.001. Diurnal diastolic BP was 5.3 mm Hg lower in the selfmanagement group ([95% CI, −8.6 to −2.0] *P*=0.002), and both nocturnal diastolic and systolic BP were significantly lower in the self-management group (adjusted mean difference: nocturnal diastolic BP, −7.8 mm Hg [95% CI, −11.9 to −3.7]; *P*<0.001 and nocturnal systolic BP, −7.4 [95% CI, −14.2 to −0.7]; *P*=0.032). Twentyfour– hour and diurnal systolic BP did not significantly differ |
| Kitt 2023 [84] | Conventional care (no eHealth) | 40% had gestational hypertension, and 60% had preeclampsia. Two hundred participants (91%) were included in the primary analysis. The 24-hour mean (SD) diastolic blood pressure, measured at 249 (16) days postpartum, was 5.8 mm Hg lower in the intervention group (71.2 [5.6] mm Hg) than in the control group (76.6 [5.7] mm Hg). The between-group difference was -5.80 mm Hg (95% CI, -7.40 to -4.20; P < .001). Similarly, the 24-hour mean (SD) systolic blood pressure was 6.5 mm Hg lower in the intervention group (114.0 [7.7] mm Hg) than in the control group (120.3 [9.1] mm Hg). The between-group difference was -6.51 mm Hg (95% CI, -8.80 to -4.22; P < .001) |
| Higher BP with eHealth intervention |  |  |
| Pealing 2019 [63]. | Conventional care (no eHealth) | Diastolic BP was higher in the self-monitoring group compared to usual care (mean and time-weighted mean clinic diastolic BP) |
| No difference in blood pressure between eHealth and control |  |  |
| Chappell 2022 [104]. | Conventional care (no eHealth) | No difference in mean systolic BP |
| No comparison |  |  |
| Hackeloeer 2023 [106]. | No comparator | There was no significant difference in the systolic blood pressure (p=0.47). The mean arterial pressure (MAP) was significantly different in the period 0 to -50 (p=0.036) days before delivery, but not significantly distinct in the period between -50 to -100 days before delivery (p=0.055) |
| Runkle 2021 [110]. | No comparator | Home systolic and diastolic BP readings varied from clinic readings. The difference did not exceed >2 SDs above the mean for clinic visits and suggests that prenatal home BP monitoring may be used in early detection of anomalous BP readings. Remote monitoring can detect elevated BP earlier than in routine clinic visits |
| Duncan [38] | No comparator | 34.9 % of all patients had a diagnosis of preeclampsia. Rates of stage 2 hypertension (BP >140/90) decreased from 42.2 % to 14.3 % from week 1 to week 6. When stratified by race, 22.4 % of Black patients had stage 2 hypertension  at 6 weeks compared to 2.2 % of non-Black patients (p<0.0001). |
| Lemon [70] | Conventional care (no eHealth) | Trajectories demonstrate  a marked increase in systolic BP at 1 week postpartum that importantly returns to prenatal values by 6 weeks postpartum for patients enrolled in the  program (121.1 mm Hg vs 120.6 mm Hg; paired t test P5.045). Trajectories of diastolic BP show a similar spike at 7 days, though they did not return entirely to prenatal measurements by 6 weeks postpartum (75.5 mm Hg vs 78.6 mmHg; paired t test P,.001) |
| Mujic [71] | No comparator | The majority of patients (94.2%) had at least 2 BP measurements above the <120/ and <80 mm Hg range in the 6-week postpartum  period, with 62.3% reporting ≥2 readings of ≥140/  or 90 mm Hg. The highest prevalence of BP ≥140/  or >90 mm Hg was observed among non-Hispanic  Black patients (71.9%). We examined the ranges of BP  in the early and late postpartum periods. Compared with the early postpartum period, the prevalence of ≥140/or >90 mm Hg in the late postpartum period decreased in all subgroups, but differences by race and ethnicity persisted. |

Cost analysis

| Study | Comparator | Outcome |
| --- | --- | --- |
| Eedarapalli 2019[54] | Historical data | Antenatal day assessment Unit’s workload reduced by 2.3%. Costs per patient on Flo were £80. The net savings were £36.7K for the year on Flo |
| Van den Heuvel 2021 [35]. | Historical data | In the SAFE@HOME group, a significant cost reduction for direct health care costs of 19.7% or €888 (€3616 vs 4504, p = 0.001) was found. For each euro associated with costs of the digital platform, an average of €7.7 was saved for antenatal care resources. In the additional analysis, costs from a societal perspective were added to direct health care costs. Both travel costs (€245 vs. €280, p *<* 0.001) and loss of productivity costs (€3565 vs €4329, p *<* 0.001) were lower for the SAFE@HOME group. Combined cost calculations of antenatal care resulted in total savings in healthcare costs and societal costs of 18.2% or €1665 (€7485 vs €9150, p *<* 0.001). When comparing means of total healthcare costs, a difference was found of € 1338 or 18.7% (€5805 vs €7143). When adding costs of travelling and work absence (societal costs), costs decreased from €9150 in usual care to €7485 with use of the digital platform (median cost difference €1665 or 18.2%, p *<* 0.001) (*mean* cost difference €2174) |
| Campbell [37] | Conventional care (no eHealth) | In BUMP 1, mean (SE) total costs with SMBP and with usual care were £7200 (£323) and £7063 (£245),  respectively, mean difference (95% CI), £151 (−£633 to £936). For the BUMP 2 chronic hypertension cohort, corresponding  figures were £13 384 (£1230), £12 614 (£1081), mean difference £323 (−£2904 to £3549) and for the gestational  hypertension cohort were £11 456 (£901), £11 145 (£959), mean difference £41 (−£2486 to £2567). The per-person  cost of telemonitoring was £6 in BUMP 1 and £29 in BUMP 2. SMBP was not associated with changes in the cost of health care contacts for individuals at risk of, or with, pregnancy hypertension |
| Denolle 2008[46]. | Same eHealth intervention – clinician aware (eHealth cohort) or blinded to remote BP results (control) | No cost saving for HBP group |
| Lanssens 2018 [25] | Conventional care (no eHealth) | Reductions in the number of ED visits and hospital readmissions led to significantly lower total medical costs in the first 6 months after delivery for the program group compared with cohort A ($32.20 PMPM, 95% CI $24.90–39.50) and cohort C ($29.40 PMPM, 95% CI $25.90–32.90). Cost analysis of ehealth vs. control - no difference in cost for prenatal follow up, remote monitoring had 34.51% less Belgian national health care system (HCS) costs and 41.72% less National Institution for Insurance of Disease and Disability (RIZIV) costs for lab test results, a reduction of 47.16% in HCS and 48.19% in RIZIV costs for neonatal care. HCS costs for medication were 1.92% lower in RM than conventional care but were 0.69% higher for RIZIV. Overall HCS costs for remote monitoring were mean 4233.31 euros per person and 4873.69 euro per person for conventional care (p=0.82), a reduction of 740.38 euro per person |
| Xydopoulos 2018 [113]. | Conventional care (no eHealth) | The mean saving per week for the total HBPM group compared with the control group was £200.69, while the average saving per week for the App-HBPM cohort compared with the control group was £286.53 |

Healthcare utilisation

Number of face-to-face visits/ health care service utilisation

| Study | Comparator | Outcome |
| --- | --- | --- |
| Reduction in healthcare service utilisation with eHealth intervention |  |  |
| Antenatal |  |  |
| Perry 2018 [114] | Usual care (no eHealth intervention) | Number of antenatal outpatient visits overall for  hypertension per patient - eHealth 6.5 (4.0–9.0), conventional care 8.0 (6.0–10.3) p= 0*.*003 |
| Fazal 2020[55]. | No comparator | More than 800 healthcare appointments were avoided during the 12 months |
| Lanssens 2016 [95]. | Usual care (no eHealth intervention) | There is no difference between the two groups in the total number of prenatal visits, CTG’s and echo’s during pregnancy, or amount of days admitted to the MIC. In RM versus RC, the total number of MIC admissions(29% versus 74%;p50.01) and the number of long-stay birthing admissions to the MIC (18% versus 64%;p50.01) were lower |
| Perry 2018 [114]. | Historical data | Patients with remote BP monitoring had significantly fewer visits per patient to the day assessment unit compared to usual care (4 vs 6, P <0.001), as well as reduced visits to other antenatal services for blood pressure related visits (6.5 vs 8.0, P =0.003) |
| Van den Heuvel 2020 [15]. | Historical data | The number of antenatal visits from the first visit to delivery was significantly lower in the SAFE@HOME group as compared to the control group (mean 13.7 [4.1] vs 16.0 [4.0], p < 0.001). The total number of ultrasound assessments was also significantly lower in the SAFE@HOME group (mean 6.3 [2.7] vs 7.4 [2.9], p = 0.005). In the SAFE@HOME group, observational admissions for hypertension or diagnosis/exclusion of suspected preeclampsia were significantly lower compared to the control group (2.9% vs 13.5% of participants, p = 0.004. |
| Postnatal |  |  |
| Saghir 2015 [92]. | Historical data | There was a 65% reduction in community midwives visit, 75% reduction in patient travel and no readmissions |
|  |  |  |
| No difference in healthcare service utilisation with eHealth intervention |  |  |
| Antenatal |  |  |
| Denolle 2008[46]. | Same eHealth intervention – clinician aware (eHealth cohort) or blinded to remote BP results (control) | There was no significant difference between the two groups (HBPT and CM) for any criteria: number of obstetrician and nurse visits (0.8 ± 1 and 2.3 ± 2 vs. 1.6 ± 1.3 and 1.7 ± 2.2 in control and HBPT groups, respectively), total time spent travelling from home to the obstetrical center (61.1 ± 23.2 vs. 49.2 ± 16.6 minutes), number of hospitalizations during pregnancy (2 vs 1), duration of hospitalization after delivery (5.7 ± 1.1 vs. 5.9 ± 1.2 days), sick leave days during pregnancy (8.1 ± 18.1 vs. 6.1 ± 8.6 days), and number of obstetrical ultrasound scans (1 ± 1 vs. 1.3 ± 1.2) |
| Hoppe 2020 [75]. | Usual care (no eHealth intervention) | There were no differences between the groups in the number of ER or triage visits or in the use of antihypertensives 6 weeks postpartum |
|  |  |  |
| Mixed results in healthcare service utilisation |  |  |
| Antenatal |  |  |
| Kalafat 2019 [107]. | Conventional care (no eHealth) | HBPM pathway significantly reduced the number of DAU visits (median 4.0 vs. 5.0, P=0.009). No difference was observed between the groups regarding the total number of outpatient (P=0.357) and triage visits (p=0.237). However, the total number of antenatal visits adjusted for the duration of monitoring was significantly lower for the HBPM group compared to controls (median 1.4 vs 1.8, P=0.020) |
| Xydopoulos 2018 [113]. | Conventional care (no eHealth) | Women in the App-HBPM cohort visited the DAU significantly fewer times over the course of the monitoring compared with women in the non-App HBPM and control groups (median (IQR); 1 (0–3) vs 5 (2–7) and 6 (5–8), respectively, P <0.001); however, they attended the hypertension clinic significantly more times than did the other two groups (P <0.001) |
|  |  |  |
| Increase in healthcare utilisation with eHealth intervention |  |  |
| Postnatal |  |  |
| Rhoads 2016 [79]. | Conventional care (no eHealth) | There was a significant difference between users and nonusers ( p = 0.0046), where 42.9% (n = 9) of the users returned to a medical facility, while none of the nonusers returned to a medical facility |
| Hirshberg 2023 [96]. | Historical data | Program participants had more postdelivery specialist visits |
|  |  |  |
| Observations on healthcare service utilisation with no comparison |  |  |
| Postnatal |  |  |
| Goodin 2023[105]. | No comparator | There were 1536 assessments completed in the app for hypertension, there were 39 positive screenings. The assessment based triggers from positive screens resulted in 209 nurse follow ups, 35 face to face visits and 8 hospital admissions |
| Hauspurg 2019[57]. | Historical data | 177 (43%) women did not require the previously scheduled in-office blood pressure check at 1-week postpartum |
