# Supplementary material for: eHealth and Hypertensive Disorders of Pregnancy: Systematic Review
Source: J Med Internet Res. 2025 Sep 10;27:e77064. doi: 10.2196/77064 (PMC12422594; doi:10.2196/77064)
Supplement: Multimedia Appendix 4 [file jmir-v27-e77064-s004.docx]

Appendix 4 – Study summary

Abbate poster [47]

| Methods | Study design: retrospective cohort study  Study duration: 12 months (9/2018-8/2019)  Study follow-up: 10 days |
| --- | --- |
| Participants | Country: USA  Setting: Hospital  Number: 877  Education level: not reported  Mean age (years): not reported  Inclusion criteria: women diagnosed with chronic hypertension (HTN) or a hypertensive disorder of pregnancy (HDP) at the time of delivery discharge  Exclusion criteria: not reported |
| Interventions | Intervention type classification: self-monitoring, decision aid (communication with clinician)  Intervention group: remote blood pressure (BP) monitoring program with text message communication  Control group: nil |
| Outcomes | Patterns in postpartum hospital visits and readmission rates |
| Notes | No other publications for this study identified  Funding source: none reported |

Ackerman poster [72]

| Methods | Study design: matched cohort study  Study duration: not reported  Study follow-up: 10 days |
| --- | --- |
| Participants | Country: USA  Setting: Hospital  Number: 172 women with chronic HTN or HDP (31 intervention and 141matched controls)  Education level: not reported  Mean age (years): not reported, maternal age >35 years (number) 10, control 47  Inclusion criteria: postpartum patients with peripartum HDP  Exclusion criteria: not described |
| Interventions | Intervention type classification: self-monitoring, decision aid (communication with clinician)  Intervention group: remote BP monitoring with Bluetooth BP cuff and telehealth clinician review  Control group: routine care |
| Outcomes | Primary outcome: outpatient adherence -a documented interaction with a provider within 10 days of delivery hospitalization discharge |
| Notes | No other publications for this study identified  Funding source: non described |

Arkerson [48]

| Methods | Study design: Randomised Controlled Trial (RCT)  Study duration: 6 months (April- September 2021)  Study follow-up: 16 days |
| --- | --- |
| Participants | Country: USA  Setting: Two academic training institutions (hospitals)  Number: 202 randomised, 197 patients in final analysis (intervention 96 control 101)  Education level: not reported  Mean age (years): intervention 28.9 control 29.9  Inclusion criteria: postpartum and were diagnosed with gestational hypertension, preeclampsia, or chronic hypertension with superimposed preeclampsia in the antenatal or immediate postpartum period of the delivery admission.  Exclusion criteria: younger than age 18 years, did not speak English, had body mass indexes (BMI) of 50 or higher, or did not have a phone with unlimited texting capability or the ability to receive calls. |
| Interventions | Intervention type classification: self-monitoring, decision aid (communication with clinician)  Intervention group: remote blood pressure monitoring with communication via smartphone application (app) with text message communication with clinician  Control group: in-person outpatient blood pressure check within 10 days of discharge |
| Outcomes | Primary outcome: the ascertainment of any blood pressure within 10 days postpartum  Secondary outcomes: rates of initiation or up titration of antihypertensive medications, unscheduled outpatient  or obstetric triage visits for hypertension, readmission for hypertension, and attending standard postpartum follow-up 6–8 weeks after delivery |
| Notes | No other publications for this study identified  Funding source: non described |

Benczur poster [117]

| Methods | Study design: case report  Study duration: 19 weeks  Study follow-up: 19 weeks |
| --- | --- |
| Participants | Country: Hungary  Setting: outpatient and hospital  Education level: not reported  Age: 29 years  Number: 1  Inclusion criteria: not described  Exclusion criteria: not described |
| Interventions | Intervention type classification: self-monitoring, decision aid (communication with clinician)  Intervention group: Tensiocare System – BP monitor with built-in memory and automated data-transfer capabilities over telephone network  Control group: nil |
| Outcomes | Description of patient care and clinical outcome |
| Notes | No other publications for this study identified  Funding source: non described |

Binstock poster [49]

| Methods | Study design: retrospective cohort study  Study duration: 12 months (January-December 2018)  Study follow-up: 56 days |
| --- | --- |
| Participants | Country: USA  Setting: Tertiary hospital  Number: 340  Education level: not reported  Mean age (years): intervention 31.1, control 30.5  Inclusion criteria: women with HDP admitted to the postpartum unit of a single tertiary care hospital.  Exclusion criteria: not reported |
| Interventions | Intervention type classification: self-monitoring, decision aid (communication with clinician)  Intervention group: remote blood pressure monitoring with text message communication  Control group: a historical cohort of women with HDP six months prior to the onset of program enrolment |
| Outcomes | Primary outcome: healthcare utilisation rate, follow up rate, readmission rate |
| Notes | No other publications for this study identified  Funding source: non described |

Bisson [87]

| Methods | Study design: prospective cohort study  Study duration: 6 months (April-September 2021)  Study follow-up: not reported |
| --- | --- |
| Participants | Country: USA  Setting: Multiple sites (179 hospitals, clinics, and perinatal quality collaborative centres)  Number: 2910 Cuff Kits were distributed to individuals in 179 different institutions across 14 states, with 117 patients completing the survey (response rate 4 %)  Education level: not reported  Mean age (years): not reported  Inclusion criteria: patients were included based on their risk factors for preeclampsia development as assessed by their provider.  Exclusion criteria: nil |
| Interventions | Intervention type classification: self-monitoring, decision aid (communication with clinician)  Intervention group: remote BP monitoring with Cuff Kits which include home blood pressure monitoring (HBPM) devices, patient information about preeclampsia and hypertension in pregnancy, and patient response postcards and telehealth communication with clinician  Control group: nil |
| Outcomes | Primary outcome: provider’s perception of HBPM utilization by patients and the impact of home blood pressure monitoring on their patient care.  Secondary outcome: patient perception of home blood pressure monitoring assessed through survey responses. |
| Notes | No other publications for this study identified  Funding source: The research was funded by the Preeclampsia Foundation |

Boggess poster [50] + Janssen [62]

| Methods | Study design: prospective cohort study  Study duration: not reported  Study follow-up: 10 days |
| --- | --- |
| Participants | Country: USA  Setting: 3 sites (large academic medical centres)  Number: total 199 participants were enrolled: 66 at site A (88 eligible), 67 at site B (66 eligible), and 66 at site C (112 eligible).  Education level: not reported  Mean age (years): all patients 31.2, Site A 31, Site B 32, Site C 31  Inclusion criteria: English speaking postpartum patients with a diagnosis of HDP, defined as chronic hypertension with 1 or more of the following: preeclampsia, gestational hypertension, preeclampsia with severe  features, or haemolysis, elevated liver  enzymes, low platelet count syndrome. The first 66 eligible participants who agreed to participate in the program at each site, were enrolled.  Exclusion criteria: nil |
| Interventions | Intervention type classification: self-monitoring, decision aid (communication with clinician)  Intervention group: remote BP monitoring with transmission of data via web based platform and text message communication, interaction with clinicians via telehealth  Control group: nil |
| Outcomes | Primary outcome: user engagement, defined as the submission of at least 1 BP measurement via text message through the program in the 10 days immediately following discharge. The total number of responses of BP readings and the number of responses received between 3 and 4 days postpartum and between 7 and 10 days postpartum, |
| Notes | Boggess poster [50] + Janssen [62]  Funding source: The research was funded by the Preeclampsia Foundation |

Burgess poster [102] + study [103]

| Methods | Study design: retrospective cohort  Study duration: 8 months (October 2019 – May 2019)  Study follow-up: 7 days |
| --- | --- |
| Participants | Country: USA  Setting: Hospital  Number: 54  Education level: not reported  Mean age (years): 28.2  Inclusion criteria: any woman who delivered at WellSpan York Hospital between October 2018 and May 2019 who experienced preeclampsia, received care from aWellSpanHealth obstetric care provider, and was enrolled or willing to enroll in MyWellSpan, the entity-specific patient portal, English-speaking  Exclusion criteria: not reported |
| Interventions | Intervention type classification: self-monitoring, decision aid (communication with clinician)  Intervention group: remote BP monitoring with MyWellSpan mobile app linked with web based platform with communication with clinicians  Control group: nil |
| Outcomes | Primary outcome: adherence – checking and reporting BP, ease of use |
| Notes | No other publications for this study identified  Funding source: non described |

Cairns [51] – SNAP-HT trial

| Methods | Study design: prospective, randomised, unmasked trial  Study duration: 13 months (April 1, 2015-April 30, 2016)  Study follow-up: 6 months |
| --- | --- |
| Participants | Country: United Kingdom (UK)  Setting: multiple sites (5 National Health Service (NHS) hospitals)  Number: 101 consented, 91 randomised, 82 in final analysis (intervention 40, control 42)  Education level: not reported  Mean age (years): intervention 31.7, control 31.7  Inclusion criteria: women aged ≥18 years, with gestational hypertension or preeclampsia requiring antihypertensive  treatment  Exclusion criteria: prescription of >3  antihypertensive medications, self-report of hypertension diagnosed outside of pregnancy, and inability to speak English |
| Interventions | Intervention type classification: self-monitoring, decision aid (communication with clinician)  Intervention group: self- management  entailed daily self-monitoring of BP with communication via text message or smartphone app with clinician  Control group: usual care (usual care patients had their BP monitored by their  community midwife and their antihypertensive medication adjusted  by their general practitioner) |
| Outcomes | Primary outcome: feasibility, recruitment, retention, and compliance with follow-up rates  Secondary outcomes: mean systolic BP, diastolic BP, and mean arterial pressure, postnatal readmission rates, safety data, side effects, quality of life scores (EQ-5D-5 L), compliance with and accuracy of remote BPM reporting |
| Notes | Prospective long term follow up of this trial published in Kitt [24]  Funding source: The research was funded by the National Institute for Health  Research (NIHR) Collaboration for Leadership in Applied Health Research and Care Oxford at Oxford Health NHS Foundation Trust and via a Research Professorship awarded to R.J. McManus  (NIHR-RP-02-12-015) |

Chappell [104] – BUMP 2 trial

| Methods | Study design: non blinded RCT  Study duration: 10 months (November 2018 - September 2019)  Study follow-up: up to 33 weeks (follow up from recruitment at booking in (earliest gestation 15 weeks) to 8 weeks after birth) |
| --- | --- |
| Participants | Country: UK  Setting: 15 maternity units  Number: 850 (intervention 430 control 420 control), primary outcome available for 416 (intervention) 405 (control)  Education level: not reported  Mean age (years): chronic hypertension SMBP 36 control 35.5, gestational hypertension SMBP 33.5, control 33.6  Inclusion criteria: Individuals aged 18 years or older with chronic hypertension (defined as sustained systolic BP ≥140 mm Hg and/or diastolic BP ≥90 mm Hg, present at booking or before 20 weeks’ gestation, or receiving antihypertensive treatment outside pregnancy or at time of referral) and were recruited up to 37+0 weeks’ gestation, or gestational hypertension (defined as sustained systolic BP ≥140 mm Hg and/or diastolic BP ≥90 mm Hg after 20 weeks’ gestation) and were recruited at 20 to 37 weeks’ gestation  Exclusion criteria: individuals considered likely to deliver within 48 hours of eligibility assessment |
| Interventions | Intervention type classification: self-monitoring, decision aid (communication with clinician)  Intervention group: self remote BP monitoring with communication via a telemonitoring system, an app and web based platform  Control group: usual prenatal care entailed pregnant individuals attending antenatal clinics as required, including BP measurement and, if needed, medication initiated or adjusted by their usual antenatal care team. Individuals randomized to usual care were not prevented from self-monitoring but did not receive the app or other advice regarding this |
| Outcomes | Primary outcome: mean systolic BP  Secondary outcomes: maternal and infant secondary outcomes |
| Notes | No other publications for this study identified  Funding source: This work was funded from a NIHR Programme Grant for Applied Research grant for applied research (RP-PG-1209-10051) and NIHR Professorships awarded to Dr McManus (NIHR-RP-R2-12-015) and Dr Chappell (NIHR -RP-2014-05-019). Drs McManus and Tucker received funding from the NIHR Collaboration for Leadership in Applied Health Research (CLAHRC) now recommissioned as NIHR Applied Research Collaboration Oxford and Thames Valley (ARC-OxTV). Dr Mackillop received support from the NIHR Oxford Biomedical Research Centre. Dr Hinton is based in The Healthcare Improvement Studies Institute (THIS Institute), University of Cambridge; THIS Institute is supported by the Health Foundation, an independent charity in the UK. Dr Sandall was supported by the NIHR Collaboration for Leadership in Applied Health Research and Care South London (NIHR CLAHRC South London) at King’s College Hospital NHS Foundation Trust, now recommissioned as the NIHR Applied Research Collaboration South London. Dr Yu’s research program is partly supported by NIHR Applied Research Collaboration (ARC)–West, NIHR Health Protection Research Unit (HPRU) for Behavioural Science and Evaluation, and the NIHR Southampton Biomedical Research Centre (BRC). Drs Chappell, McManus, Sandall, and Yu are NIHR senior investigators. Service support costs were administered through the NIHR Clinical Research Network. |

Cheu [73] letter to editor

| Methods | Study design: case report  Study duration: not reported  Study follow-up: 36 weeks |
| --- | --- |
| Participants | Country: USA  Setting: Hospital  Number: 1  Education level: not reported  Mean age (years): 30  Inclusion criteria: not reported  Exclusion criteria: not reported |
| Interventions | Intervention type classification: self-monitoring, decision aid (communication with clinician)  Intervention group: remote blood pressure monitoring with Bluetooth BP cuff, mobile phone app with communication with clinician  Control group: nil |
| Outcomes | Clinical outcome |
| Notes | No other publications for this study identified  Funding source: none reported |

Countouris [52]

| Methods | Study design: cohort study  Study duration: 17 months (December 2019-April 2021)  Study follow-up: 1 year |
| --- | --- |
| Participants | Country: USA  Setting: Hospital  Number: 140 patients (175 visits)  Education level: not reported  Mean age (years): virtual visit 33.6 in person visit (control) 34.6  Inclusion criteria: not reported  Exclusion criteria: not reported |
| Interventions | Intervention type classification: decision aid  Intervention group: virtual postpartum hypertension clinic review  Control group: usual care (face to face review in postpartum hypertension clinic) |
| Outcomes | Primary outcome: attendance at postpartum visits, feasibility of utilising telehealth for postpartum hypertension clinic |
| Notes | No other publications for this study identified  Funding source: This study was funded by the Magee Womens Hospital Medical Staff Grant. Dr. Hauspurg was funded by  the NIH/ORWH Building Interdisciplinary Research Careers in Women’s Health (BIRCWH) NIH K12HD043441. Dr. Countouris was funded by the NIH NHLBI T32 Training Grant HL129964 |

Denolle [46]

| Methods | Study design: multicentre RCT  Study duration: not reported  Study follow-up: 7 days |
| --- | --- |
| Participants | Country: France  Setting: eight hospitals with obstetric departments  Number: 57 (9 excluded due to 3 deliveries 4 hospitalisation) – 48 analysed (24 intervention 24 control)  Education level: not reported  Mean age (years): not reported  Inclusion criteria: patients with hypertension, defined as the mean of three office BP measurements during a single visit ≥ 140/90 and ≤ 180/105 mm Hg following 18 weeks gestation but without albuminuria or a history of hypertension in previous pregnancies or preceding the present pregnancy.  Exclusion criteria: BMI ≤ 30 kg/m2, did not have nephropathy or chronic disease, and were not undergoing treatment for hypertension |
| Interventions | Intervention type classification: self-monitoring, decision aid (communication with clinician)  Intervention group: remote BP monitoring linked with web based platform with communication with clinician  Control group: remote BP monitoring however the BP results were not given to the obstetrician, the patient had usual obstetric monitoring |
| Outcomes | Primary objective: the prevalence and prognosis of white coat hypertension (WCH) in women with mild hypertension.  Secondary objectives: to study: a) the feasibility and safety of home BP measured telemetrically in hypertensive pregnant women. The feasibility was assessed by the percentage of validated HBP monitorings and measurements.  The tolerance and acceptability of HBPM was assessed via questionnaire. Safety was assessed by the obstetrician at the end of the study. The time needed to be taught by the nurse and to monitor BP at home was noted, and cost saving when using HBP measured telemetrically compared with standard BP monitoring. This cost saving was assessed by the number of visits, hospitalizations, tests, and sick days in each group |
| Notes | No other publications for this study identified  Funding source: none reported |

Deshpande [53]

| Methods | Study design: cohort study  Study duration: 6 months (November 2020 – April 2021)  Study follow-up: 90 days |
| --- | --- |
| Participants | Country: India  Setting: Hospital  Number: 63 (156 approached), 3 opted out prior to completion of follow up  Education level: not reported  Mean age (years): not reported  Inclusion criteria: women who underwent vaginal delivery with HDP without any complication and willing to participate in the study and having digital BP apparatus and mobile phone with WhatsApp application at home. Birth companion willing to record BP and inform obstetrician.  Exclusion criteria: postnatal women with eclampsia or HDP with complications,  those not willing to participate in the study, women who underwent caesarean section, women readmitted to hospital for neonatal complication. |
| Interventions | Intervention type classification: self-monitoring, decision aid (communication with clinician)  Intervention group: remote blood pressure monitoring program with text message communication with clinician  Control group: nil |
| Outcomes | Primary outcome: feasibility of BP monitoring in postpartum women by teleconsultation, feasibility of antihypertensive dose adjustment  through telemedicine, pattern of BP normalization at home, number of women requiring hospital visit for uncontrolled blood pressure or warning signs and symptoms |
| Notes | No other publications for this study identified  Funding source: none reported |

Eedarapalli [54] poster

| Methods | Study design: cohort study  Study duration: 12 months (July 2017-June 2018)  Study follow-up: not reported |
| --- | --- |
| Participants | Country: UK  Setting: 2 hospitals and community  Number: 131  Education level: not reported  Mean age (years): not reported  Inclusion criteria: asymptomatic women at gestations 20–37 + 6 with BP = <149/ 99 + no proteinuria or BP = <145/95 + 1 + proteinuria (PCR <30) with normal or mildly deranged blood tests; clinically stable postnatal women < Day10  Exclusion criteria: not reported |
| Interventions | Intervention type classification: self-monitoring, decision aid (communication with clinician)  Intervention group: remote blood pressure monitoring program with text messaging of BPs linked with web based platform, communication with clinician  Control group: nil |
| Outcomes | Primary outcome: clinical outcomes, admission rates, patient satisfaction |
| Notes | No other publications for this study identified  Funding source: none reported |

Fazal [55]

| Methods | Study design: retrospective cohort study  Study duration: 12 months (June 2017- June 2018)  Study follow-up: up to 20 weeks (from recruitment until delivery) |
| --- | --- |
| Participants | Country: UK  Setting: Hospital  Number: 75  Education level: not reported  Mean age (years): not reported  Inclusion criteria: women diagnosed with chronic hypertension or a HDP at the time of delivery discharge were included  Exclusion criteria: not reported |
| Interventions | Intervention type classification: self-monitoring, decision aid (communication with clinician)  Intervention group: remote blood pressure monitoring program with communication with clinician via text message communication with web-based platform storage of information  Control group: nil |
| Outcomes | Primary outcome: patient satisfaction and reduction in face-to-face visits to healthcare facilities (DAU, community midwife/GP)  Secondary outcome: financial cost saving |
| Notes | No other publications for this study identified  Funding source: Great Western Hospital provided the Equipment cost (BP monitor and urine dipstick), Gant/award no: Not applicable. Maternity safety and innovation Fund 2016–2017 from the Department of Health was Used to cover midwifery champion’s time. It had no contribution to study design and no influence on the outcome. Grant/award no.: not available. West of England Academic Health Science Network (WEAHSN)’s QI/Innovation fund to support a quality improvement/  innovation project that was used for the purchase of License (Florence). It did not  influence the study design or outcome. Grant/award no: not available |

Ganapathy [56]

| Methods | Study design: cohort study  Study duration: 12 months (9/2018-8/2019)  Study follow-up: not reported |
| --- | --- |
| Participants | Country: UK  Setting: Hospital  Number: 50  Education level: not reported  Mean age (years): not reported  Inclusion criteria: women who were admitted to city hospital  Exclusion criteria: not reported |
| Interventions | Intervention type classification: self-monitoring, decision aid (communication with clinician)  Intervention group: remote BP monitoring with Bluetooth BP cuff with communication via mobile phone application, text message and management of data via web based platform  Control group: nil |
| Outcomes | Primary outcome: technological feasibility and acceptance by women |
| Notes | No other publications for this study identified  Funding source: development was self-funded by authors 1 and 2- R. Ganapathy  and A. Grewal. |

Goodin [105] poster

| Methods | Study design: cohort study  Study duration: not reported  Study follow-up: 6 weeks |
| --- | --- |
| Participants | Country: USA  Setting: Hospital  Number: 401  Education level: not reported  Mean age (years): not reported  Inclusion criteria: postpartum women with health risks in the initial 6 weeks postpartum  Exclusion criteria: not reported |
| Interventions | Intervention type classification: decision aid  Intervention group: clinical review via a smartphone app with telehealth communication with clinician  Control group: nil |
| Outcomes | Primary outcome: usage of app, clinical outcomes |
| Notes | No other publications for this study identified  Funding source: none reported |

Hackeloeer [106] poster

| Methods | Study design: cohort study  Study duration: 17 months (11/2020 - 03/2022)  Study follow-up: not reported |
| --- | --- |
| Participants | Country: Germany  Setting: not reported  Number: 48 (outcome available in 30 patients)  Education level: not reported  Mean age (years): not reported  Inclusion criteria: not reported  Exclusion criteria: not reported |
| Interventions | Intervention type classification: self-monitoring  Intervention group: remote blood pressure monitoring program with submission of BP via app  Control group: nil |
| Outcomes | Primary outcome: adverse preeclampsia-related pregnancy outcomes, blood pressure (systolic, diastolic, mean arterial pressure) |
| Notes | No other publications for this study identified  Funding source: none reported |

Hacker [88]

| Methods | Study design: prospective cohort study  Study duration: 12 months (July 2020-June 2021)  Study follow-up: not reported |
| --- | --- |
| Participants | Country: USA  Setting: Tertiary hospital  Number: 1192  Education level: not reported  Mean age (years): 31.1  Inclusion criteria: all postpartum discharges from July 2020 through June 2021 at our institution  Exclusion criteria: if they did not have a cuff or if they had a pre-pregnancy diagnosis of hypertension or a hypertensive disorder of pregnancy |
| Interventions | Intervention type classification: self-monitoring, decision aid (communication with clinician)  Intervention group: remote BP monitoring with telehealth communication with clinician  Control group: nil |
| Outcomes | Primary outcome: feasibility of the remote BP monitoring program - quantified by the proportion of women for whom blood pressure was ascertained, resource utilization, and identification and triage to appropriate follow up.  Secondary outcome: additional outcomes of interest included the incidence of new-onset postpartum preeclampsia, postpartum systolic and diastolic blood pressure values, enrolment into our remote monitoring blood pressure program, and Emergency Department evaluation and hospital readmission for postpartum hypertension. |
| Notes | No other publications for this study identified  Funding source: the project described was supported by the Magee-Womens Hospital Medical Staff through Grant Award FY2021 and by the National Institute of Health through Grant Number UL1 TR001857 to Dr. Hacker This work was additionally supported by NIH/ORWH Building Interdisciplinary Research Careers in Women’s Health (BIRCWH) NIH K12HD043441 scholar funds to Dr. Hauspurg. |

Hauspurg [57]

| Methods | Study design: cohort study  Study duration: 12 months (February 2018-January 2019)  Study follow-up: 6 weeks |
| --- | --- |
| Participants | Country: USA  Setting: Hospital  Number: 499 (409 in final analysis)  Education level: not reported  Mean age (years): 31  Inclusion criteria: women with one of the following hypertension-related diagnoses: chronic hypertension, chronic hypertension with superimposed preeclampsia, gestational hypertension, preeclampsia, eclampsia, or new onset postpartum hypertension, be English-speaking and are also required to have access to a text messaging-enabled smartphone device.  Exclusion criteria: not reported |
| Interventions | Intervention type classification: self-monitoring, decision aid (communication with clinician)  Intervention group: remote BP monitoring with web based platform and text message communication with clinician  Control group: nil |
| Outcomes | Primary outcome: feasibility - engagement and retention through 42 days postpartum, patient acceptability (assessed via a post program survey administered through the telehealth platform) |
| Notes | No other publications for this study identified  Funding source: This work was supported by institutional funds and NIH/ORWH Building Interdisciplinary Research Careers in Women’s Health (BIRCWH) NIH K12HD043441 scholar funds to AH. |

Hinton [58] - BuMP study

| Methods | Study design: qualitative study of retrospective cohort study [66]  Study duration: 10 months (April 2013- January 2014)  Study follow-up: up to 34 weeks (recruitment at 12-16 weeks with follow up to 6 weeks postpartum) |
| --- | --- |
| Participants | Country: UK  Setting: hospital  Number: 170 in the original study [66], 15 patients participated in the qualitative assessment of their experience  Education level: 7 first degree, 1 GCSE O level or CSE, 3 professional qualification, 3 post graduate or above, 1 unkonwn  Mean age (years): 33.73  Inclusion criteria: women who were deemed at higher risk of preeclampsia (who had any of: previous pre-eclampsia, family history of pre-eclampsia, hypertension, pregnancy  interval of 10 years, age 40 or over, high BMI, first or multiple pregnancy, renal disease) were invited at between 12 and 16 weeks  Exclusion criteria: not reported |
| Interventions | Intervention type classification: self-monitoring, decision aid  Intervention group: remote BP monitoring with automated BP cuff that transmits data to web based platform as well as via text message  Control group: nil |
| Outcomes | Primary outcome: qualitative assessment of participant experience of remote self BP monitoring |
| Notes | Qualitative study of Tucker [66]  Funding source: This study was funded through a grant from the National Institute for Health Research (NIHR) National School for Primary Care Research (SPCR) (SPCR project No. 171 |

Hirshberg 2023 [96]

| Methods | Study design: retrospective cohort study  Study duration: 3 years and 7 months (September 2017 - April 2021)  Study follow-up: 10 days for BP monitoring, 6 months for postpartum complications |
| --- | --- |
| Participants | Country: USA  Setting: multi centre, 3 hospitals  Number: 1,700 intervention, control 1,021 individuals in cohort A and 1,276 individuals in cohort C.  Education level: not reported  Mean age (years): intervention 32.22, cohort A 30.29, cohort C 29.73  Inclusion criteria: Patients across three Penn-Medicine affiliated obstetric hospitals who were diagnosed with hypertensive disorders of pregnancy with a delivery from September 2017 to April 2021 and had medical coverage through Independence Blue Cross (Independence). These patients were diagnosed with gestational hypertension; preeclampsia; chronic hypertension with superimposed preeclampsia; hemolysis, elevated liver enzymes, and low platelet count (HELLP) syndrome; or eclampsia  Exclusion criteria: members who had less than 16 months of continuous insurance enrolment before delivery, patients who incurred more than $12,500 in total medical costs per member per month in the prenatal period, patients with multiple deliveries during the study time frame were only included once. |
| Interventions | Intervention type classification: self-monitoring, decision aid (communication with clinician)  Intervention group: remote self BP monitoring with text message and telehealth communication  Control group: the asynchronous  comparison group (cohort A) included  members who met remote monitoring program inclusion criteria at any of the three participating hospitals between August 2015 and August 2017, before the  program was implemented. The contemporaneous comparison group (cohort C) was comprised of members  who also met the program’s inclusion criteria but delivered at hospitals other than the three intervention hospitals during the same time that the program was used as standard clinical care. |
| Outcomes | Primary outcome: a composite  measure of having any prespecified adverse clinical outcome after delivery discharge, including stroke, disseminated intravascular coagulation, eclampsia,  pulmonary edema, HELLP syndrome, myocardial infarction, and cardiomyopathy.  Secondary outcomes: total medical cost and health care service utilization including specialist visits, emergency department (ED) visits, cardiology visits, and all-cause inpatient readmissions in the first 6 months after delivery. |
| Notes | No other publications for this study identified  Funding source: none reported |

Hirshberg [59] poster 2016 and paper [67] 2017

| Methods | Study design: cohort study  Study duration: 4 months (Sep-December 2014)  Study follow-up: 10 days |
| --- | --- |
| Participants | Country: USA  Setting: hospital  Number: 32  Education level: not reported  Mean age (years): not reported  Inclusion criteria: women who delivered between September – December 2014 with chronic hypertension, gestational hypertension, preeclampsia  Exclusion criteria: not reported |
| Interventions | Intervention type classification: self-monitoring, decision aid (communication with clinician)  Intervention group: remote BP monitoring with electronic BP cuffs with text message communication  Control group: nil |
| Outcomes | Primary outcome: follow up rate, participation rate (BPs sent), clinical outcomes |
| Notes | No other publications for this study identified  Funding source: none reported |

Hirshberg [60] research letter 2019

| Methods | Study design: secondary analysis of previous RCT (Hirshberg [61] 2018  Study duration: 5 months (August 2016- January 2017)  Study follow-up: 10 days (for BP measurement), 6 weeks for follow up attendance rate |
| --- | --- |
| Participants | Country: USA  Setting: Hospital  Number: 206 (103 intervention 103 control)  Education level: not reported  Mean age (years): not reported in this study, but original study - intervention 28, control 28  Inclusion criteria: All women with  pregnancy-related hypertension (gestational hypertension, preeclampsia, chronic hypertension with superimposed  pre-eclampsia, or haemolysis, elevated liver enzymes, low platelets syndrome (with or without inpatient hypertension) who delivered at the home institution,  over 18 years of age, be able to speak and read English and have access to a cellphone with unlimited text  message capabilities  Exclusion criteria: readmissions for new-onset postpartum hypertension |
| Interventions | Intervention type classification: self-monitoring, decision aid (communication with clinician)  Intervention group: remote BP monitoring with text message communication and data storage on web based platform  Control group: usual care (in-person office-based review and blood pressure assessment) |
| Outcomes | Primary outcome: ascertainment of blood pressure, defined as either office visit attendance or at least 1 blood pressure texted.  Secondary outcomes: hypertension related readmission, rate of oral antihypertensive medication requirement  This study analysed the racial disparities in the patient outcomes |
| Notes | This is a secondary analysis of previous RCT (Hirshberg [61] 2018)  Funding source: none reported |

Hirshberg [61] 2018

| Methods | Study design: RCT  Study duration: 6 months (August 2016- January 2017)  Study follow-up: 10 days (for BP measurement), 6 weeks for follow up attendance rate |
| --- | --- |
| Participants | Country: USA  Setting: Hospital  Number: 206 (103 intervention 103 control, 62 intervention completed follow up survey, 77 control completed follow up survey)  Education level: not reported  Mean age (years): intervention 28, control 28  Inclusion criteria: All women with  pregnancy-related hypertension (gestational hypertension, preeclampsia, chronic hypertension with superimposed  pre-eclampsia, or haemolysis, elevated liver enzymes, low platelets syndrome (with or without inpatient hypertension) who delivered at the home institution were eligible. Women had to be  over 18 years of age, be able to speak and read English and have access to a cellphone with unlimited text  message capabilities  Exclusion criteria: readmissions for new-onset postpartum hypertension were not eligible. |
| Interventions | Intervention type classification: self-monitoring, decision aid (communication with clinician)  Intervention group: remote BP monitoring with text message communication, data storage on web based platform  Control group: usual care (in-person office-based review and blood pressure assessment) |
| Outcomes | The primary outcome was ascertainment of blood pressure, defined as either office visit attendance or at least 1 blood pressure texted.  Secondary outcome measures were initiation of antihypertensive medication, number of additional postpartum office or emergency room visits and readmission for persistent hypertension, attendance of the 4–6 week postpartum visit, patient satisfaction with blood pressure surveillance and future health awareness in relation to the long-term effects of pre-eclampsia on cardiac health. |
| Notes | There is a secondary analysis of this study – Hirshberg [60] research letter 2019. The intervention cohort in this study served as control cohort in another study - Triebwasser [42].  Funding source: none reported |

Hoppe [74] 2019

| Methods | Study design: cohort study  Study duration: 4 months (March 23, 2017-July 14, 2017)  Study follow-up: 6 weeks |
| --- | --- |
| Participants | Country: USA  Setting: Hospital  Number: 55 (124 approached)  Education level: not reported  Mean age (years): 31.8  Inclusion criteria: women admitted to the labor and delivery unit of a Midwestern academic hospital with the following inclusion criteria: ≥18 years old, with one of the following hypertension-related diagnoses during pregnancy: chronic, gestational, preeclampsia, or eclampsia, or a new hypertension diagnosis postpartum  Exclusion criteria: women who were readmitted after their primary hospital admission for delivery of their neonate. |
| Interventions | Intervention type classification: self-monitoring, decision aid (communication with clinician)  Intervention group: remote BP monitoring with tablet device and Bluetooth BP cuff with telehealth review with clinician  Control group: nil |
| Outcomes | Primary outcome: feasibility – measured through recruitment, consent and retention through 42 days postpartum.  Secondary outcomes: incidence of severe hypertension (systolic BP ≥160 or diastolic BP ≥110 mmHg) after discharge, postpartum blood pressure requiring treatment (systolic BP ≥150 mmHg or diastolic BP≥100 mmHg), participant evaluation of the equipment and satisfaction, total participant contacts required, and hospital readmission at 6 weeks postpartum |
| Notes | No other publications for this study identified  Funding source: this project was supported by the UnityPoint Health-Meriter Foundation and the University of Wisconsin Department of Obstetrics & Gynecology intramural departmental funding. |

Hoppe [75] 2020

| Methods | Study design: non randomised controlled trial  Study duration: 14 months (April 2017-June 2018)  Study follow-up: 6 weeks |
| --- | --- |
| Participants | Country: USA  Setting: hospital  Number: 428 (214 intervention 214 control)  Education level: not reported  Mean age (years): 31.8  Inclusion criteria: women admitted for  delivery of their neonate with any of the following hypertensive diagnoses: chronic hypertension, gestational  hypertension, preeclampsia or eclampsia  Exclusion criteria: not reported |
| Interventions | Intervention type classification: self-monitoring, decision aid (communication with clinician)  Intervention group: remote BP monitoring with tablet device and Bluetooth BP cuff with telehealth review from clinician  Control group: standard outpatient care |
| Outcomes | Primary outcome: hypertension related readmission through six-weeks postpartum  Secondary outcomes: hypertension related postpartum emergency room (ER)/triage visit, acquisition of BP within ten days of delivery, and use of antihypertensives six-weeks postpartum. |
| Notes | No other publications for this study identified  Funding source: this project was supported by the UnityPoint Health – Meriter Foundation and the University of Wisconsin Department of Obstetrics & Gynecology intramural departmental funding and in part by the Clinical and Translation Science Award UL1 TR002372 to the University of Wisconsin-Madison from the National Center for Advancing Translational Science, NIH, DHHS. |

Huber [76] poster

| Methods | Study design: cohort study  Study duration: not reported  Study follow-up: 8 weeks |
| --- | --- |
| Participants | Country: USA  Setting: hospital  Number: not reported  Education level: not reported  Mean age (years): not reported  Inclusion criteria: not reported  Exclusion criteria: not reported |
| Interventions | Intervention type classification: self-monitoring, decision aid (communication with clinician)  Intervention group: remote BP monitoring with storage of data via Bluetooth capable iPad and communication via telehealth  Control group: usual care |
| Outcomes | Primary outcome: postpartum hypertension readmission rates, patient compliance with blood pressure medication management |
| Notes | No other publications for this study identified  Funding source: none reported |

Jones [89]

| Methods | Study design: mixed methods pilot study with a survey and semi-structured qualitative interview  Study duration: not reported  Study follow-up: survey and interview conducted after 2 weeks of remote BP monitoring |
| --- | --- |
| Participants | Country: USA  Setting: hospital  Number: 12 patients consented, 11 patients in final analysis, (1 lost to follow up)  Education level (n, %): 9^th^ grade to 12^th^ grade 1, 9.09%, high school graduate or GED 3 (27.27%), some college or technical school 5 (45.45%), college graduate or higher 2 (18.18%)  Mean age (years): not reported  Inclusion criteria: pregnant females in their second trimester between18-50 years of age whose prenatal care was received at the [institution blinded for peer review] Women’s Health Clinic and who (b) had elevated BP meeting the criteria of systolic ≥ 140 and/or dia­stolic ≥ 90 for at least one reading  Exclusion criteria: not reported |
| Interventions | Intervention type classification: self-monitoring, decision aid  Intervention group: remote BP monitoring with BP cuff that transmits data via cellular capability to a web based platform with telehealth communication with clinician  Control group: nil |
| Outcomes | Primary outcome: patients’ satisfaction with and feasibility of using the remote BP monitoring program |
| Notes | No other publications for this study identified  Funding source: Translational Research Institute at the University of Arkan­sas for Medical Sciences. |

Jongsma [77] – alternate analysis of van den Heuvel [15] 2020

| Methods | Study design: mixed methods study (questionnaire and interviews) of prospective case control study [15]  Study duration: 14 months (October 2017- December 2018)  Study follow-up: questionnaire given at 36 weeks gestation, original study up to 24 weeks (for duration of pregnancy, earliest BP monitoring from 16 weeks) |
| --- | --- |
| Participants | Country: Netherlands  Setting: hospital  Number: 51 patients  Education level (n, %): primary school 1, 1.9, secondary school 4, 7.7, middle-level applied education 14, 26.9, higher-level applied education 17, 32.7, scientific education (university) 13, 25, unknown 3, 5.8  Mean age (years): 34.4  Inclusion criteria: patients from original study who could speak either Dutch or English and were willing to be interviewed  Exclusion criteria: not reported |
| Interventions | Intervention type classification: self-monitoring, decision aid (communication with clinician)  Intervention group: remote BP monitoring with Bluetooth transmission of data to a smartphone application and web based platform  Control group: nil |
| Outcomes | Primary outcome: patients experiences of and motivations for using the remote BP monitoring technology |
| Notes | This is a mixed methods study (questionnaire and interviews) of prospective case control study [15]  Funding source: none reported |

Kalafat [107]

| Methods | Study design: cohort study  Study duration: 8 months (December 2017 - August 2018)  Study follow-up: ~6 weeks (median recruitment to study 34 weeks, median delivery gestation 38.9 weeks) |
| --- | --- |
| Participants | Country: UK  Setting: hospital  Number: 143 total (80 intervention, 63 control)  Education level: not reported  Mean age (median): median age intervention 34, median age control 31  Inclusion criteria: have an initial diagnosis of gestational hypertension (GH)  Exclusion criteria: diagnosis of chronic hypertension, maternal age less than 16 years, systolic BP above 155 mmHg, diastolic BP above 100 mmHg, significant  proteinuria (≥2+ on dipstick testing or protein/creatinine ratio>30mg/mmol), an estimated fetal weight below the 10th centile, signs of severe preeclampsia (oliguria<500 mL/24 h, cerebral or visual disturbance, pulmonary edema, epigastric or right-upper quadrant pain,  impaired liver function, platelet count <100,000/mm3), significant mental health concerns or insufficient understanding of the English language. |
| Interventions | Intervention type classification: self-monitoring, decision aid (communication with clinician)  Intervention group: remote home BP monitoring with communication via smartphone app  Control group: retrospective cohort of pregnant women who presented to Day Assessment Unit (DAU) with GH, managed via the traditional  pathway (hospital BP monitoring) |
| Outcomes | Primary outcome: diagnosis of HDP, maternal and foetal clinical outcomes |
| Notes | No other publications for this study identified  Funding source: this study was supported by a grant from the Health Foundation. |

Khosla [90]

| Methods | Study design: retrospective cohort study  Study duration: 6 months (December 2019 - June 2020)  Study follow-up: 6 weeks |
| --- | --- |
| Participants | Country: USA  Setting: hospital (tertiary care centre)  Number: 473 total (intervention 258, control 215)  Education level: not reported  Mean age (years): intervention 30, control 29  Inclusion criteria: all patients attending the postpartum hypertension clinic – this includes all patients with HDP who delivered at that hospital between December 2019-June 2020  Exclusion criteria: not reported |
| Interventions | Intervention type classification: self-monitoring, decision aid (communication with clinician)  Intervention group: telehealth review with clinicians in conjunction with a bundle of quality improvement initiatives targeting postpartum hypertension (including remote BP monitoring), these patients delivered from March 15, 2020 onwards  Control group: historical group of patients with face to face review in conjunction with a bundle of quality improvement initiatives targeting postpartum hypertension (including remote BP monitoring), these patients delivered between December 1 2019 and February 14, 2020 |
| Outcomes | Primary outcome: the change in racial disparity for the first postpartum hypertension clinic (PPHTN) visit attendance between the pre- and post-telehealth periods  Secondary outcomes: the proportion of patients who attended a 6-week general postpartum visit and the proportion with hypertensive-range BP (≥140/90) at the time of the first PPHTN visit and the general 6-week postpartum visit |
| Notes | No other publications for this study identified  Funding source: none reported |

Kitt [24] – prospective long term follow up of study by Cairns [51]

| Methods | Study design: prospective long term follow up of previous unmasked randomised trial [51]  Study duration: 8 months (1 April 2019- 1 December 2019)  Study follow-up: ~ 4 years (median 3.6 ± 0.4 years postpartum) |
| --- | --- |
| Participants | Country: UK  Setting: multiple sites (5 NHS hospitals)  Number: 61 (30 intervention, 31 control)  Education level: not reported  Mean age (at re-recruitment): intervention 35.2, control 34.1  Inclusion criteria: original study inclusion criteria - women aged ≥18 years, with gestational hypertension or preeclampsia requiring antihypertensive treatment, were eligible, 70 of 101 original study participants gave consent to be contacted for future studies  Exclusion criteria: this study’s exclusion criteria - if they were pregnant at the time of reassessment or they had a significant new comorbidity that made enrolment unsafe, current antihypertensive use. Original study exclusion criteria - prescription of >3 antihypertensive medications, self-report of hypertension diagnosed outside of pregnancy, and inability to speak English |
| Interventions | Intervention type classification: self-monitoring, decision aid (communication with clinician)  Intervention group: in the original study these patients performed remote self BP monitoring with communication of BP via text message or smartphone app with clinician  Control group: usual care (usual care patients had their BP monitored by their  community midwife and their antihypertensive medication adjusted  by their general practitioner) |
| Outcomes | Primary outcome: blood pressure (3-4 years after initial remote BP monitoring program)  Secondary outcome: 24 hour diastolic BP, antihypertensives prescribed |
| Notes | This was a prospective long term follow up of previous unmasked randomised trial - Cairns [51]  Funding source: R. McManus and P. Leeson acknowledge support from the National Institute for Health Research (NIHR) Oxford Collaboration for Leadership in Health Research and Care. J. Kitt is funded by a British Heart Foundation Clinical Research Training Fellowship (British Heart Foundation grant number FS/19/7/34148). P. Leeson  acknowledges support from the Oxford British Heart Foundation Centre for  Research Excellence and NIHR Oxford Biomedical Research Centre. K. Tucker  and R. McManus are funded by an NIHR programme grant for applied research  (RP-PG-0614-20005) and have received funding from the NIHR Applied Research  Collaboration Oxford and Thames Valley at Oxford Health National Health Service Foundation Trust. R. McManus is an NIHR Senior Investigator. |

Lanssens [95] poster 2016

| Methods | Study design: retrospective cohort study  Study duration: 12 months (1 January – 31 December 2015)  Study follow-up: not reported |
| --- | --- |
| Participants | Country: Belgium  Setting: outpatient clinic of prenatal centre  Number: 142 total (44 intervention, 98 control)  Education level: not reported  Mean age (years): not reported  Inclusion criteria: pregnant women with GHD who delivered at Ziekenhuis Oost-Limburg in 2015 and had a RM prenatal follow up  Exclusion criteria: not reported |
| Interventions | Intervention type classification: self-monitoring, decision aid (communication with clinician)  Intervention group: remote BP monitoring with transmission of data via Wi-Fi or Bluetooth, data storage on web based portal and telehealth communication with clinician  Control group: conventional care |
| Outcomes | Primary outcome: number of prenatal consultations and Maternal Intensive Care (MIC) admissions.  Secondary outcomes: maternal and neonatal outcomes |
| Notes | These patients likely included in other study by authors (though not explicitly reported) Lanssens [83]  Funding source: none reported |

Lanssens [83] 2017 - PREMOM

| Methods | Study design: retrospective cohort study  Study duration: 12 months (1 January – 31 December 2015)  Study follow-up: 145 days |
| --- | --- |
| Participants | Country: Belgium  Setting: outpatient clinic of prenatal centre  Number: 166 total (146 in final analysis)- 53 intervention (48 included in final analysis) 113 control (98 included in final analysis)  Education level: not reported  Mean age (years): intervention 31.69, control 31.94  Inclusion criteria: all women diagnosed with GHD who delivered at the outpatient prenatal clinic of Ziekenhuis Oost-Limburg (Genk, Belgium) during 2015 were included. The criteria to initiate RM were GHD at gestational age ≥20 weeks where an intensive follow-up until delivery was desirable.  Exclusion criteria: women without a mobile phone, a gestational age less than 20 weeks, a fetus with congenital malformations, and women who refused informed consent were excluded and received conventional care (CC). |
| Interventions | Intervention type classification: self-monitoring, decision aid (communication with clinician)  Intervention group: remote BP monitoring with transmission of data via Wi-Fi or Bluetooth, data storage on web based portal and telehealth communication with clinician  Control group: conventional care |
| Outcomes | Primary outcome: total numbers of prenatal consultations were collected from 10 weeks of gestation onwards: ultrasound scans, cardiotocographics (CTG), admission to the prenatal ward, total days of hospitalization, and the number of admissions until delivery.  Secondary outcomes: maternal parameters collected at birth were gestational age at delivery and mode of delivery. Neonatal outcomes collected were birth weight, birth weight percent, length, Apgar at 1′ and 5′, and number of admissions to NIC. |
| Notes | The patients included in this study was also included in Lanssens [91] 2018 Funding source: none reported |

Lanssens [91] 2018

| Methods | Study design: retrospective cohort study  Study duration: 2 years (1/1/2015-31/12/2016)  Study follow-up: up to 30 weeks (from recruitment at ~ 10 weeks until delivery) |
| --- | --- |
| Participants | Country: Belgium  Setting: outpatient antenatal clinic  Number: 320 total (301 in final analysis, 90 (86 in final analysis) intervention, 230 (215 in final analysis) control)  Education level: not reported  Mean age (years): intervention 30.97, control 30.53  Inclusion criteria: all women diagnosed with gestational hypertensive disordres (GHD) who underwent prenatal follow-up at the outpatients prenatal clinic of Ziekenhuis Oost- Limburg (Genk, Belgium) during 2015 and 2016, gestational age of >10 weeks  Exclusion criteria: not reported |
| Interventions | Intervention type classification: self-monitoring, decision aid (communication with clinician)  Intervention group: remote BP monitoring with wireless BP monitor and transmission of data to web based platform with telehealth review by clinician  Control group: Women at a gestational age of <10 weeks, or women who did not give their informed consent received CC |
| Outcomes | Primary outcome: the total number of prenatal consultations from the start of the pregnancy: ultrasound scans, cardiotocography, admission to the prenatal ward, total days of hospitalization, and the number of admissions until delivery.  Secondary outcome: the maternal parameters collected at birth were gestational age at delivery, intended mode of delivery, and mode of delivery. The neonatal outcomes collected were birth weight, Apgar score at 1 min and 5 min, and number of admissions to the NICU |
| Notes | A cost analysis of this project was published as a separate paper [25] as well as analysis of perception and experiences of participants Lanssens [82] 2019  Funding source: this study is part of the Limburg Clinical Research Program (LCRP) UHasselt-–ZOL–Jessa, supported by the Foundation Lim-burg Sterk Merk, the province of Limburg, the Flemish Govern- ment, Hasselt University, Ziekenhuis Oost-Limburg, and Jessa Hospital. This work was supported by Foundation Mustela (Laureate 2016). |

Lanssens [25] 2018 (cost)

| Methods | Study design: cost analysis of original retrospective cohort study Lanssens [83] 2017  Study duration: not reported, original study 12 months (1 January – 31 December 2015)  Study follow-up: mean time 44.42 days |
| --- | --- |
| Participants | Country: Belgium  Setting: outpatient clinic of prenatal centre  Number: 166 total (146 in final analysis)- 53 intervention (48 included in final analysis) 113 control (98 included in final analysis)  Education level: not reported  Mean age (years): intervention 31.72, control 31.95  Inclusion criteria: all women diagnosed with GHD who delivered at the outpatient prenatal clinic of Ziekenhuis Oost-Limburg (Genk, Belgium) during 2015, the criteria to initiate RM were GHD at gestational age ≥20 weeks where an intensive follow-up until delivery was desirable  Exclusion criteria: W\women without a mobile phone, a gestational age less than 20 weeks, a fetus with congenital malformations, and women who refused informed consent were excluded and received conventional care (CC). |
| Interventions | Intervention type classification: self-monitoring, decision aid (communication with clinician)  Intervention group: remote BP monitoring with data storage on web based portal and telehealth communication with clinician  Control group: conventional care |
| Outcomes | Costs of remote monitoring versus conventional care relative to reported benefits |
| Notes | This is a cost analysis of original study Lanssens [83] 2017  Funding source: none reported |

Lanssens [82] 2019

| Methods | Study design: questionnaire of perception and experiences of participants in retrospective cohort study Lanssens [83] 2017  Study duration: 2015- April 2016, original study 12 months (1 January – 31 December 2015)  Study follow-up: 23 days |
| --- | --- |
| Participants | Country: Belgium  Setting: online questionnaire, original study - outpatient clinic of prenatal centre  Number: 91  Education level (n, %): lower secondary school 4, 9, higher secondary school 12, 26, high school 20, 43, university 11, 23  Mean age (years): not reported  Inclusion criteria: all original participants of PREMOM [83], original study inclusion criteria: all women diagnosed with GHD who delivered at the outpatient prenatal clinic of Ziekenhuis Oost-Limburg (Genk, Belgium) during 2015  Exclusion criteria: the 27 women who did not participate didn’t answer their phone, didn’t have an email address, or didn’t speak Dutch |
| Interventions | Intervention type classification: self-monitoring, decision aid (communication with clinician)  Intervention group: remote BP monitoring with transmission of data via Wi-Fi or Bluetooth, data storage on web based portal and telehealth communication with clinician  Control group: conventional care |
| Outcomes | Primary outcome: to investigate the perceptions and experiences of remote monitoring among mothers, midwives, and obstetricians who participated in the PREMOM study. |
| Notes | Analysis of perception and experiences of participants in retrospective cohort study Lanssens [83]  Funding source: none reported |

Lopez [78]

| Methods | Study design: cohort study  Study duration: 3 months (October-December 2017)  Study follow-up: up to 28 weeks (recruitment from 12 weeks, followed up to delivery) |
| --- | --- |
| Participants | Country: Peru  Setting: Health institution  Number: 20  Education level: not reported  Mean age (years): not reported  Inclusion criteria: not reported  Exclusion criteria: not reported |
| Interventions | Intervention type classification: self-monitoring  Intervention group: remote BP monitoring with a wearable device with data transmission to a phone application with data storage on a web based platform. The wearable device transmits information via the internet and Bluetooth  Control group: nil |
| Outcomes | Primary outcome: clinical outcomes (abnormal BP, maternal deaths, controlled BP) |
| Notes | No other publications for this study identified  Funding source: none reported |

Marko [108]

| Methods | Study design: prospective cohort study  Study duration: 6 months (July 2014- January 2015)  Study follow-up: up to 32 weeks (follow up from recruitment from 8 weeks to delivery) |
| --- | --- |
| Participants | Country: USA  Setting: hospital  Number: 8  Education level: 4 graduate, 1 undergraduate, 3 not applicable  Mean age (years): 29.63  Inclusion criteria: pregnant women between the ages of 18 to 40  years old presenting for routine prenatal care in the first trimester who self-reported regular usage of an iPhone and had a low-risk pregnancy  status per established guidelines  Exclusion criteria: not reported |
| Interventions | Intervention type classification: self-monitoring  Intervention group: remote BP monitoring with mobile phone application connected to a wireless weight scale and BP cuff  Control group: nil |
| Outcomes | Primary outcome: patient engagement (number of times patient interacted with app or recorded blood pressure reading), accuracy of remote patient monitoring compared to in-office measurements, efficacy of automatic alerts, patient satisfaction, clinical outcomes |
| Notes | No other publications for this study identified  Funding source: none reported |

Mussarat [26] poster – Connected MOM

| Methods | Study design: retrospective case control  Study duration: 4 years 3 months (October 2016-December 2020)  Study follow-up: not reported |
| --- | --- |
| Participants | Country: USA  Setting: single healthcare system  Number: 9689 total (3223 intervention 6466 control  Education level: not reported  Mean age (years): intervention 30.5, control 30.6  Inclusion criteria: all patients who delivered in a single healthcare system from October 2016 to December 2020  Exclusion criteria: not reported |
| Interventions | Intervention type classification: self-monitoring  Intervention group: remote BP monitoring with mobile system, Bluetooth BP cuff  Control group: standard care |
| Outcomes | Primary outcome: early detection of pregnancy associated hypertension (PAH; gestational hypertension, preeclampsia and eclampsia)  Secondary outcomes: mode of  delivery, postpartum hemorrhage, neonatal intensive care and small  for gestational age |
| Notes | No other publications for this study identified  Funding source: none reported |

Musyoka [27]

| Methods | Study design: cohort study  Study duration: not reported  Study follow up: up to 20 weeks (follow up from recruitment at 20+ weeks to delivery) |
| --- | --- |
| Participants | Country: Kenya  Setting: two hospitals  Number: 30  Education level: not reported  Mean age (years): not reported  Inclusion criteria: expectant mothers who were 20 weeks pregnant and above, in two level 5 hospitals in Embu and Kiambu Counties  Exclusion criteria: not reported |
| Interventions | Intervention type classification: self-monitoring, decision aid (communication with clinician)  Intervention group: remote BP monitoring with smartwatch linked via Bluetooth with a mobile with transmission and storage of information with a mobile phone application and web based platform  Control group: nil |
| Outcomes | Primary outcome: system responsiveness, cost effectiveness, system error rate, user experience (questionnaire) |
| Notes | No other publications for this study identified  Funding source: none reported |

Nakahara [118] poster

| Methods | Study design: retrospective cohort study  Study duration: 5 years (January 2015- January 2020)  Study follow-up: 7 days |
| --- | --- |
| Participants | Country: USA  Setting: hospital  Number: 3584 total (422 intervention, 3162 control)  Education level: not reported  Mean age (years): intervention 34, control 33  Inclusion criteria: all women with a  hypertensive disorder of pregnancy who delivered at that hospital from January 2015 to January 2020  Exclusion criteria: not reported |
| Interventions | Intervention type classification: self-monitoring  Intervention group: Connected Maternity Online Monitoring (MOM) – remote BP monitoring program with data storage on web based platform  Control group: traditional care |
| Outcomes | Primary outcome: to evaluate the  utility of Connected MOM in improving adherence to the recommended  postpartum BP evaluation.  Secondary aim: to compare the rate of severe maternal morbidity in intervention versus control population |
| Notes | No other publications for this study identified  Funding source: none reported |

Novoa [28] poster

| Methods | Study design: cohort study  Study duration: 9 months (July 2021 – March 2022)  Study follow-up: 6 weeks |
| --- | --- |
| Participants | Country: USA  Setting: hospital  Number: 570  Education level: not reported  Mean age (years): not reported  Inclusion criteria: not reported  Exclusion criteria: not reported |
| Interventions | Intervention type classification: self-monitoring, decision aid (communication with clinician)  Intervention group: remote BP monitoring with telematic data transmission to clinician  Control group: nil |
| Outcomes | Primary outcome: patient perception of remote BP monitoring program and knowledge of hypertensive disorders |
| Notes | No other publications for this study identified  Funding source: none reported |

Nuss [109] poster

| Methods | Study design: retrospective cohort study  Study duration: 4 years (1 January 2016 – 31 December 2019)  Study follow-up: 10 days |
| --- | --- |
| Participants | Country: USA  Setting: hospital  Number: 2132 total (612 intervention 1520 control)  Education level: not reported  Mean age (years): intervention 29, control 28  Inclusion criteria: all women admitted with HDP who delivered between Jan. 1st, 2016 - Dec. 31st, 2019  Exclusion criteria: nil reported |
| Interventions | Intervention type classification: self-monitoring, decision aid (communication with clinician)  Intervention group: remote BP monitoring with mobile phone app  Control group: one outpatient visit for a blood pressure check |
| Outcomes | Primary outcome: readmission due to hypertension complications |
| Notes | No other publications for this study identified  Funding source: none reported |

Onishi [29] poster

| Methods | Study design: case series  Study duration: not reported  Study follow-up: 6 months |
| --- | --- |
| Participants | Country: Japan  Setting: hospital  Number: 4  Education level: not reported  Mean age (years): not reported  Inclusion criteria: not reported  Exclusion criteria: not reported |
| Interventions | Intervention type classification: self-monitoring  Intervention group: remote BP monitoring with data storage on web based platform  Control group: nil |
| Outcomes | Primary outcome: clinical outcome |
| Notes | No other publications for this study identified  Funding source: none reported |

Patel [97] poster

| Methods | Study design: cohort study  Study duration: 6 months (October 2021 – March 2022)  Study follow-up: 6 weeks |
| --- | --- |
| Participants | Country: USA  Setting: hospital  Number: 207  Education level: not reported  Mean age (years): 31  Inclusion criteria: patients with HDP  Exclusion criteria: not reported |
| Interventions | Intervention type classification: self-monitoring, decision aid (communication with clinician)  Intervention group: remote BP monitoring with Bluetooth blood pressure cuff with telehealth clinician review  Control group: nil |
| Outcomes | Primary outcome: postpartum visit adherence  Secondary outcomes: incidence of hypertensive blood pressures at six weeks postpartum, incidence of at least one blood pressure recording among the intervention cohort within six weeks |
| Notes | No other publications for this study identified  Funding source: none reported |

Payakachat [30] – same study as Rhoads [79]

| Methods | Study design: qualitative study of non randomised cohort study - Rhoads [79]  Study duration: 5 months (October 2015 – February 2016)  Study follow-up: 2 weeks |
| --- | --- |
| Participants | Country: USA  Setting: tertiary hospital  Number: 37 total (21 intervention, 16 control)  Education level: not reported  Mean age (years): not reported  Inclusion criteria: women aged 18 years and older, who were delivering a baby at a university hospital between October 2015 and February 2016 with diagnosis of pre-eclampsia during pregnancy, intrapartum, or postpartum, and spoke English.  Exclusion criteria: if they had psychiatric disorders or did not have access to a telephone 24/7 |
| Interventions | Intervention type classification: self-monitoring, decision aid (communication with clinician)  Intervention group: remote BP monitoring with Bluetooth transmission of data to web-based platform and telehealth review by clinician  Control group: usual care |
| Outcomes | Primary outcome: perceptions of the remote monitoring system (telephone interviews) |
| Notes | Qualitative study of patient experiences from original study - Rhoads [79]  Funding source: none reported |

Pealing 2019 [63] – OPTIMUM- BP

| Methods | Study design: mixed methods unmasked RCT  Study duration: 2 years (December 2015- December 2017)  Study follow-up: up to 38 weeks (follow up from recruitment to 6 weeks postpartum) |
| --- | --- |
| Participants | Country: UK  Setting: four NHS maternity units  Number: total 158 – chronic hypertension intervention 55, control 31, gestational hypertension intervention 49, control 23  Education level (n, %): professional/ higher qualifications chronic hypertension intervention 36, 65, control 16, 52, gestational hypertension intervention 37, 76, control 20, 87, school qualifications chronic hypertension intervention 15, 27, control 14, 45, gestational hypertension intervention 9, 18, control 3, 13, no formal qualification chronic hypertension intervention 3, 6, control 1, 3, gestational hypertension intervention 1, 2, control 0, 0, not known chronic hypertension intervention 2, 4, control 1, 3, gestational hypertension intervention 1, 2, control 1, 4  Mean age (years): chronic hypertension intervention 35.9, control 35.5, gestational hypertension intervention 33.4, control 34.2  Inclusion criteria: women aged ≥18 years with a singleton pregnancy and with chronic or gestational hypertension,  without preeclampsia. Women with chronic hypertension (treated or not) could have a prenatal diagnosis of hypertension or sustained BP readings ≥140 mmHg systolic and/or ≥90 mmHg diastolic before 20 weeks’ gestation, and were recruited any time between booking and 23+6 weeks’ gestation. Women with gestational hypertension, defined as sustained BP readings ≥140 mmHg systolic and/or ≥90 mmHg diastolic after 20 weeks’ gestation and without proteinuria, were recruited between 20 and 37+6 weeks’ gestation.  Exclusion criteria: if they were unwilling to self- monitor, had insufficient understanding of the study or had preeclampsia at time of enrolment |
| Interventions | Intervention type classification: self-monitoring  Intervention group: remote BP monitoring with transmission of data via text message or app with data storage on web based platform  Control group: usual care – BP monitoring by local clinical team |
| Outcomes | Primary outcome: feasibility, measured by recruitment, discontinuation, and adherence and persistence with the self-monitoring protocol.  Secondary outcomes included several measures of BP control (to best inform the design and analysis of a future large  multi-site RCT), delivery outcomes and safety, health resource use, quality of life scores, antihypertensive prescribing behaviour and self-reported medication beliefs and adherence. |
| Notes | Qualitative study conducted on 41 of these participants [31] Pealing 2017  Funding source: none reported |

Pealing [31] 2022 – qualitative study of OPTIMUM – BP Pealing 2019 [63]

| Methods | Study design: qualitative study of original RCT - Pealing [63]  Study duration: 17 months (December 2015 – May 2017)  Study follow-up: not reported |
| --- | --- |
| Participants | Country: UK  Setting: 38 clinics (36 antenatal clinics and two maternity assessment clinics)  Number: 48  Education level: not reported  Mean age (years): not reported  Inclusion criteria: all the women who participated were involved in the OPTIMUM feasibility trial  Exclusion criteria: not reported |
| Interventions | Intervention type classification: self-monitoring  Intervention group: remote BP monitoring with transmission of data via text message or app with data storage on web based platform  Control group: usual care – BP monitoring by local clinical team |
| Outcomes | Primary outcome: experiences of the self-monitoring intervention including acceptability of SMBP to women and clinicians, use of SMBP by women and clinicians, the importance of clinical relationships |
| Notes | Qualitative study of original mixed-methods RCT [63]  Funding source: none reported |

Perry [114]

| Methods | Study design: case control study  Study duration: 3 years (December 2013 – November 2016)  Study follow-up: up to 9 weeks (from recruitment until delivery) |
| --- | --- |
| Participants | Country: UK  Setting: hospital  Number: 166 total (108 remote monitoring, 58 control)  Education level: not reported  Mean age (years): remote monitoring 32.5, control 32  Inclusion criteria: chronic hypertension, GH or high risk of developing PE, no significant proteinuria (≤ 1+ proteinuria on dipstick testing), and normal biochemical and hematological markers. Pregnant women in the HBPM group presented via referral either to the  hypertension clinic or to the DAU between December 2013 and November 2016  Exclusion criteria: not reported |
| Interventions | Intervention type classification: self-monitoring, decision aid (communication with clinician)  Intervention group: remote BP monitoring with data transmission and communication via mobile phone app  Control group: retrospective cohort of patients managed with routine care with regular DAU visits for BP monitoring |
| Outcomes | Primary outcome: Adverse maternal, foetal and neonatal outcomes, duration of outpatient monitoring, healthcare utilisation – outpatient visits, admissions, HDU admissions |
| Notes | Cost analysis of this study conducted and published in - Xydopoulos [113]  Funding source: none reported |

Rhoads [79]

| Methods | Study design: non randomised controlled study  Study duration: 2 weeks  Study follow-up: 2 weeks |
| --- | --- |
| Participants | Country: USA  Setting: hospital  Number: 50 total (2 excluded due to non adherence, 25 intervention, 23 control)  Education level (n, %): >high school intervention 14, 56, control 15, 65.2  Mean age (years): intervention 26.8, control 27.9  Inclusion criteria: women speak English,  be at least 18 years of age who delivered at the University of Arkansas for Medical Sciences during the study period, and  have a pregnancy that was complicated by preeclampsia  Exclusion criteria: having a diagnosis of a psychiatric disorder or not having telephone access. |
| Interventions | Intervention type classification: self-monitoring, decision aid (communication with clinician)  Intervention group: remote BP monitoring with Bluetooth transmission of data and storage on web-based platform with telehealth communication with clinician  Control group: routine care |
| Outcomes | Primary outcome: identify and examine the patient factors that influence a patient’s decision to enrol in remote patient monitoring using the m-health user cohort or m-health nonuser cohort, assess whether there were any differences between patients who chose m-health monitoring and those who did not regarding treatment adherence and health outcomes, and describe the level of perceived patient experience with the technology among those who enrolled in the m-health monitoring cohort |
| Notes | Qualitative data reported in Payakachat [30]  Funding source: none reported |

Rimsza [32] poster

| Methods | Study design: nested case control study  Study duration: not reported  Study follow-up: 14 days |
| --- | --- |
| Participants | Country: USA  Setting: hospital  Number: 171  Education level: not reported  Mean age (years): not reported  Inclusion criteria: patients with a pre-existing hypertension or hypertensive disorder of pregnancy  Exclusion criteria: not reported |
| Interventions | Intervention type classification: self-monitoring, decision aid (communication with clinician)  Intervention group: remote BP monitoring with text message communication  Control group: nil |
| Outcomes | Primary outcome: engagement (adherence to BP monitoring) |
| Notes | No other publications for this study identified  Funding source: none reported |

Robles Cuevas [115]

| Methods | Study design: cohort study  Study duration: not reported  Study follow-up: not reported |
| --- | --- |
| Participants | Country: Mexico  Setting: laboratory  Number: 7 patients  Education level: not reported  Mean age (years): not reported  Inclusion criteria: not reported  Exclusion criteria: not reported |
| Interventions | Intervention type classification: decision aid (communication with clinician)  Intervention group: Remote telemonitoring system via a mobile phone application (allows reporting of alarm symptom data, hypertension data)  Control group: nil |
| Outcomes | Primary outcome: user satisfaction |
| Notes | No other publications for this study identified  Funding source: none reported |

Runkle [110]

| Methods | Study design: prospective cohort study  Study duration: 9 months (June 2019 – March 2020)  Study follow-up: up to 14 weeks (follow up from recruitment at 26 weeks to delivery) |
| --- | --- |
| Participants | Country: USA  Setting: rural health clinic  Number: 30 (23 completed follow up and survey)  Education level: not reported  Mean age (years): 30.1  Inclusion criteria: pregnant women to participate in our study were to be between 26 and 40 weeks of gestation,  attending an MAHEC centering group, and who owned a smartphone and they were invited to participate  in the study  Exclusion criteria: Women with serious arrhythmia, severe blood flow problems, or blood disorders, as well as complicating factors such as common arrhythmias, ventricular premature  beats, atrial fibrillation, arteriosclerosis, poor perfusion, diabetes, preeclampsia or renal disease are at a higher risk for obtaining a BP reading error and  were excluded from participation in this study |
| Interventions | Intervention type classification: self-monitoring  Intervention group: remote BP monitoring with BP cuff and smartphone application with transmission and storage of data via web based platform  Control group: nil |
| Outcomes | Primary outcome: retention and persistence of weekly BP monitoring during late-stage pregnancy, differences between weekly self-monitored and clinic BP measures, and the performance of self-monitored BP in early detection of  pregnancy-induced HTN compared with the clinic visit  Secondary outcomes: receptivity to m-health technology for prenatal monitoring, e-health literacy, and prenatal care utilization and satisfaction |
| Notes | No other publications for this study identified  Funding source: Funding for this study was provided, in part, from the Undergraduate Research Assistantship Awards, Office of Student Research and Student and Faculty Excellence Fund (SAFE) at Appalachian State University |

Sabol [33] poster (925)

| Methods | Study design: cohort study  Study duration: 4 months (April 1 – July 31 2020)  Study follow-up: 14 days |
| --- | --- |
| Participants | Country: USA  Setting: hospital  Number: not reported  Education level: not reported  Mean age (years): not reported  Inclusion criteria: postpartum women with HDP or chronic hypertension who was English speaking and have access to text messaging  Exclusion criteria: not reported |
| Interventions | Intervention type classification: self-monitoring, decision aid (communication with clinician)  Intervention group: remote BP monitoring with text message and telehealth communication with clinician  Control group: historical cohort with usual care (prior to implementation of remote BP monitoring) |
| Outcomes | Primary outcome: postpartum readmission due to HDP |
| Notes | Other findings reported in Sabol [64]  Funding source: none reported |

Sabol [64] poster (879)

| Methods | Study design: cohort study  Study duration: 3 months (April 1 – June 30, 2020)  Study follow-up: 14 days |
| --- | --- |
| Participants | Country: USA  Setting: hospital  Number: 247  Education level: not reported  Mean age (years): not reported  Inclusion criteria: postpartum  women with any hypertensive disorder of pregnancy or chronic hypertension who was English speaking and have access to a text messaging.  Exclusion criteria: not reported |
| Interventions | Intervention type classification: self-monitoring, decision aid (communication with clinician)  Intervention group: remote BP monitoring with text message and telehealth communication with clinician  Control group: nil |
| Outcomes | Primary outcome: program reach (defined as the proportion of eligible women willing to participate) and individual level implementation (defined as adherence with texting at least 1 BP within 72 hours of discharge and responding to at least 50% of BP reminders). Completion was defined as participation throughout the 14 days without unenrolment. |
| Notes | Other findings reported in Sabol [33]  Funding source: none reported |

Saghir [92] poster

| Methods | Study design: prospective cohort study  Study duration: not reported  Study follow-up: 12 weeks |
| --- | --- |
| Participants | Country: UK  Setting: district general hospital  Number: 12  Education level: not reported  Mean age (years): not reported  Inclusion criteria: 12 consecutive postnatal patients diagnosed with preeclampsia on antihypertensives  Exclusion criteria: not reported |
| Interventions | Intervention type classification: self-monitoring  Intervention group: remote BP monitoring with tele device which prompts self-assessment of blood pressure, logs warning symptoms  Control group: nil |
| Outcomes | Primary outcome: Duration of monitoring, clinical outcomes, feasibility, safety, patient’s satisfaction and impact on secondary care |
| Notes | No other publications for this study identified  Funding source: none reported |

Sanghavi [34]

| Methods | Study design: retrospective cohort  Study duration: 6 months (March 1 2020- August 30 2020)  Study follow-up: 6 weeks |
| --- | --- |
| Participants | Country: USA  Setting: hospital  Number: 236 unique new patient visits (119 telemedicine visits, 117 control)  Education level: not reported  Mean age (years): intervention 30.8, control 30.5  Inclusion criteria: patients who deliver at Hospital of the University of Pennsylvania (HUP) and develop severe term preeclampsia requiring medications on discharge, preterm preeclampsia, or superimposed preeclampsia who require postpartum visits in the women’s cardiovascular health clinic  Exclusion criteria: duplicate patient encounters were excluded |
| Interventions | Intervention type classification: decision aid  Intervention group: telemedicine visit in postpartum clinic  Control group: retrospective cohort of patients with preeclampsia with in-person postpartum visits between March 1 2019- August 30 2019 |
| Outcomes | Primary outcome: compare completion rate of new patient telemedicine visits  to in-person office visits for patients with preeclampsia referred for postpartum hypertension management and CV risk assessment at HUP |
| Notes | No other publications for this study identified  Funding source: none reported |

Scalise [65] poster

| Methods | Study design: cohort study  Study duration: 3 months  Study follow-up: 1 week |
| --- | --- |
| Participants | Country: USA  Setting: outpatient clinic  Number: 123  Education level: not reported  Mean age (years): not reported  Inclusion criteria: patients attending postpartum hypertensive clinic  Exclusion criteria: not reported |
| Interventions | Intervention type classification: decision aid  Intervention group: text message clinical review  Control group: nil |
| Outcomes | Primary outcome: 7-day readmission rate, user satisfaction, user response rate |
| Notes | No other publications for this study identified  Funding source: none reported |

Sheth [111] poster

| Methods | Study design: case series  Study duration: not reported  Study follow-up: up to 41 weeks |
| --- | --- |
| Participants | Country: USA  Setting: outpatient clinic of medical centre  Number: 2  Education level: not reported  Mean age (years): not reported  Inclusion criteria: not reported  Exclusion criteria: not reported |
| Interventions | Intervention type classification: self-monitoring, decision aid (communication with clinician)  Intervention group: remote BP monitoring with communication via mobile phone app  Control group: nil |
| Outcomes | Primary outcome: clinical outcomes |
| Notes | No other publications for this study identified  Funding source: none reported |

Sheehan [112]

| Methods | Study design: qualitative study  Study duration: not reported  Study follow-up: 8 weeks |
| --- | --- |
| Participants | Country: UK  Setting: antenatal hypertension clinic in a London teaching hospital  Number: 8  Education level: not reported  Mean age (years): 35.38  Inclusion criteria: Participants  completed at least 8 weeks of HBPM using the innovative care pathway.  Exclusion criteria: not reported |
| Interventions | Intervention type classification: self-monitoring, decision aid (communication with clinician)  Intervention group: Remote BP monitoring with BP cuff with storage of data via smartphone application and educational video  Control group: nil |
| Outcomes | Primary outcome: women’s experiences of using home BP monitoring service |
| Notes | No other publications for this study identified  Funding source: The innovative care pathway that this research is based upon was funded by the Health Foundation. |

Socrates [93] poster

| Methods | Study design: prospective cohort study, nested substudy  Study duration: not reported  Study follow-up: 12 weeks |
| --- | --- |
| Participants | Country: Switzerland  Setting: hospital  Number: 112  Education level: not reported  Mean age (years): not reported  Inclusion criteria: preexisting hypertension, HDP, de novo postpartum hypertension  Exclusion criteria: not reported |
| Interventions | Intervention type classification: self-monitoring, decision aid (communication with clinician)  Intervention group: home based telemonitoring with smartphone app, remote BP monitoring with telephone consultations  Control group: standard care |
| Outcomes | Primary outcome: clinical outcomes, readmission rate, feasibility, acceptable, safety |
| Notes | No other publications for this study identified  Funding source: none reported |

Spiro [94] poster

| Methods | Study design: cohort study  Study duration: not reported  Study follow-up: 10 days |
| --- | --- |
| Participants | Country: USA  Setting: hospital  Number: 30  Education level: not reported  Mean age (years): not reported  Inclusion criteria: not reported  Exclusion criteria: not reported |
| Interventions | Intervention type classification: self-monitoring, decision aid (communication with clinician)  Intervention group: remote BP monitoring with data storage on web-based platform and telehealth communication with clinician  Control group: nil |
| Outcomes | Primary outcome: feasibility, patient satisfaction, adherence rate of 80% or higher  Secondary outcomes: readmissions, elevated BP, antihypertensive medication up-titration |
| Notes | No other publications for this study identified  Funding source: none reported |

Thomas [80]

| Methods | Study design: secondary analysis (cross-sectional post-participation web based) survey study) of non-randomised controlled trial Hoppe [75]  Study duration: original study duration - 14 months (April 2017-June 2018) – this survey administered at 6 weeks postpartum  Study follow-up: 6 weeks |
| --- | --- |
| Participants | Country: USA  Setting: hospital  Number: 128 participants completed survey (195 of original 214 participants completed initial study)  Mean age (years): survey respondents 32, intervention participants 31  Inclusion criteria: Inclusion criteria for the parent intervention study and  subsequently this survey were women admitted for delivery of their neonate with any of the following hypertensive diagnoses: chronic hypertension, gestational hypertension, preeclampsia or eclampsia  Exclusion criteria: not reported |
| Interventions | Intervention type classification: self-monitoring, decision aid (communication with clinician)  Intervention group: remote BP monitoring with tablet device and Bluetooth blood pressure cuff with telehealth review with clinician  Control group: standard outpatient care |
| Outcomes | Primary outcome: assess patient perspectives and experiences regarding daily postpartum blood pressure monitoring via telehealth with remote patient monitoring |
| Notes | Secondary analysis of Hoppe [75]  Funding source: none reported |

Tran [121]

| Methods | Study design: prospective cohort study  Study duration: 19 months (May 2020 – December 2021)  Study follow-up: up to 24 weeks (from recruitment (>20 weeks) to 4 weeks postpartum) |
| --- | --- |
| Participants | Country: Canada  Setting: hospital  Number: 103 (83 in final analysis)  Education level (n, %): Less than high school 1, 1.3, high school 7, 8.9, some college no degree 11, 13.9, college 27, 34.2, university 15, 19, professional qualifications 2, 2.5, Masters or PhD 16, 20.3  Median age (years): 35  Inclusion criteria: age ≥18 years old; English speaking; able to provide informed consent; greater than 20 weeks’ gestation; diagnosis of hypertensive disorder of pregnancy (chronic hypertension (SBP > 140 mm Hg or DBP > 90 mm Hg before 20 weeks gestation), gestational hypertension (SBP > 140 mm Hg or DBP > 90 mm Hg after 20 weeks gestation), or isolated office SBP or DBP reading greater than 140 or 90 mm Hg, respectively), with at least one additional  risk factors for developing preeclampsia (i.e.: previous history of preeclampsia,  anti-phospholipid antibody syndrome, pre-existing renal disease or booking proteinuria, preexisting diabetes, age ≥40 years, family history of preeclampsia (mother or sister) or early onset cardiovascular disease, multiple pregnancy, overweight/obesity, first pregnancy, pregnancy with a new partner, assisted reproductive technology, or interpregnancy interval ≥10 years)  were  Exclusion criteria: unable to perform HBPM or attend obstetric medicine (ObM) clinic visits |
| Interventions | Intervention type classification: self-monitoring, decision aid (communication with clinician)  Intervention group: remote BP monitoring with communication via email  Control group: nil |
| Outcomes | Primary outcome: describe how ObM physicians used and interpreted home BP measurements to manage clinical care. Secondary outcome: the proportion of patient visits where ObM physicians  utilized 7-day average home BP values versus home BP range versus last 3-day values to make clinical decisions on HDP  management. HBPM compliance was assessed by calculating the number of completed home BP readings in one week prior to clinic visit divided by  the expected number (the number of home BP readings requested). |
| Notes | No other publications for this study identified  Funding source: none reported |

Triebwasser [42]

| Methods | Study design: retrospective cohort study  Study duration: intervention 6 months (September 2018 – February 2019), control 6 months (August 2016 – January 2017)  Study follow-up: 10 days for BP measurement, up to 6 weeks for follow up |
| --- | --- |
| Participants | Country: USA  Setting: two hospitals  Number: intervention 333, control 103  Education level: not reported  Mean age (years): intervention 31.1, control 28.6  Inclusion criteria: women eligible for the trial were diagnosed with gestational hypertension (gHTN), pre-eclampsia (PEC), chronic hypertension with superimposed PEC, chronic hypertension without superimposed PEC, HELLP syndrome, or eclampsia at the time of their delivery admission over 18 years of age, be able to speak and read English, and have access to a cell phone with unlimited text message capabilities, control: as above excluding chronic hypertension without superimposed PEC  Exclusion criteria: not reported |
| Interventions | Intervention type classification: self-monitoring, decision aid (communication with clinician)  Intervention group: remote BP monitoring with text message communication and data storage on web based platform recruited from September 2018 – February 2019 at a second teaching hospital in same hospital network as control cohort  Control group: intervention cohort from Hirshberg [61] 2018 – same intervention as above recruited from August 2016 – January 2017 with implementation at a different hospital in same hospital network |
| Outcomes | Primary outcome: BP ascertainment, defined as the percentage  of patients in which a single BP was obtained in the first  10 days following discharge. Our secondary outcome was the proportion  of women meeting ACOG recommendations for postpartum BP  evaluation with BP sent on postpartum day 3–4 and again day 7–10 |
| Notes | Control cohort in this study was intervention cohort from Hirshberg [61] 2018  Funding source: this work was supported by the University of Pennsylvania  Department of Obstetrics and Gynecology Harrison Fund and the  Preeclampsia Foundation. |

Tucker [98] 2022 – BUMP 1 trial

| Methods | Study design: unblinded randomised clinical trial  Study duration: 12 months (November 2018 – October 2019)  Study follow-up: up to 36 weeks (recruitment from 16 weeks to 12 weeks postpartum) |
| --- | --- |
| Participants | Country: UK  Setting: 15 hospital maternity units  Number: 2441 total (recruited and randomised – 1223 intervention 1218 control), 2346 in final analysis (1171 intervention, 1175 control)  Education level (n, %): total reported intervention 1209 control 1201, tertiary education intervention 715, 59.1 control 684, 57, professional qualifications intervention 122, 10.1, control 120, 10, A-level of General Certificate of Secondary Education intervention 294, 24.3 control 335, 27.9, vocational qualifications intervention 34, 2.8 control 32, 2.7, no formal qualifications intervention 44, 3.6 control 30, 2.5  Mean age (years): intervention 32.8, control 33  Inclusion criteria: pregnant individuals at 16 to 24 weeks' gestation with higher risk of preeclampsia - higher risk included 1 or more of the following risk factors for pregnancy hypertension: age 40 years or older with a nulliparity pregnancy interval of greater than 10 years, family history of preeclampsia, history of preeclampsia or gestational hypertension, body mass index of 30 or greater, any stage of chronic kidney disease, twin pregnancy, prepregnancy diabetes, or autoimmune disease (eg, systemic lupus erythematosus or antiphospholipid syndrome).  Exclusion criteria: individuals with a preexisting diagnosis of hypertension |
| Interventions | Intervention type classification: self-monitoring, decision aid (communication with clinician)  Intervention group: remote BP monitoring with communication via mobile phone app and telehealth contact with clinician, storage of data on web based platform  Control group: usual care |
| Outcomes | Primary outcome: time to first recorded hypertension measured by a health care professional  Secondary outcomes: both maternal (severe hypertension [systolic BP ≥160 mm Hg and/or diastolic BP ≥110 mm Hg], serious maternal complications, and onset of labor) and perinatal (stillbirth and early neonatal death, gestation at delivery, mode of delivery, birth weight [including percentiles], small for gestational age [<10th and <3rd percentiles], and neonatal admissions). Patient-reported maternal outcomes were captured by questionnaires at baseline, 30 weeks’ gestation, and 12 weeks postnatally for illness perception (adapted Brief Illness Perception Questionnaire score range, 0-10; higher scores reflect greater confidence in ability to manage hypertension; minimal clinically important difference [MCID] not available), anxiety (6-item State-Trait Anxiety Inventory [STAI-6] scaled to 100; score range, 0 [no anxiety] to 100 [highest level of anxiety]; MCID = 10), and maternal health-related quality of life (EuroQol EQ-5D-5L; score range, −0.594 [worst quality] to +1 [best quality]; MCID = 0.037) |
| Notes | No other publications for this study identified  Funding source: This work was funded from a National Institute for Health Research (NIHR) Programme grant for applied research (RP-PG-1209-10051) and NIHR professorships awarded to Dr McManus (NIHR-RP-R2-12-015) and Dr Chappell (NIHR -RP-2014-05-019). Drs McManus and Tucker received funding from the National Institute for Health Research (NIHR) Collaboration for Leadership in Applied Health Research (CLAHRC) now recommissioned as NIHR Applied Research Collaboration Oxford and Thames Valley. Dr Mackillop received support from NIHR Oxford Biomedical Research Centre. Dr Hinton is based in The Healthcare Improvement Studies Institute (THIS Institute), University of Cambridge. THIS Institute is supported by the Health Foundation, an independent charity. Dr Sandall was supported by NIHR CLAHRC South London at King’s College Hospital NHS Foundation Trust, now recommissioned as NIHR Applied Research Collaboration South London. Dr Yardley’s research programme is partly supported by NIHR Applied Research Collaboration (ARC)-West, NIHR Health Protection Research Unit (HPRU) for Behavioural Science and Evaluation, and the NIHR Southampton Biomedical Research Centre (BRC). Drs McManus, Sandall, Yu, Yardley, and Chappell are NIHR Senior Investigators. Service support costs were administered through the NIHR Clinical Research Network. Dr Chappell reported serving as Chief Scientific Adviser to the UK Department of Health and Social Care and chief executive officer for NIHR. The BP monitors for the trial were purchased from the manufacturer (Microlife) at commercial prices. The BUMP app has been developed into a commercial product in collaboration with Sensyne Health and provided free to the NHS during the coronavirus pandemic through free licensing from both University of Oxford and Sensyne Health. |

Tucker [66] 2017

| Methods | Study design: prospective cohort  Study duration: 9 months (April 2013 – April 2014)  Study follow-up: up to 24 weeks (follow up from recruitment from 12 weeks to 36 weeks) |
| --- | --- |
| Participants | Country: UK  Setting: two hospital trusts and 13 primary care practices  Number: 201  Education level (n): professional qualifications or degree 101, school qualifications only 74, no formal qualifications 7, unknown 19  Mean age (years): 31  Inclusion criteria: pregnant women were those able and willing to self-monitor BP and higher risk of pre-eclampsia on the basis of any of the following risk factors; aged 40 years or older; nulliparity (first pregnancy); pregnancy interval of more than 10 years; family history of pre-eclampsia; previous history of pre-eclampsia; history of hypertension in pregnancy, body mass index of  30 kg/m2 or above at booking; pre-existing vascular disease such as hypertension; pre-existing renal disease; or multiple pregnancy  Exclusion criteria: not reported |
| Interventions | Intervention type classification: self-monitoring, decision aid (communication with clinician)  Intervention group: remote BP monitoring with communication via text message and storage of data on web based platform  Control group: nil |
| Outcomes | Primary outcome: numbers recruited and  retained, and persistence of self-monitoring. Retention was defined as the proportion of women recruited who  remained in the study until miscarriage or delivery or final follow-up at 36 weeks, whichever was longer. Persistence  was defined as the proportion of women who selfmonitored until miscarriage or delivery or final follow-up at 36 weeks, whichever was longer.  Secondary outcomes: the difference between mean self-monitored and clinic BP (systolic and diastolic); and the performance of self-monitored BP in the detection of gestational hypertension compared to the reference of clinic BP. |
| Notes | No other publications for this study identified  Funding source: This article represents independent research commissioned by the National Institute for Health Research (NIHR) School of Primary Care Research (SPCR) (SPCR project No. 171). The views expressed in this publication are those of the authors and not necessarily those of the NHS, the NIHR or the Department of Health. RM receives support from NIHR Professorship (NIHR-RP-02-12-015) and the NIHR Oxford Collaboration for Leadership in Applied Health Research and Care. SG is part funded by the National Institute for Health Research (NIHR) Collaboration for Leadership in Applied Health Research and Care West Midlands. During the period of the research LL was employed at the Nuffield Department of Primary Care Health Sciences and supported by the NIHR Oxford Biomedical Research Centre. |

Van den Heuvel [81] 2019

| Methods | Study design: prospective observational study  Study duration: one month (June 2017)  Study follow-up: 15 days (of remote BP monitoring program) patient opinion of program assessed 1 week after end of study period – total follow up 22 days |
| --- | --- |
| Participants | Country: Netherlands  Setting: outpatient clinic  Number: 14  Education level (n, %): unknown 2, 14, secondary education 3, 22, post-secondary education 9, 64  Mean age (years): 30.3  Inclusion criteria: Women between 18 and 40 years old with a gestational age < 34 weeks, if they could read and speak the Dutch language and had access to a smartphone or tablet with internet connection.  Exclusion criteria: chronic hypertension, hyper- tensive disorder in a prior pregnancy, cardiac or renal pathology, obesity (BMI > 35), or arm circumference > 42 cm |
| Interventions | Intervention type classification: self-monitoring, decision aid (communication with clinician)  Intervention group: remote BP monitoring with Bluetooth BP cuff and communication via app, storage of data via web based platform and communication with clinicians via telehealth  Control group: nil |
| Outcomes | Primary outcome: patient interaction and compliance - measured by registering the number of times patients sent their blood pressure and/or the checklist. Accuracy of the automatic alert system - evaluated by manual comparison of all entered values with the system thresholds for error positive or missing alerts. Clinical impact of the alert system - assessed through the submitted combination of BP and concurrent presence or absence of preeclampsia symptoms. Patient satisfaction and usability of the app and platform was examined one week after the end of the study period. |
| Notes | No other publications for this study identified  Funding source: the work described in this study was carried out in the context of the e-Health Citrien Program, which is part of and funded by the Dutch Federation of University Medical Centers (Nederlandse Federatie van Universitair Medische Centra, NFU). |

Vandenberk [120] 2019 (analysis of Lanssens [91] 2018)

| Methods | Study design: mixed method study of participants from retrospective cohort study - Lanssens [91] 2018  Study duration: 2 years (1/1/2015-31/12/2016) – this study recruited patients 1 day after inclusion in original study  Study follow-up: up to 90 days (from inclusion at ~ 10 weeks until delivery) |
| --- | --- |
| Participants | Country: Belgium  Setting: outpatient antenatal clinic  Number: 124 (108 in final analysis)  Education level: not reported  Median age (years): 30  Inclusion criteria: patients of original study PREMOM – study criteria (all women diagnosed with GHD who underwent prenatal follow-up at the outpatients prenatal clinic of Ziekenhuis Oost- Limburg (Genk, Belgium) during 2015 and 2016 were included)  Exclusion criteria: nil reported |
| Interventions | Intervention type classification: self-monitoring, decision aid (communication with clinician)  Intervention group: GHD at a gestational age of >10 weeks when intensive follow-up until delivery was desirable, remote BP self monitoring with wireless BP monitor and transmission of data to online platform with telehealth review by clinician  Control group: Women at a gestational age of <10 weeks, or women who did not give their informed consent received CC |
| Outcomes | Primary outcome: explore the roles of depression and anxiety, cognitive factors, and attachment and personality traits in relation to adherence to RM |
| Notes | This study included patients from Lanssens [91] 2018 - PREMOM  Funding source: none reported |

Van den Heuvel [15] 2020 – Safe@Home

| Methods | Study design: case control study  Study duration: 14 months (October 2017 and December 2018)  Study follow-up: up to 24 weeks (for duration of pregnancy, earliest BP monitoring from 16 weeks) |
| --- | --- |
| Participants | Country: Netherlands  Setting: two perinatal centres  Number: 103 in intervention group, 133 control  Education level (n, %): primary school or less intervention 3, 2.9 control 2, 1.5, high school or less intervention 9, 8.7 control 5, 3.8, secondary vocational school intervention 36, 35 control 44, 33.1, higher professional education intervention 30, 29.1 control 48, 36.1, university graduate intervention 25, 24.3 control 34, 25.6  Mean age: intervention 33.7, control 33.1  Inclusion criteria: women who presented with one (or more) of the following risk factors for preeclampsia: chronic hypertension, preeclampsia  in a prior pregnancy, maternal cardiac disease, or maternal kidney disease between October 2017 and  December 2018 in the two clinics. Eligible candidates for the prospective  study were>18 years of age, had access to a smartphone or tablet with  Internet connection and could understand Dutch or English language.  Exclusion criteria: kidney transplant patients and arm circumference>42 cm |
| Interventions | Intervention type classification: self-monitoring, decision aid (communication with clinician)  Intervention group: remote BP monitoring with Bluetooth BP cuff and transmission of data to web based portal with communication via mobile phone application or email  Control group: retrospectively selected women with one of the aforementioned four risk factors at start of pregnancy. After database search for these risk factors amongst all deliveries between 1  and 1-2015 and 31-12-2016, patients were included in this control group only if they received antenatal care from intake to delivery in the same centre. |
| Outcomes | Primary outcomes: healthcare consumption and user experiences  of the digital telemonitoring platform. For healthcare consumption, the number of antenatal visits, ultrasounds  for fetal assessment, blood and urinary analysis, medication use  and admissions were extracted  Secondary outcomes: maternal and neonatal perinatal outcomes |
| Notes | A mixed methods study on these patients experience and motivations in using the intervention was conducted by Jongsma [77].  Funding source: This research was funded by the e-Health Citrien Program of the Dutch Federation of University Medical Centers (Nederlandse Federatie  van Universitair Medische Centra, NFU). |

Van den Heuvel [35] 2021 – cost analysis of Van den Heuvel – [15] 2020

| Methods | Study design: analysis of original case control study [15]  Study duration: 14 months (October 2017 and December 2018)  Study follow-up: up to 24 weeks (for duration of pregnancy, earliest BP monitoring from 16 weeks) |
| --- | --- |
| Participants | Country: Netherlands  Setting: two perinatal centres  Number: 230 total (97 intervention, 133 control  Education level (n, %): primary school or less intervention 3, 3.1 control 2, 1.5, high school or less intervention 9, 9.3 control 5, 3.8, secondary vocational school intervention 35, 36.1 control 44, 33.1, higher professional education intervention 26, 26.8 control 48, 36.1, university graduate 24, 24.7 control 34, 25.6  Mean age: intervention 33.7, control 331.  Inclusion criteria: women who presented with one (or more) of the following risk factors for preeclampsia: chronic hypertension, preeclampsia in a prior pregnancy, maternal cardiac disease, or maternal kidney disease between October 2017 and December 2018 in the two clinics, >18 years of age, had access to a smartphone or tablet with  Internet connection and could understand Dutch or English language.  Exclusion criteria: kidney transplant patients and arm circumference>42 cm (as prescribed by the instructions of the monitor) |
| Interventions | Intervention type classification: self-monitoring, decision aid (communication with clinician)  Intervention group: remote BP monitoring with Bluetooth BP cuff and transmission of data to web based portal with communication via mobile phone application or email  Control group: retrospectively selected women with one of the aforementioned four risk factors at start of pregnancy. After database search for these risk factors amongst all deliveries between 1  and 1-2015 and 31-12-2016, patients were included in this control group only if they received antenatal care from intake to delivery in the same centre. |
| Outcomes | Primary outcomes: cost analysis of healthcare consumption from intervention |
| Notes | This was an alternate analysis of original study - Van den Heuvel [15] 2020 – Safe@Home  Funding source: This research was funded by the e-Health Citrien Program of the Dutch Federation of University Medical Centers (Nederlandse Federatie van Universitair Medische Centra, NFU). |

Winsten [36] poster

| Methods | Study design: prospective cohort study  Study duration: 4 months (April – July 2022)  Study follow-up: 14 days |
| --- | --- |
| Participants | Country: USA  Setting: outpatient clinic  Number: 64  Education level: not reported  Mean age: not reported  Inclusion criteria: not reported  Exclusion criteria: not reported |
| Interventions | Intervention type classification: self-monitoring  Intervention group: remote BP monitoring with data storage on web based platform, text message and telehealth communication with clinician  Control group: nil |
| Outcomes | Primary outcome: utilisation defined as logging a BP, viewing educational information, and/or interacting with a virtual nurse |
| Notes | No other publications for this study identified  Funding source: none reported |

Xydopoulos [113] – cost analysis of Perry [114]

| Methods | Study design: cost minimisation study of original case control study Perry [114]  Study duration: 3 years (December 2013 – November 2016)  Study follow-up: up to 9 weeks (from recruitment until delivery) |
| --- | --- |
| Participants | Country: UK  Setting: hospital  Number: 166 total (108 remote monitoring, 58 control)  Education level: not reported  Mean age (years): intervention 32.5, control 32  Inclusion criteria: chronic hypertension, GH or high risk of developing PE, no significant proteinuria (≤ 1+ proteinuria on dipstick testing), and normal biochemical and hematological markers, referred from either the hypertension clinic or to the DAU between December 2013 and November 2016  Exclusion criteria: not reported |
| Interventions | Intervention type classification: self-monitoring, decision aid (communication with clinician)  Intervention group: remote BP monitoring with data transmission via mobile phone app  Control group: retrospective cohort of patients managed with routine care with regular DAU visits for BP monitoring |
| Outcomes | Primary outcome: healthcare economic evaluation |
| Notes | Analysis of original case control study Perry [114]  Funding source: none reported |

Zizzo [99]

| Methods | Study design: retrospective cohort  Study duration: 8 years 11 months (February 2011 – December 2019)  Study follow-up: up to 6 weeks (follow up from inclusion where median gestational age at inclusion was 33.8 weeks to delivery) |
| --- | --- |
| Participants | Country: Denmark  Setting: hospital  Number: total 245 (PE and foetal growth restriction (FGR) 94, high risk of PE 151)  Education level: not reported  Mean age (years): PE and FGR 31, high risk of PE 32  Inclusion criteria: singleton pregnant women enrolled in the home monitoring program at Aarhus University Hospital, Denmark, between February 2011 and December 2019. Only native Danish-speaking or English-speaking women were included.  Exclusion criteria: nil |
| Interventions | Intervention type classification: decision aid  Intervention group: telemedicine outpatient management -daily evaluation of patients via telehealth  Control group: nil |
| Outcomes | Primary outcome: days include in home-monitoring, number of monitoring sessions and rehospitalisations  Secondary outcomes: obstetric and perinatal clinical outcomes |
| Notes | No other publications for this study identified  Funding source: none reported |

Abelman [43]

| Methods | Study design: retrospective cohort  Study duration: 14 months (April 2019 -October 2019 and April 2020 – October 2020)  Study follow up: not reported |
| --- | --- |
| Participants | Country: USA  Setting: 1 urban tertiary care centre  Number: 498 (control 231, intervention 267)  Education level: not reported  Mean age (years): control 29.23, intervention 30  Inclusion criteria: patients to have any diagnosis of an HDP before delivery (including gestational hypertension, preeclampsia with or without severe features, superimposed preeclampsia with or without severe features, or hemolysis, elevated liver enzymes, and low platelet count), delivery at our hospital, and prenatal care received at this same institution or an affiliated city-run health center.  Exclusion criteria: if their hypertensive diagnosis was made after delivery, if they did not receive prenatal care within this hospital system or an affiliated clinic, or if they transferred their prenatal care to this institution late in pregnancy (after 34 weeks of gestation or after already receiving a diagnosis of an HDP) |
| Interventions | Intervention type classification: decision aid  Intervention group: telehealth (cohort who delivered April 2020- October 2020)  Control group: retrospective cohort (April 2019 -October 2019) without telehealth (some telehealth used however not officially implemented) |
| Outcomes | The primary outcome examined was mean gestational age at which initial diagnosis of a hypertensive disorder was made, in cohorts before and after the implementation of telehealth protocols  The secondary outcomes included assessing the effect of severity of the hypertensive disorder at the time of diagnosis and at delivery. |
| Notes | No other publications for this study identified  Funding source: none reported |

Burgess [68] 2024

| Methods | Study design: retrospective cohort  Study duration: 22 months (17 September 2020 – 14 July 2022)  Study follow up: antenatal and postpartum education for up to 1 year, and BP monitoring for 16 days postpartum |
| --- | --- |
| Participants | Country: USA  Setting: five hospitals for intervention (three out of the five hospitals for control)  Number: control 812, intervention 837  Education level: not reported  Mean age (years): intervention 30  Inclusion criteria: all postpartum patients  who had been diagnosed with a hypertensive disorder  of pregnancy  Exclusion criteria: not reported |
| Interventions | Intervention type classification: self-monitoring, decision aid (communication with clinician)  Intervention group: antenatal and postpartum education via mobile phone app, remote self BP monitoring with communication via mobile phone app, text message or email, and telehealth review  Control group: historical cohort at 3 (of the 5 hospitals intervention utilised at) hospitals |
| Outcomes | Primary outcome: participation rate (BP measurement with automatic BP cuff), impact of implementation of remote patient monitoring on readmissions and ED utilisation  Secondary outcomes: patient satisfaction |
| Notes | No other publications for this study identified  Funding source: none reported |

Campbell [37] – cost analysis of BUMP trials (Tucker [98] – BUMP 1 trial, Chappell [104] – BUMP 2 trial)

| Methods | Study design: BUMP 1 – unblinded randomised clinical trial  BUMP 2 - non blinded randomised trial  Study duration: BUMP 1 – 12 months (November 2018 – October 2019)  BUMP 2 - 10 months (November 2018 - September 2019)  Study follow up: BUMP 1 – up to 36 weeks (recruitment from 16 weeks to 12 weeks postpartum)  BUMP 2 – up to 33 weeks (follow up from recruitment at booking in (earliest gestation 15 weeks) to 8 weeks after birth) |
| --- | --- |
| Participants | Country: UK  Setting: 15 hospital maternity units  Number: BUMP 1 - 2441 total (1223 intervention, 1218 control)  BUMP 2 - 850 (430 allocated to SMBP, 420 control), primary outcome available for 416 (SMBP) 405 (control)    Education level: not reported  Mean age (years): BUMP 1 - intervention 32.8, control 33  BUMP 2 - chronic hypertension SMBP 36 control 35.5, gestational hypertension SMBP 33.5, control 33.6  Inclusion criteria: BUMP 1 - pregnant individuals at 16 to 24 weeks' gestation with higher risk of preeclampsia - higher risk was defined by the relevant UK guidance at the time and included 1 or more of the following risk factors for pregnancy hypertension: age 40 years or older with a nulliparity pregnancy interval of greater than 10 years, family history of preeclampsia, history of preeclampsia or gestational hypertension, body mass index of 30 or greater, any stage of chronic kidney disease, twin pregnancy, prepregnancy diabetes, or autoimmune disease (eg, systemic lupus erythematosus or antiphospholipid syndrome).  BUMP 2 - individuals aged 18 years or older were eligible if they had chronic hypertension (defined as sustained systolic BP ≥140 mm Hg and/or diastolic BP ≥90 mm Hg, present at booking or before 20 weeks’ gestation, or receiving antihypertensive treatment outside pregnancy or at time of referral) and were recruited up to 37+0 weeks’ gestation, or gestational hypertension (defined as sustained systolic BP ≥140 mm Hg and/or diastolic BP ≥90 mm Hg after 20 weeks’ gestation) and were recruited at 20 to 37 weeks’ gestation  Exclusion criteria: BUMP 1 - individuals with a preexisting diagnosis of hypertension were excluded  BUMP 2 - individuals considered likely to deliver within 48 hours of eligibility assessment were excluded |
| Interventions | Intervention type classification: self-monitoring, decision aid (communication with clinician)  Intervention group: remote BP monitoring with communication via mobile phone app and telehealth contact with clinician, storage of data on web based platform  Control group: usual care |
| Outcomes | Primary outcome: cost analysis |
| Notes | Cost analysis of BUMP trials (Tucker [98] – BUMP 1 trial, Chappell [104] – BUMP 2 trial)  Funding source: This work was funded from a National Institute for Health and Care Research (NIHR) program grant for applied research (RP-PG-1209-10051) and NIHR professorships awarded to R.J. McManus (NIHR-RP-R2-12-015) and L.C. Chappell (NIHR-RP-2014-05-019). R.J. McManus and K.L. Tucker received funding from the NIHR Collaboration for Leadership in Applied Health Research now recommissioned as NIHR Applied Research Collaboration Oxford and Thames Valley. R.J. McManus and L.C. Chappell are NIHR Senior Investigators. Service support costs were administered through the NIHR Clinical Research Network. |

Charifson [44] poster

| Methods | Study design: retrospective cohort  Study duration: not reported  Study follow up: not reported |
| --- | --- |
| Participants | Country: USA  Setting: community practice  Number: 152  Education level: not reported  Mean age (years): not reported  Inclusion criteria: not reported, pregnant patients at a certain community practice in Texas  Exclusion criteria: not reported |
| Interventions | Intervention type classification: self-monitoring  Intervention group: remote BP monitoring with connected BP devices (automated recording and transmission of values to web based platform)  Control group: remote BP monitoring with manual reporting of values to web based platform |
| Outcomes | Primary outcome: number of BP measures recorded and patient adherence (at least 2 BP measures/ day for duration of pregnancy) |
| Notes | No other publications for this study identified  Funding source: none reported |

Duncan [38] poster

| Methods | Study design: prospective cohort study  Study duration: 6 months (October 2021- April 2022)  Study follow up: 6 weeks |
| --- | --- |
| Participants | Country: USA  Setting: hospital  Number: 545 (306 consented to data collection)  Education level: not reported  Mean age (years): not reported  Inclusion criteria: postpartum patients with a diagnosis of HDP at the University of Chicago between October 2021 and  April 2022.  Exclusion criteria: not reported |
| Interventions | Intervention type classification: self-monitoring  Intervention group: remote BP monitoring with electronic submission of data  Control group: nil |
| Outcomes | Primary outcome: determine remote BP monitoring BP trends |
| Notes | Alternative outcome reported in different publication (poster) [119]  Funding source: none reported |

Duncan [119] poster (2024)

| Methods | Study design: prospective cohort study  Study duration: 6 months (October 2021- April 2022)  Study follow up: 6 weeks |
| --- | --- |
| Participants | Country: USA  Setting: hospital  Number: 545 (306 consented to data collection)  Education level: not reported  Mean age (years): not reported  Inclusion criteria: postpartum patients with a diagnosis of HDP at the University of Chicago between October 2021 and  April 2022.  Exclusion criteria: not reported |
| Interventions | Intervention type classification: self-monitoring  Intervention group: remote BP monitoring with electronic submission of data  Control group: nil |
| Outcomes | Primary outcome: maternal, obstetric, follow-up, and readmission rates |
| Notes | Alternative outcome reported in different publication (poster) [38]  Funding source: none reported |

Hermawati [116]

| Methods | Study design: quantitative research in prospective cohort study  Study duration: 7-27 February 2023  Study follow up: intervention with follow up over 7 days, survey given over 21 days |
| --- | --- |
| Participants | Country: Indonesia  Setting: 2 community health centres  Number: 62 total (31 control 31 intervention)  Education level: intervention senior high school 16 college 15, control senior high school 14 college 17  Age: intervention >35 years old 5, 20-35 years old 26, control >35 years old 9 20-35 year olds 22  Inclusion criteria: pregnant women in the third trimester of pregnancy, having had at least one pregnancy check-up, having a smartphone and WhatsApp application, being able to read and write, and being willing to be a respondent.  Exclusion criteria: pregnancy with complications |
| Interventions | Intervention type classification: education tool  Intervention group: animated video related to preventing preeclampsia with survey carried out via Whatsapp  Control group: education via routine antenatal care activities at the community health centre |
| Outcomes | Primary outcome: identify the effect of animation videos on preeclampsia  prevention. To identify pregnant women's knowledge and motivation regarding pre-eclampsia prevention between intervention and control groups on pretest and post-test evaluation |
| Notes | No other publications for this study identified  Funding source: none reported |

Howard [69]

| Methods | Study design: retrospective cohort study  Study duration: 6 years 8 months (January 1, 2016- September 5, 2022)  Study follow up: 6 weeks |
| --- | --- |
| Participants | Country: USA  Setting: hospital  Number: 7952 total (intervention 3976, control 3976)  Education level: not reported  Mean age (years): intervention 30.9, control 30.9  Inclusion criteria: adult patients with a singleton pregnancy and 1 pregnancy  record who were eligible for enrollment in the remote BP monitoring program CMOM during the study period and  delivered after 20 weeks’ gestation at a participating hospital between January 1, 2016, and September 5, 2022, other inclusion criteria - prenatal visit with a provider whose primary clinic  offered CMOM, active pregnancy episode in the Epic (Epic Systems Corporation) electronic health record (EHR), gestational age between 10 weeks and 20 weeks at enrollment, and possession of a Bluetooth-enabled personal smartphone  (iOS or Android).  Exclusion criteria: not reported |
| Interventions | Intervention type classification: self-monitoring, decision aid (communication with clinician)  Intervention group: remote BP monitoring with Bluetooth BP cuff, communication via mobile phone application, text messages, storage of data on web based platform  Control group: usual care |
| Outcomes | Primary outcome: BP ascertainment and interval. BP ascertainment was defined as the average number of BP measurements during the prenatal and postpartum periods. BP interval was defined as the average time interval  in days between BP measurements during the prenatal and postpartum periods.  Secondary outcome: assessment of postpartum adherence -evaluating the proportion of patients diagnosed with a hypertensive disorder of pregnancy who checked their BP within 7 days of discharge from the hospital |
| Notes | No other publications for this study identified  Funding source: none reported |

Irani [41] poster

| Methods | Study design: qualitative study  Study duration: not reported  Study follow up: not reported |
| --- | --- |
| Participants | Country: Ghana  Setting: urban tertiary hospital  Number: 55 participants  Education level: Formal education ranged from 4 with no education to 18 with tertiary education  Mean age (years): not reported  Inclusion criteria: adult pregnant women at a tertiary hospital in Accra, Ghana  Exclusion criteria: not reported |
| Interventions | Intervention type classification: self-monitoring  Intervention group: remote BP monitoring, audiovisual training of the use of the remote BP monitor and recognition of abnormal BP  Control group: nil |
| Outcomes | Primary outcome: perceived and objective numeracy to identify elevated BP values |
| Notes | No other publications for this study identified  Funding source: none reported |

Irani [122] poster – family member interviews

| Methods | Study design: qualitative study (of prospective cohort study)  Study duration: not reported  Study follow up: not reported |
| --- | --- |
| Participants | Country: Ghana  Setting: urban tertiary hospital  Number: 15 interviews  Education level: support person education level 86 % finished senior high school or more education,  Mean age (years): support person age range 21-59  Inclusion criteria: not reported  Exclusion criteria: not reported |
| Interventions | Intervention type classification: self-monitoring  Intervention group: remote BP monitoring, interviews of patient support person via telephone  Control group: nil |
| Outcomes | Primary outcome: exploration of support people’s perspectives on home blood pressure monitoring, including overall  experience, involvement, difficulties or challenges, perceived health benefits or risks, value of family support, and views on women’s health autonomy |
| Notes | No other publications for this study identified  Funding source: none reported |

Jones [39]

| Methods | Study design: mixed methods study (survey, semi-structured qualitative interview) of cohort study  Study duration: not reported  Study follow up: 8 weeks |
| --- | --- |
| Participants | Country: USA  Setting: hospital  Number: 30 (20 completed follow up, 10 lost to follow up)  Education level: 9^th^-12^th^ grade 2, high school graduate or GED 6, college or technical school 9, college graduate or higher 3  Mean age (years): not reported  Inclusion criteria: Participants included women aged >18 years whose pregnancies were complicated by hypertension and who received their prenatal care at the UAMS Women’s Health Clinic. Participants must have had elevated BP meeting the criteria of  systolic >/=140 and/or diastolic >/=90 for at least 1 reading.  Exclusion criteria: not reported |
| Interventions | Intervention type classification: self-monitoring, decision aid (communication with clinician)  Intervention group: remote BP monitoring with cellular enabled BP devices supported by telehealth communication and storage of data on web based platform  Control group: nil |
| Outcomes | Primary outcome: patient perception of remote BP monitoring |
| Notes | No other publications for this study identified  Funding source: This research was funded by the Translational Research Institute at the University of Arkansas for Medical Sciences, grant TR003107-02S4. |

Kitt [84] 2023

| Methods | Study design: randomized clinical trial  Study duration: 22 months (21 February 2020 – 2 November 2021)  Study follow up: 9 months |
| --- | --- |
| Participants | Country: UK  Setting: one hospital  Number: 220 total (intervention 112, control 108) – 200 in final analysis (intervention 105, control 95)  Education level: not reported  Mean age (years): 32.6 (intervention 33.4, control 32.6)  Inclusion criteria: aged 18 years or older, following pregnancy complicated by preeclampsia or gestational hypertension, requiring antihypertensive medication postnatally when discharged  Exclusion criteria: not reported |
| Interventions | Intervention type classification: self-monitoring, decision aid  Intervention group: remote blood pressure monitoring Bluetooth BP cuff with communication with clinician via mobile phone app, storage of data on web based platform  Control group: conventional care (no eHealth) |
| Outcomes | Primary outcome: 24-hour mean diastolic blood pressure at 9 months postpartum, adjusted for baseline postnatal blood pressure.  Secondary outcomes: adverse event monitoring, physical activity monitoring, readmission rates, quality of life (assessed via questionnaire) |
| Notes | No other publications for this study identified  Funding source: this study was funded by grant FS/19/7/34148, a BHF Clinical Research Training Fellowship to Dr Kitt with additional support from the NIHR Oxford Biomedical Research Centre and Oxford BHF Centre for Research Excellence |

Kumar [100]

| Methods | Study design: retrospective cohort study  Study duration: 4 months (March 16, 2019 - June 16, 2019)  Study follow up: 8 weeks |
| --- | --- |
| Participants | Country: USA  Setting: urban tertiary centre with 2 clinical sites  Number: 1579 total (intervention 799, control 780)  Education level: intervention high school or less 459 greater than high school 328, control high school or less 443, greater than high school 320  Mean age (years): intervention 30.2, control 30.3  Inclusion criteria: all patients with a scheduled postpartum visit of any modality at an outpatient obstetrical clinic affiliated with the tertiary care centre  Exclusion criteria: none |
| Interventions | Intervention type classification: decision aid (communication with clinician)  Intervention group: telehealth postpartum visits  Control group: historical cohort prior to telehealth visits (face to face) |
| Outcomes | Primary outcome: postpartum clinic visit attendance – to assess the impact of telemedicine implementation on racial disparities in postpartum care  Secondary outcome: postpartum depression screen completion rates, contraception choice, feeding plan, follow up for other testing (FGTT, pap smear, colposcopy, cardiology) – assessing if telehealth reduced racial disparities |
| Notes | No other publications for this study identified  Funding source: none reported |

Lemon [70]

| Methods | Study design: retrospective cohort study  Study duration:4 years 3 months (March 2019 - June 2023)  Study follow up: 6 weeks |
| --- | --- |
| Participants | Country: USA  Setting: hospital  Number: 12,038 total (6556 intervention, 5482 control)  Education level: not reported  Mean age (years): not reported  Inclusion criteria: diagnosis of HDP during the delivery admission  (preexisting or pregnancy-induced), or at least two inpatient postpartum BPs higher than 140/90 mm Hg during the delivery hospitalization. Participants  must speak English, Spanish, or Portuguese, and have a cellular device that accepts text messages.  Exclusion criteria: not reported |
| Interventions | Intervention type classification: self-monitoring, decision aid (communication with clinician)  Intervention group: remote BP monitoring with text message communication with data storage on web based platform and telehealth review  Control group: usual care |
| Outcomes | Primary outcome: postpartum care utilization, including hospital  (readmission and ED visits) along with attendance of a postpartum office visit within 6 weeks postpartum  Secondary outcomes: measurement of BP within 10 days of delivery and initiation of antihypertensive medications in the postpartum period, BP trajectories in the intervention group |
| Notes | No other publications for this study identified  Funding source: none reported |

Lewkowitz [45] – research letter

| Methods | Study design: prospective cohort study  Study duration: not reported  Study follow up: 6 weeks |
| --- | --- |
| Participants | Country: USA  Setting: hospital  Number: 119 total (59 intervention 60 control)  Education level: not reported  Mean age (years): not reported  Inclusion criteria: individuals with chronic or pregnancy-related hypertension are routinely enrolled into remote BP monitoring  Exclusion criteria: not reported |
| Interventions | Intervention type classification: self-monitoring, decision aid (communication with clinician)  Intervention group: remote BP monitoring with Bluetooth BP cuff with data transmission via mobile phone application utilising AI to respond to BPs or symptoms with communication with clinicians via email  Control group: remote BP monitoring with manual submission of data and usual care |
| Outcomes | Primary outcome: >/=1 BP assessment </=10 days postpartum  Secondary outcomes: remote BP medication initiation/titration and emergency department (ED) presentation or inpatient readmission within 30 days of discharge for HTN-related etiologies, participant experience of remote BP monitoring |
| Notes | No other publications for this study identified  Funding source: A.K.L. is supported by the NICHD (K23HD103961). A.H. is supported by the NHLBI (K23HL168356). This study was also supported by a grant  from the CVS Foundation/Essential Hospitals Institute. |

Mujic [71]

| Methods | Study design: prospective cohort study  Study duration: 17 months (January 2021 – May 2022)  Study follow up: 6 weeks |
| --- | --- |
| Participants | Country: USA  Setting: hospital  Number: 1118 patients received remote BP cuff, 1033 provided at least 2 BP measurements and were included in the analysis  Education level: not reported  Mean age (years): 30.9  Inclusion criteria: those delivering at ≥20 weeks gestation, including stillbirths and livebirths, with either chronic hypertension, gestational hypertension, preeclampsia, or de novo postpartum hypertension identified during the delivery hospitalization  Exclusion criteria: patients enrolled in the RBPM program from April to December 2020, as the data in these initial months of the program were not systematically integrated with electronic medical record data. |
| Interventions | Intervention type classification: self-monitoring, decision aid (communication with clinician)  Intervention group: remote BP monitoring with cell-enabled BP cuff with communication via text message and telehealth, data transmission to web based platform  Control group: nil |
| Outcomes | Primary outcome: frequency of use, defined as number of BP measurements ascertained, and duration of participation in the program, defined as day of last measurement during the 6-week (42-day) program.  Secondary analysis: postpartum BP ranges |
| Notes | No other publications for this study identified  Funding source: This work was supported by the National Institutes of Health (R01 HL158864). |

Nadkarni [40]

| Methods | Study design: retrospective cohort study  Study duration: 3 months (January – March 2021)  Study follow up: not reported |
| --- | --- |
| Participants | Country: USA  Setting: tertiary hospital  Number: 721 total  Education level: not reported  Mean age (years): not reported  Inclusion criteria: patients with one or more risk factors for preeclampsia -  including advanced maternal age (‡35), black race, body mass index ‡30, nulliparity, multiple gestation, Medicaid insurance, type 1 or type 2 diabetes, renal disease, autoimmune illness, chronic hypertension, and a prior diagnosis of preeclampsia or eclampsia.  Exclusion criteria: not reported |
| Interventions | Intervention type classification: decision aid  Intervention group: telehealth review  Control group: nil |
| Outcomes | Primary outcome: assess how many telehealth visits, blood pressure cuff access and documentation of self-measured BP occurred for patients with risk factors for preeclampsia |
| Notes | No other publications for this study identified  Funding source: none reported |

Runesha [85]

| Methods | Study design: descriptive analysis (survey) of cohort study  Study duration: 7 months (October 2021 – April 2022)  Study follow up: 6 weeks |
| --- | --- |
| Participants | Country: USA  Setting: hospital  Number: 306  Education level: not reported  Mean age (years): 31  Inclusion criteria: patients with HDP all enrolled into remote monitoring as standard care between October 2021 – April 2022  Exclusion criteria: not reported |
| Interventions | Intervention type classification: self-monitoring, decision aid (communication with clinician)  Intervention group: remote BP monitoring with Bluetooth BP cuff, communication with mobile phone app and telehealth clinician review  Control group: nil |
| Outcomes | Primary outcome: survey of remote BP monitoring participants to assess perceptions, knowledge and satisfaction |
| Notes | No other publications for this study identified  Funding source: the STAMPP–HTN program is funded through the UCM  health equity initiatives through IT Strategic Programs and Department  of Obstetrics and Gynecology at the University of Chicago medicine |

Tully [101]

| Methods | Study design: RCT (two arm)  Study duration: 14 months (September 2018 – November 2019)  Study follow up: 10 days |
| --- | --- |
| Participants | Country: USA  Setting: tertiary level hospital  Number: 100 (50 intervention 50 control)  Education level: intervention >high school 42, control 41  Mean age (years): intervention 29.8, control 28.6  Inclusion criteria: individuals who delivered at North Carolina Women’s Hospital on or after 20 weeks gestation, or were admitted within 3 days of delivery and was aged at least 18 years, with diagnosis of hypertensive disorder of pregnancy or chronic hypertension  requiring medications, and able to communicate in English or Spanish.  Exclusion criteria: if they were less than 18 years of age, had a diagnosis of chronic hypertension not requiring medications during pregnancy or postpartum or no diagnosis of HDP, or reported that they lacked access to a cellular phone. |
| Interventions | Intervention type classification: self-monitoring, decision aid (communication with clinician)  Intervention group: remote BP monitoring with telephone review with clinician  Control group: standard care |
| Outcomes | Primary outcome: the proportion of  participants who underwent a BP evaluation at 7–10 days postpartum that was documented in the HER  Secondary outcomes: birthing parent acceptability of the postpartum |
| Notes | No other publications for this study identified  Funding source: This study was funded by the UNC Center for Health Innovation. |

Zhang [86] – brief communication

| Methods | Study design: prospective cohort study  Study duration: 20 months (9 July 2021 – 23 February 2023)  Study follow up: up to 23 weeks (recruited at mean of 21 weeks, follow up to 4 weeks postpartum) |
| --- | --- |
| Participants | Country: USA  Setting: hospital  Number: 98 (enrolled) 77 actively participated  Education level: not reported  Mean age (years): 30.53  Inclusion criteria: Adult patients with an intrauterine pregnancy, receiving prenatal care at UMMC and affiliated practices, proficient in English, and having reliable internet access  Exclusion criteria: not reported |
| Interventions | Intervention type classification: self-monitoring, decision aid (communication with clinician)  Intervention group: remote BP monitoring with Bluetooth BP cuff, communication via cellular enabled iPad tablet and telehealth and storage of data via web based platform  Control group: nil |
| Outcomes | Primary outcome: feasibility, adherence to protocol  Secondary outcomes: engagement activities, pregnancy outcomes |
| Notes | No other publications for this study identified  Funding source: This work is supported by the Office for the Advancement of  Telehealth, Health Resources and Services Administration, U.S. Department of Health and Human Services under cooperative agreement award no. 5U66RH31459-05-00. |
